# Accelerated Biological Aging, Genetic Predisposition, and Incident Valvular Heart Disease

**DOI:** 10.1016/j.jacasi.2025.06.011

**Published:** 2025-08-16

**Authors:** Yong-Jian Zhu, Xiang-Ying Suo, Jing Guo, Shuo Lu, Jun-Xi Zhang, Ya-Cong Bo, Zhan-Ying Han, Chun-Guang Qiu

**Affiliations:** a Department of Cardiology, The First Affiliated Hospital of Zhengzhou University, Zhengzhou, China; b School of Public Health, Zhengzhou University, Zhengzhou, China; c School of Physical Education (Main Campus), Zhengzhou University, Zhengzhou, China; d NHC Key Laboratory of Birth Defects Prevention, Henan Key Laboratory of Population Defects Prevention, Zhengzhou, China

**Keywords:** biological age, genetic predisposition, KDM-BA acceleration, PhenoAge acceleration, valvular heart disease

## Abstract

**Background:**

The association between biological aging and valvular heart disease (VHD) has not yet been evaluated.

**Objectives:**

This study aimed to evaluate the associations between 2 biological age indicators, Klemera–Doubal method biological age (KDM-BA) acceleration and PhenoAge acceleration, and the risk of VHD, as well as explore the potential gene–environment interplay.

**Methods:**

The study included 341,460 UK Biobank participants without VHD at enrollment. Biological age was assessed using KDM-BA and PhenoAge methods. Genetic risk was measured by genome-wide-association study–based polygenic risk scores (PRS). Cox models were used to assess the individual and joint effects of biological age and PRS on incident VHD. Both multiplicative and additive interactions between the 2 factors were also estimated.

**Results:**

During a median follow-up of 13.58 years (Q1-Q3: 12.83-14.25 years), 8,146 VHD cases were documented. The results showed a significant association between older biological age and an increased risk of VHD, with a HR of 1.35 (95% CI: 1.32-1.38) for each 1-SD increase in KDM-BA acceleration, and 1.29 (95% CI: 1.26-1.32) for PhenoAge acceleration. Compared with individuals in the first quartile group (Q1) for KDM-BA acceleration, those in Q4 showed the highest risk of VHD, with an 86% higher risk (HR: 1.86; 95% CI: 1.74-1.99). There was an additive interaction between KDM-BA acceleration and PRS for VHD. Similar results were found for the association between PhenoAge acceleration and VHD.

**Conclusions:**

Advanced biological aging was significantly associated with an increased risk of VHD and could serve as a potential target for clinical prediction and intervention.

Valvular heart disease (VHD) is a global issue with significant regional differences, and it remains a major source of mortality and disability worldwide,^1^ exerting substantial emotional and economic impacts on families and society. Aortic stenosis (AS) is the most common form of VHD, with its prevalence increasing in developed countries due to an aging population.^2^^,^^3^ However, surgical or transcatheter interventions are currently the primary treatment for VHD, as there is a lack of effective pharmacological therapies, limiting access to adequate treatment.^4^ Therefore, early recognition and a deeper understanding of the risk factors for VHD are imperative.

Aging is widespread and a crucial cardiovascular risk factor.^5^ Each organism has a different rate of aging, and subsequently, it is more important to focus on biological age rather than chronological age. Objective quantification of disparity between these can reflect the impact of age acceleration or deceleration, and evaluate the contribution of biological aging to the disease process.^6^ Previous studies preferred single indicators of biological age (such as telomere length or DNA methylation age),^7^^,^^8^ but recent studies have underscored integrative biological age biomarkers.^9^, ^10^, ^11^ The Klemera–Doubal method biological age (KDM-BA) and the PhenoAge algorithm offer solutions by using multiple clinical indicators to assess the biological age comprehensively.^12^^,^^13^ Both methods have been shown to be effective in predicting the onset of disease, disability, and mortality in multiethnic older cohorts.^14^^,^^15^ They have also been validated using data from the UK Biobank.^9^, ^10^, ^11^ In addition, 12 hallmarks of aging were proposed by Carlos López-Otín et al,^6^ which mostly overlap with mechanisms of vascular aging.^4^ However, only 2 cohort studies have investigated the link of biological age calculated by KDM-BA and the PhenoAge algorithm with heart failure and cardiometabolic multimorbidity.^10^^,^^16^ Studies remain scarce on the relationship between comprehensive biological age and VHD.

Previous findings indicated that genetic polymorphisms may play a role in onset of VHD,^17^^,^^18^ and biological age–genetic interactions have been found in mental disorders and chronic respiratory diseases.^9^^,^^11^ Yet there is limited understanding of how genetic predisposition might influence the association of biological age with VHD.

To address these gaps, we utilized data based on the UK Biobank to explore the prospective association between biological age measured by KDM-BA and PhenoAge, and incident VHD. Moreover, we assessed the joint effect and interaction of biological age and genetic predisposition on the development of VHD.

## Materials and Methods

### Study population

A detailed study design of the UK Biobank is outlined.^19^ Briefly, the UK Biobank is a prospective, largescale, population-based cohort study that enrolled more than 500,000 participants aged 40 to 74 years through 22 assessment centers distributed across England, Wales, and Scotland in 2006 to 2010. The study collected a wide range of data, including exposures, health status, biological and imaging data through self-reported questionnaires, biological samples, and physical examinations. All UK Biobank participants provided their informed written consent, and the UK Biobank has received ethical approval from the North West Multicenter Research Ethics Committee.

Supplemental Figure 1 depicts the procedure for participants selection. Among the 503,371 participants enrolled in 2006 to 2010, we firstly excluded 160,485 participants who lacked trait data for biological age algorithms or diagnosis of VHD. Furthermore, we excluded participants who refused to participate in follow-up and those with VHD at baseline (n = 992). Next, we excluded individuals who had congenital valvular malformations (including congenital malformations of pulmonary and tricuspid valves, or of aortic and mitral valves) before the end of the study (n = 434). In total, 341,460 individuals remained in the final analysis.

### Assessment of biological ages and age accelerations

Biological age was assessed using 2 of the most widely validated algorithms, KDM-BA and PhenoAge, based on blood chemistry data available from the UK Biobank dataset, as validated in previous studies.^9^^,^^12^^,^^14^ In brief, KDM-BA was computed using 2 physical parameters (forced expiratory volume in 1 second [FEV1] and systolic blood pressure), and 7 blood biomarkers (albumin, alkaline phosphatase, blood urea nitrogen, creatinine, C-reactive protein, glycated hemoglobin, and total cholesterol); PhenoAge was computed using 9 blood biomarkers including albumin, alkaline phosphatase, creatinine, C-reactive protein, glucose, mean cell volume, red cell distribution width, white blood cell count, and lymphocyte proportion. The physical parameters and blood biomarker that were included, along with their corresponding data fields from the UK Biobank, are listed in Supplemental Table 1. The biological age was computed using the “BioAge” R package.^20^ The methods of computation are detailed described in previous literature and Supplemental Methods.^9^

Furthermore, we regressed the biological ages against chronological age at the time of biomarker measurement and obtained the residuals. Consistent with a previous study,^9^ these residuals are referred to as “age acceleration (AA)” values, which reflect biological aging. The AA values for the 2 methods are termed “KDM-BA acceleration” and “PhenoAge acceleration,” respectively. To facilitate comparisons of effect sizes between the 2 biological aging measures, we standardized the AA values, setting the mean to 0 and the SD to 1 for continuous analysis. In addition, both KDM-BA acceleration and PhenoAge acceleration were categorized into 4 quartiles (quartile 1 [Q1]; quartile 2 [Q2], quartile 3 [Q3], and quartile 4 [Q4]) for dose–response analysis.

### Ascertainment of VHD

The outcome in the present study was incident degenerative VHD, including AS, aortic valve regurgitation (AR), and mitral valve regurgitation (MR). These conditions were determined based on the International Classification of Diseases coding system-10 (ICD-10) codes: I35.0 and I35.2 for AS, I35.1 for AR, and I34.0 for MR, which are described in Supplemental Table 2.^2^^,^^21^ Data were collected from hospital inpatient records, and death registry. Hospital admissions data were available through October 2022 for England, August 2022 for Scotland, and May 2022 for Wales. Participants were followed up until the occurrence of VHD, death, loss to follow-up, or the end of the study period, whichever occurred first.

### Genetic predisposition of VHD

We constructed a polygenic risk score (PRS) based on genome-wide association study (GWAS) summary statistics for VHD to quantify the genetic predisposition of each individual to the condition using genotype data from deCODE genetics.^22^ Single nucleotide polymorphisms (SNPs) were removed due to variants with a call rate <98%, a minor allele frequency <0.01, or deviation from Hardy-Weinberg equilibrium (*P* < 10^−8^). Individuals with no more than 10 estimated third-degree relatives based on the kinship table were included. The resulting PRS was classified into high genetic risk (highest quintile), intermediate genetic risk (quintiles 2-4), and low genetic risk (lowest quintile) for further analysis.

### Covariates

Covariates were obtained at enrollment and included age, sex (male/female), ethnicity (White/other), employment status (yes/no), education level (college or university degree/other), smoking status (never/ever/now), drinking status (never/ever/now), physical activity level, Townsend Deprivation Index, and body mass index (BMI). Physical activity levels were classified into 3 categories—low, moderate, and high—based on MET-min/week cutoffs of 600 and 3,000, which were obtained from the International Physical Activity Questionnaire (IPAQ). BMI was calculated by dividing weight in kilograms by the square of height in meters.

### Statistical analysis

Continuous variables were summarized as either the mean ± SD or median (IQR), depending on the distribution of the data. Categorical variables were presented as numbers (percentages). The missing data pattern is available in Supplemental Figure 2 and Supplemental Table 3, showing the data meet the missing at random assumption. Missing values of covariates were imputed by multiple imputation using the “mice” package in R (R Foundation for Statistical Computing). Five multiplely imputed datasets with 20 iterations each were generated to ensure convergence and stability of the imputation process. The imputation procedure was properly accounted for in the statistical modeling using Rubin’s rules.

Cox proportional hazard model was performed to assess the relationship between biological age and the incidence of VHD. The Schoenfeld residuals method was used to verify the proportional hazards assumption, which confirmed that no violations were detected. The population attributable fraction was calculated to estimate the proportion of VHD cases attributable to biological ages. Exposure–response relationships between biological ages and VHD were examined using restricted cubic spline regression. Three knots were placed at the 10th, 50th (reference), and 90th percentiles of the exposure distribution. Linearity was assumed at the lower and upper 10% of the exposure range. Departure from linearity was assessed using a Wald test. The covariates mentioned earlier were adjusted for in all models.

Next, individuals were categorized into 3 genetic risk groups—low, intermediate and high genetic risk—according to their PRS. We performed a joint analysis of their association with incident VHD in 3 × 4 groups, using individuals with the lowest quartile biological age and low genetic risk as the reference group. The relative excess risk of interaction (RERI) and the attributable proportion were further calculated to quantify additive interactions. In addition, separate analyses were then conducted for each genetic risk group to assess the associations of biological age with VHD across different genetic risk backgrounds.

We further investigate the potential modifying effects of age (<65 or ≥65 years), sex (male or female), ethnicity (white or other), employment status (yes or no), BMI (<25 or ≥25 kg/m^2^), smoking status (never, ever, now), drinking status (never, ever, now), and physical activity levels (low, moderate, high) underlying the association between biological age and VHD.

Furthermore, 5 sensitivity analyses were conducted to evaluate the robustness of these relationships. First, analyses were restricted to participants with complete covariate data. Second, we performed analyses excluding individuals who developed VHD in the first 2 years of follow-up to control for reverse causation. Third, we used a broader definition of VHD as the outcome,^23^ described in Supplemental Table 2, and reconducted the analyses. Fourth, comorbidities including hypertension, stroke, atrial fibrillation, chronic kidney disease, cancer, and type 2 diabetes were further adjusted as confounding factors in models. Finally, we reclassified biological ages, defining KDM-BA acceleration/PhenoAge acceleration >0 as biologically older and ≤0 as biologically younger than chronological age. These classifications were then used as exposures to evaluate the link of biological age with incident VHD.

All statistical analyses were performed by R software version 4.2.2. A *P* <0.05 on a 2-tailed test was considered statistically significant.

## Results

Among 341,460 individuals free of VHD at baseline (mean age: 56.89 ± 8.09 years; 54.1% [184,603/341,460] female), the cumulative incidence over a median follow-up of 13.58 years (Q1-Q3: 12.83-14.25 years) was 2.39% (8,146/341,460; 95% CI: 2.33%-2.44%) for VHD, 1.02% (3,479/341,460; 95% CI: 0.99%-1.05%) for AS, 0.38% (1,283/341,460; 95% CI: 0.36%-0.40%) for AR, and 1.18% (4,040/341,460; 95% CI: 1.15%-1.22%) for MR. The characteristics of participants by VHD status are presented in Table 1. Briefly, participants with VHD were more likely to be older (both chronological and biological ages), male, unemployed, and smokers, had higher BMI, lower income, and low physical activity. The similar population characteristics were also observed in individuals by AS, AR, and MR status (Supplemental Tables 4 to 6). Demographic features by biological ages (quartiles) are detailed in Supplemental Tables 7 and 8 and the characteristics of components of biological ages by VHD status are listed in Supplemental Table 9.

**Table 1:** Baseline Characteristics of Participants by VHD Status

	Overall (N = 341,460)	Non-VHD (n = 333,314)	VHD (n = 8,146)
Age, y	56.89 ± 8.09	56.75 ± 8.08	62.43 ± 6.17
Female	184,603 (54.1)	181,265 (54.4)	3,338 (41.0)
White	323,205 (94.7)	315,381 (94.6)	7,824 (96.0)
Income			
<£18,000	81,207 (23.8)	78,394 (23.5)	2,813 (34.5)
£18,000 to £30,999	87,942 (25.8)	85,570 (25.7)	2,372 (29.1)
£31,000 to £51,999	87,527 (25.6)	85,763 (25.7)	1,764 (21.7)
£52,000 to £100,000	67,167 (19.7)	66,208 (19.9)	959 (11.8)
>£100,000	17,617 (5.2)	17,379 (5.2)	238 (2.9)
Education level			
College or university degree	112,295 (32.9)	110,167 (33.1)	2,128 (26.1)
Others	229,165 (67.1)	223,147 (66.9)	6,018 (73.9)
Employment status			
No	140,246 (41.1)	135,081 (40.5)	5,165 (63.4)
Yes	201,214 (58.9)	198,233 (59.5)	2,981 (36.6)
Physical activity			
Low	63,324 (18.5)	61,673 (18.5)	1,651 (20.3)
Moderate	139,163 (40.8)	135,930 (40.8)	3,233 (39.7)
High	138,973 (40.7)	135,711 (40.7)	3,262 (40.0)
Smoking status			
Never	188,775 (55.3)	185,021 (55.5)	3,754 (46.1)
Ever	117,640 (34.5)	114,147 (34.2)	3,493 (42.9)
Current	35,045 (10.3)	34,146 (10.2)	899 (11.0)
Drinking status			
Never	14,664 (4.3)	14,305 (4.3)	359 (4.4)
Ever	11,614 (3.4)	11,199 (3.4)	415 (5.1)
Current	315,182 (92.3)	307,810 (92.3)	7,372 (90.5)
BMI, kg/m^2^	27.34 ± 4.69	27.30 ± 4.67	28.65 ± 5.12
Townsend deprivation index	−1.37 ± 3.05	−1.37 ± 3.05	−1.27 ± 3.15
Biological ages			
KDM-BA	54.29 ± 9.30	54.12 ± 9.27	61.36 ± 7.95
KDM-BA acceleration	−0.01 ± 4.83	−0.05 ± 4.81	1.63 ± 5.61
PhenoAge	47.92 ± 10.00	47.73 ± 9.95	55.55 ± 8.96
PhenoAge acceleration	−0.01 ± 5.36	−0.05 ± 5.32	1.73 ± 6.29

Values are mean ± SD or n (%).

BMI = body mass index; KDM-BA = Klemera–Doubal method biological age; VHD = valvular heart disease.

The relationships between biological aging and the risk of VHD, AS, AR, and MR are shown in Table 2 and Supplemental Tables 10 to 12. The results suggested that each 1-SD increased KDM-BA acceleration is linked to an increased risk of incident VHD (HR: 1.35; 95% CI: 1.32-1.38), AS (HR: 1.49; 95% CI:1.45-1.54), AR (HR: 1.24; 95% CI: 1.17-1.31), and MR (HR: 1.27; 95% CI: 1.23-1.31). Similarly, each 1-SD increased PhenoAge acceleration is also notably associated with higher risk of VHD (HR: 1.29; 95% CI: 1.26-1.32), AS (HR: 1.34; 95% CI: 1.29-1.38), AR (HR: 1.24; 95% CI: 1.18-1.31), and MR (HR: 1.27, 95% CI: 1.23-1.31). Compared with the first quartile group (Q1) for KDM-BA acceleration, participants in Q4 had a higher risk of VHD (HR: 1.86; 95% CI: 1.74-1.99), AS (HR: 2.68; 95% CI: 2.40-3.00), AR (HR: 1.70; 95% CI: 1.43-2.01) and MR (HR: 1.48; 95% CI: 1.35-1.63). The associations were also significant for participants in Q4 of PhenoAge acceleration compared with Q1: VHD (HR: 1.67; 95% CI: 1.57-1.79), AS (HR: 1.92; 95% CI: 1.73-2.13), AR (HR: 1.68; 95% CI: 1.38-1.92), and MR (HR: 1.59; 95% CI: 1.45-1.74). Furthermore, the population attributable fraction analyses revealed that reducing KDM-BA acceleration or PhenoAge acceleration from Q4 to Q1 in all individuals could result in a reduction of 34.41% and 32.73% in incident VHD cases, respectively (Table 2).

**Table 2:** Associations of the Biological Age Accelerations With the Risk of VHD

Biological Age	Cases/Person-Years	Incidence Rate (95% CI), per 1,000 Person-Years	HR (95% CI)	*P* Value	PAF (95% CI), %
KDM-BA acceleration, continuous	8,146/4,512,447.4	1.81 (1.77-1.84)	1.35 (1.32-1.38)	<0.001	
KDM-BA acceleration, quartiles					
Q1	1,445/1,142,018.4	1.27 (1.20-1.33)	Reference		
Q2	1,702/1,136,599.6	1.50 (1.43-1.57)	1.13 (1.05-1.21)	<0.001	8.17 (4.84-11.49)
Q3	2,038/1,129,286.9	1.80 (1.73-1.88)	1.31 (1.22-1.40)	<0.001	17.03 (13.80-20.41)
Q4	2,961/1,104,542.5	2.68 (2.59-2.78)	1.86 (1.74-1.99)	<0.001	34.41 (31.78-37.04)
PhenoAge acceleration, continuous	8,146/4,512,447.4	1.81 (1.77-1.84)	1.29 (1.26-1.32)	<0.001	
PhenoAge acceleration, quartiles					
Q1	1,524/1,146,118.2	1.33 (1.26-1.40)	Reference		
Q2	1,683/1,141,056.3	1.47 (1.41-1.55)	1.06 (0.99-1.14)	0.090	4.96 (1.79-8.43)
Q3	1,932/1,131,566.5	1.71 (1.63-1.79)	1.15 (1.08-1.24)	<0.001	11.81 (8,46-15.25)
Q4	3,007/1,093,706.4	2.75 (2.65-2.85)	1.67 (1.57-1.79)	<0.001	32.73 (30.91-35.58)

Model adjusted for age, sex, ethnicity, education level, employment status, BMI, smoking status, drinking status, physical activity, and Townsend deprivation index.

PAF = population attributable fraction; Q = quartile; other abbreviations as in Table 1.

**Figure 1:**
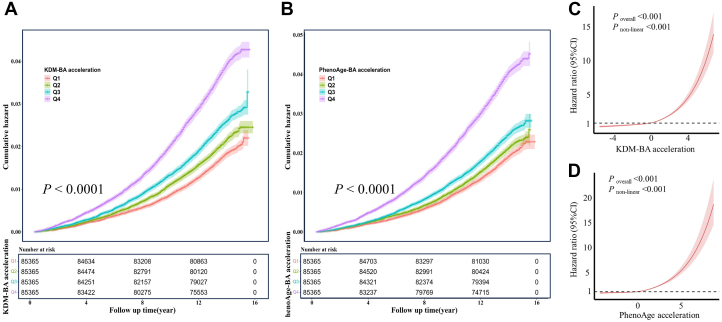
Association of Biological Aging With the Risk of VHD (A) Cumulative valvular heart disease (VHD) incidence difference among Klemera–Doubal method biological age (KDM-BA) acceleration groups. (B) Cumulative VHD incidence difference among PhenoAge acceleration groups. (C) Association between KDM-BA acceleration and the risk of VHD using restricted cubic spline regression. (D) Association between PhenoAge acceleration and the risk of VHD using restricted cubic spline regression. All models were adjusted for age, sex, ethnicity, education level, employment status, body mass index, smoking status, drinking status, physical activity, and Townsend deprivation index. Q = quartile.

In addition, we plotted the cumulative probability of VHD between the 4 groups of KDM-BA acceleration and PhenoAge acceleration, and tested for differences using the log-rank test (both *P* < 0.0001) (Figures 1A and 1B). Restricted cubic spline analyses showed that the association between KDM-BA acceleration/PhenoAge acceleration and the incidence of VHD both exhibited a monotonically increasing, nonlinear trend (*P*_overall_ < 0.001, *P*_non-linear_ < 0.001) (Figures 1C and 1D). The same trends were also observed in individuals with AS, AR, or MR (Supplemental Figures 3 to 5).

**Figure 2:**
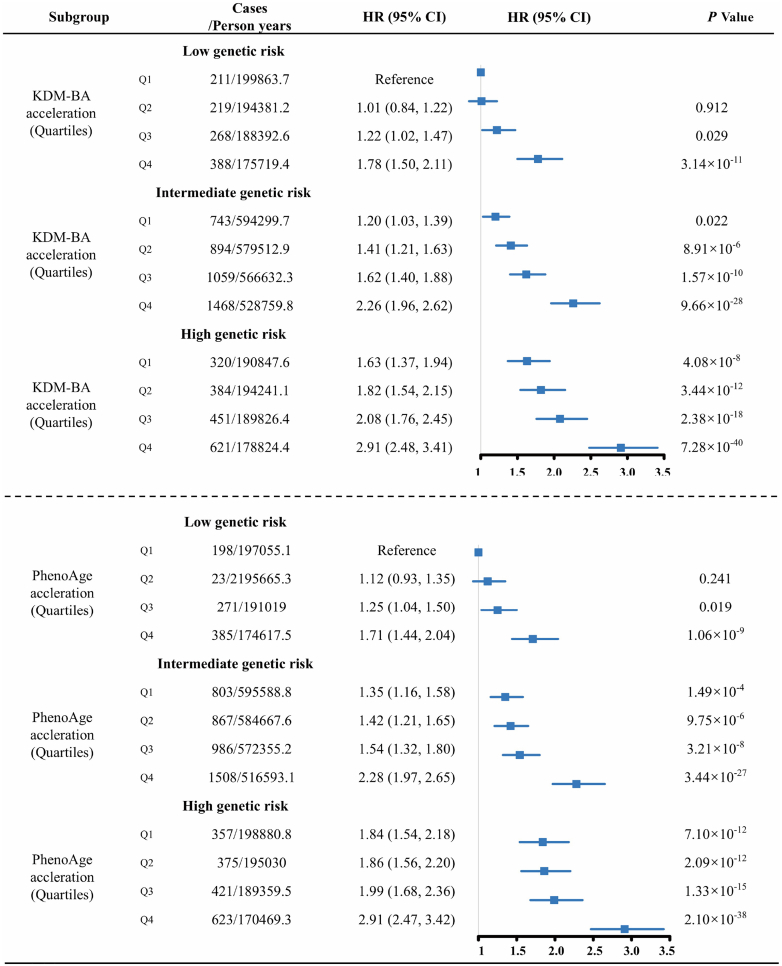
Joint Effects of Biological Aging and PRS on VHD risk Model adjusted for age, sex, education level, employment status, body mass index, smoking status, drinking status, physical activity, and Townsend deprivation index. PRS = polygenic risk score; other abbreviations as in Figure 1.

The PRS distribution for individuals was normal (Supplemental Figure 6). The VHD-PRS groups exhibited a positive relationship with the risk of VHD (Supplemental Table 13). We did not observe significant modifying effects of genetic risk on the association between biological age accelerations and the risk of VHD (*P*_interaction_ = 0.867 for KDM-BA acceleration; *P*_interaction_ = 0.921 for PhenoAge acceleration), AR (*P*_interaction_ = 0.275 for KDM-BA acceleration; *P*_interaction_ = 0.698 for PhenoAge acceleration), and MR (*P*_interaction_ = 0.615 for KDM-BA acceleration; *P*_interaction_ = 0.349 for PhenoAge acceleration). For AS, genetic risk exhibited a mixed modifying effect (*P*_interaction_ = 0.032 for KDM-BA acceleration; *P*_interaction_ = 0.304 for PhenoAge acceleration). Joint associations showed an increasing trend in HRs for VHD risk following higher KDM-BA acceleration and PhenoAge acceleration across all low, intermediate, and high genetic risk groups (Figure 2). The highest risk of VHD occurred in participants with the highest KDM-BA acceleration and high genetic risk (HR: 2.91; 95% CI: 2.48-3.41), as well as in participants with the highest PhenoAge acceleration and high genetic risk (HR: 2.91; 95% CI: 2.47-3.42). Similar results were observed in the joint association analyses for AS, AR, and MR (Supplemental Figures 7 to 9). In addition, we explored the association between biological aging and outcomes across different genetic risk groups. The results showed that older biological age was associated with an increased risk of VHD, AS, AR, and MR in all genetic risk groups (Supplemental Table 14 to 17, Supplemental Figures 10 to 13).

A significant additive interaction between biological age and PRS for VHD was present in Table 3. Specifically, for participants with both high PRS and who were in Q4 of KDM-BA acceleration, the RERI was 0.50 (95% CI: 0.16-0.85), indicating a 0.50-fold excess risk attributable to the interaction between genetic predisposition and accelerated biological aging, accounting for 17% (95% CI: 6%-29%) of the total VHD risk in individuals exposed to both high PRS and the oldest biological age (Q4). Similarly, for individuals with high PRS and in Q4 of PhenoAge acceleration, the RERI was 0.36 (95% CI: 0.00-0.71), accounting for 12% (95% CI: 0%-25%) of the total risk. The additive interaction observed in AS was similar to that in VHD, whereas no additive interaction was found in AR or MR (Supplemental Tables 18 to 20).

**Table 3:** Additive Interaction Between Biological Age Acceleration and Genetic Risk Categories on Incident VHD

		Intermediate Genetic Risk		High Genetic Risk	
		RERI (95% CI)	AP (95% CI)	RERI (95% CI)	AP (95% CI)
KDM-BA acceleration	Q2	0.20 (−0.01 to 0.41)	0.14 (−0.02 to 0.30)	0.18 (−0.13 to 0.49)	0.10 (−0.07 to 0.27)
	Q3	0.20 (−0.02 to 0.43)	0.13 (−0.02 to 0.27)	0.23 (−0.09 to 0.55)	0.11 (−0.04 to 0.26)
	Q4	0.29 (0.04-0.54)	0.13 (0.01-0.24)	0.50 (0.16-0.85)	0.17 (0.06-0.29)
PhenoAge acceleration	Q2	−0.05 (−0.30 to 0.19)	−0.04 (−0.21 to 0.13)	−0.10 (−0.44 to 0.24)	−0.05 (−0.24 to 0.13)
	Q3	−0.06 (−0.31 to 0.19)	−0.04 (−0.20 to 0.12)	−0.09 (−0.43 to 0.25)	−0.04 (−0.22 to 0.13)
	Q4	0.22 (−0.03 to 0.47)	0.10 (−0.02 to 0.21)	0.36 (0.00-0.71)	0.12 (0.00-0.25)

The relative excess risk of interaction (RERI) and attributable proportion (AP) were calculated by the “Delta” method.

Abbreviations as in Tables 1 and 2.

In stratified analyses (Supplemental Table 21), the associations between biological ages and VHD did not vary by ethnicity, BMI, employment status, smoking status, and drinking status, except for sex, age, and physical activity. Specifically, stronger associations of biological ages (both KDM-BA and PhenoAge acceleration) with VHD were observed among male, younger adults (<65 years), and individuals with low physical activity.

In the sensitivity analyses, the results yielded consistent results with the main findings after excluding participants with missing covariates (Supplemental Tables 22 to 25, Supplemental Figure 14), excluding those who developed VHD within the first 2 years of follow-up (Supplemental Tables 26 to 29, Supplemental Figure 15), or using a broader definition of VHD as the outcome (Supplemental Tables 30 to 33, Supplemental Figure 16). Furthermore, after additional adjustment for comorbidities, including hypertension, stroke, atrial fibrillation, chronic kidney disease, cancer, and type 2 diabetes, the results remained significant, though the effect size were attenuated (Supplemental Tables 34 to 37, Supplemental Figure 17). Participants were stratified by biological age status (younger vs older than chronological age). Biological age acceleration was significantly associated with increased VHD risk. Compared with biologically younger individuals, those classified as biologically older exhibited substantially higher VHD incidence (HR: 1.46; 95% CI: 1.39-1.53 for KDM-BA acceleration; HR: 1.39; 95% CI: 1.33-1.45 for PhenoAge acceleration), as shown in Supplemental Table 38 and Supplemental Figure 18. The joint association and additive interactions remained consistent with the main analyses (Supplemental Tables 39 to 41).

## Discussion

In the present study, we evaluated the impact of KDM-BA acceleration and PhenoAge acceleration, two indicators of biological aging, on the incidence of VHD. Our main results revealed that participants with older biological ages had a higher risk of VHD in comparison to those who were biologically younger. Moreover, biological-aging-associated risk of VHD was independent of and additive to genetic risk measured by PRS (Central Illustration). To the best of our knowledge, this is the first study to support the hypothesis that biological aging might be a risk factor of VHD, providing novel insights into the possibility that targeting biological aging could contribute to VHD prevention.

**Central Illustration:**
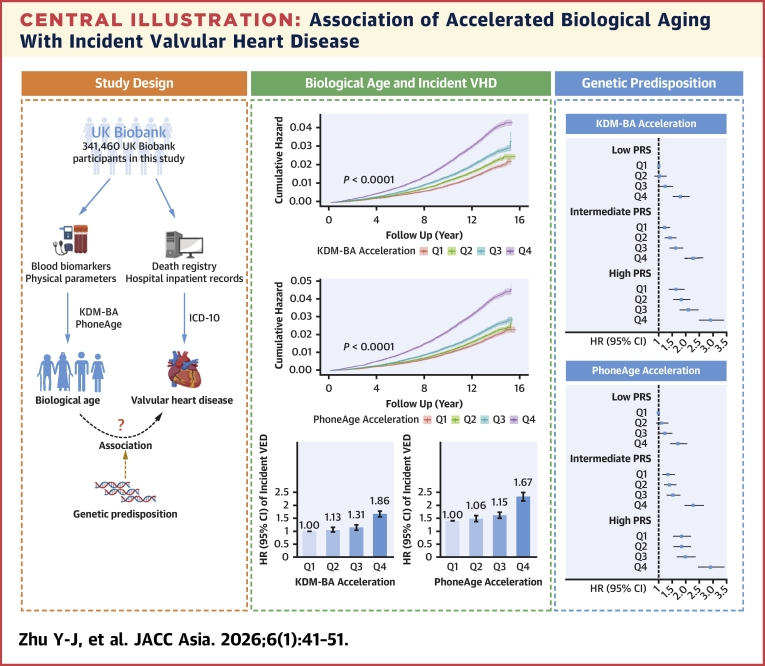
Association of Accelerated Biological Aging With Incident Valvular Heart Disease The multicenter population-based cohort included 341,460 participants from the UK Biobank to assess the relationships between biological aging with incident valvular heart disease (VHD). After adjusting for sociodemographic features, advanced biological aging was significantly associated with an increased risk of incident VHD. No significant interaction between biological aging and genetic risk with the risk of valvular heart disease. Biological-aging-associated risk of VHD was independent of and additive to genetic risk measured by PRS. ICD-10 = International Classification of Diseases coding system 10; KDM-BA = Klemera-Doubal method biological age; PRS = polygenic risk score; Q = quartile. This figure was partly created with biorender.com.

The extant literature suggests that biological age is a critical risk factor for cardiovascular diseases.^7^^,^^8^^,^^10^^,^^16^^,^^24^, ^25^, ^26^, ^27^, ^28^ However, most studies in this field have primarily focused on single biomarkers of biological aging (ie, telomere length and DNA methylation age) to explore their association with CVD. For example, an study based on the Hong Kong Diabetes Register found shortened relative leukocyte telomere length may elevate the risk cardiovascular complications in type 2 diabetes.^8^ A cohort study from the PIVUS study (Prospective Study of the Vasculature in Uppsala Seniors) observed that increased biological age, as estimated by DNA methylation, is linked to an increased risk of CVD.^7^ Although these individual biomarkers provide valuable insights, constructing a composite, clinically accessible, and validated biomarker is crucial. To date, studies specifically exploring the association between composite biomarkers of biological age and CVD are limited, with only 2 cohort studies investigating the link of biological aging calculated by KDM-BA and the PhenoAge method with outcomes such as heart failure and cardiometabolic multimorbidity.^10^^,^^16^ Studies remain scarce on the relationship between comprehensive biological age markers and VHD. This study fills that gap and demonstrates that advanced biological aging increases the risk of VHD.

Our results contribute to establishing a prospective relationship between accelerated biological aging and the risk of VHD, although the potential mechanisms underlying this association were not addressed in the study. Two recent reviews have summarized the characteristics of aging and the mechanisms underlying CVD, which offer important clues into the overlapping mechanisms between aging and VHD.^5^^,^^6^ First, several studies support the role for chronic inflammation, also known as inflammageing.^29^, ^30^, ^31^, ^32^, ^33^ The older organisms tend to develop a proinflammatory status, which increases their predisposition to chronic disease, including CVD.^30^, ^31^, ^32^, ^33^ Meanwhile, inflammageing is a recognized risk factor for CVD, and clinical trials have suggest that this association is causal.^34^, ^35^, ^36^, ^37^, ^38^ Second, mitochondrial dysfunction is a common denominator in both biological aging and CVD pathology. An animal study observed a notable decline in Nrf2 activity and mitochondrial function, as well as cardiac dysfunction, in older control mice compared with the younger age group.^39^ Another animal study revealed mitochondrial dysfunction leads to telomere dysfunction and then contributes to the development of CVD.^40^ Moreover, other key processes involved in aging, such as deregulated nutrient sensing, loss of proteostasis, and cellular senescence, also play a significant role in the pathogenesis of CVD.^5^

Disparities in aging trajectories result from complex interactions between lifestyle factors and genetic predisposition. Evidence indicates that unhealthy behaviors increase cardiovascular disease, cancer, and mortality risk, potentially through accelerated biological aging processes.^24^ Our analysis demonstrated significant additive and synergistic effects between biological aging markers and PRS in predicting incident VHD. These findings highlight the multifactorial nature of VHD pathogenesis, involving dynamic gene-environment interactions. Importantly, individuals with elevated genetic risk may benefit from close monitoring of biological aging indicators.

Our findings suggest that the biological age measured by KDM-BA and PhenoAge method may serve as a valuable predictive biomarker for VHD to identify the high-risk populations with developed VHD, which is essential in the management of VHD in public health. Furthermore, emerging clinical trials aimed at slowing or reversing biological age have demonstrated promising preliminary outcomes, with the interventions physical activity, a healthy diet, and lifestyle changes proving to be effective in slowing aging.^41^, ^42^, ^43^ Further research is required to explore whether these interventions contribute to the prevention of VHD by slowing, halting, or even reversing the aging process.

### Study Strengths and Limitations

This study has 2 main strengths. First, the largescale, prospective study design of the UK Biobank provided adequate statistical power to obtain stable and precise estimates. Second, biological aging measured by multiple clinical biomarkers is more comprehensive and accessible, offering greater potential for widespread clinical application compared with other biological age indicators.

Despite these strengths, several limitations should be considered. First, all results of the main analyses and sensitivity analyses in our study support the hypothesis that advanced biological aging is positively associated with incident VHD, but UK Biobank is a volunteer cohort with participants likely to be healthier than the general population and has a selection bias. Second, we were unable to obtain the biochemical indicators used to construct biological age during follow-up, making it difficult to determine the changes in biological aging and corresponding associations with VHD. The data in the UK Biobank dataset are continuously accumulating, which provides the opportunity for further research focusing on changes in biological aging and health-related outcomes, including VHD. Third, it is challenging to generalize the results to a general population or other ethnic groups, as participants in the present study were mostly White. Finally, the present study is an observational study, which limits causal inference.

## Conclusions

The current study indicated that accelerated biological aging is associated with an increased risk of incident VHD and suggested a significant additive interaction between biological age and PRS for VHD. Thus, slowing biological aging and integrating it into VHD management could be targeted to reduce the cardiovascular disorders burden. Future research is required to investigate these implications in diverse populations.

### Data sharing statement

This research has been conducted using the public UK Biobank resource.

## Funding Support and Author Disclosures

This work was supported by the Open Research Fund of the National Health Commission Key Laboratory of Birth Defects Prevention and the Henan Key Laboratory of Population Defects Prevention (ZD202303), the 2022 International Postdoctoral Exchange Fellowship Program [Talent-Introduction Program] (No. YJ20220181), Henan Medical Science and Technology Research Program (No.232102310069). The authors have reported that they have no relationships relevant to the contents of this paper to disclose.

## References

[1] Coffey S., Roberts-Thomson R., Brown A. (2021). Global epidemiology of valvular heart disease. Nat Rev Cardiol.

[2] Li Z., Cheng S., Guo B. (2025). Wearable device-measured moderate to vigorous physical activity and risk of degenerative aortic valve stenosis. Eur Heart J.

[3] Iung B., Delgado V., Rosenhek R. (2019). Contemporary presentation and management of valvular heart disease: the EURObservational Research Programme Valvular Heart Disease II Survey. Circulation.

[4] Ajmone Marsan N., Delgado V., Shah D.J. (2023). Valvular heart disease: shifting the focus to the myocardium. Eur Heart J.

[5] Ungvari Z., Tarantini S., Sorond F., Merkely B., Csiszar A. (2020). Mechanisms of vascular aging, a geroscience perspective: JACC focus seminar. J Am Coll Cardiol.

[6] López-Otín C., Blasco M.A., Partridge L., Serrano M., Kroemer G. (2023). Hallmarks of aging: an expanding universe. Cell.

[7] Lind L., Ingelsson E., Sundström J., Siegbahn A., Lampa E. (2018). Methylation-based estimated biological age and cardiovascular disease. Eur J Clin Invest.

[8] Cheng F., Luk A.O., Tam C.H.T. (2020). Shortened relative leukocyte telomere length is associated with prevalent and incident cardiovascular complications in type 2 diabetes: analysis from the Hong Kong Diabetes Register. Diabetes Care.

[9] Gao X., Geng T., Jiang M. (2023). Accelerated biological aging and risk of depression and anxiety: evidence from 424,299 UK Biobank participants. Nat Commun.

[10] Mao R., Wang F., Zhong Y., Meng X., Zhang T., Li J. (2024). Association of biological age acceleration with cardiac morphology, function, and incident heart failure: insights from UK Biobank participants. Eur Heart J Cardiovasc Imaging.

[11] Wang T., Duan W., Jia X. (2024). Associations of combined phenotypic ageing and genetic risk with incidence of chronic respiratory diseases in the UK Biobank: a prospective cohort study. Eur Respir J.

[12] Klemera P., Doubal S. (2006). A new approach to the concept and computation of biological age. Mech Ageing Dev.

[13] Kothari M., Belsky D.W. (2021). Unite to predict. Elife.

[14] Liu Z., Kuo P.L., Horvath S., Crimmins E., Ferrucci L., Levine M. (2018). A new aging measure captures morbidity and mortality risk across diverse subpopulations from NHANES IV: a cohort study. PLoS Med.

[15] Graf G.H., Crowe C.L., Kothari M. (2022). Testing black-white disparities in biological aging among older adults in the United States: analysis of DNA-methylation and blood-chemistry methods. Am J Epidemiol.

[16] Jiang M., Tian S., Liu S. (2024). Accelerated biological aging elevates the risk of cardiometabolic multimorbidity and mortality. Nat Cardiovasc Res.

[17] Arsenault B.J., Kamstrup P.R. (2022). Lipoprotein(a) and cardiovascular and valvular diseases: a genetic epidemiological perspective. Atherosclerosis.

[18] Chou H.T., Chen C.H., Tsai C.H., Tsai F.J. (2004). Association between transforming growth factor-beta1 gene C-509T and T869C polymorphisms and rheumatic heart disease. Am Heart J.

[19] UK Biobank (2007). UK Biobank Protocol: A Large-Scale Prospective Epidemiological Resource. https://www.ukbiobank.ac.uk/wp-content/uploads/2025/01/Main-study-protocol.pdf.

[20] Kwon D., Belsky D.W. (2021). A toolkit for quantification of biological age from blood chemistry and organ function test data: BioAge. Geroscience.

[21] Yang C., Li Z., Sui Y. (2025). Sex differences in cardiovascular-kidney-metabolic risk factors associated with degenerative valvular heart disease. Eur J Prev Cardiol.

[22] Thériault S., Li Z., Abner E. (2024). Integrative genomic analyses identify candidate causal genes for calcific aortic valve stenosis involving tissue-specific regulation. Nat Commun.

[23] Jia C., Zeng Y., Huang X. (2023). Lifestyle patterns, genetic susceptibility, and risk of valvular heart disease:a prospective cohort study based on the UK Biobank. Eur J Prev Cardiol.

[24] Li X., Cao X., Zhang J. (2024). Accelerated aging mediates the associations of unhealthy lifestyles with cardiovascular disease, cancer, and mortality. J Am Geriatr Soc.

[25] Tyrrell D.J., Goldstein D.R. (2021). Ageing and atherosclerosis:vascular intrinsic and extrinsic factors and potential role of IL-6. Nat Rev Cardiol.

[26] Liberale L., Badimon L., Montecucco F., Lüscher T.F., Libby P., Camici G.G. (2022). Inflammation, aging, and cardiovascular disease: JACC review topic of the week. J Am Coll Cardiol.

[27] Haycock P.C., Heydon E.E., Kaptoge S., Butterworth A.S., Thompson A., Willeit P. (2014). Leucocyte telomere length and risk of cardiovascular disease:systematic review and meta-analysis. BMJ.

[28] Topriceanu C.C., Dev E., Ahmad M. (2023). Accelerated DNA methylation age plays a role in the impact of cardiovascular risk factors on the human heart. Clin Epigenetics.

[29] Ferrucci L., Fabbri E. (2018). Inflammageing: chronic inflammation in ageing, cardiovascular disease, and frailty. Nat Rev Cardiol.

[30] Kumar P., Liu C., Suliburk J. (2023). Supplementing glycine and N-acetylcysteine (GlyNAC) in older adults improves glutathione deficiency, oxidative stress, mitochondrial dysfunction, inflammation, physical function, and aging hallmarks: a randomized clinical trial. J Gerontol A Biol Sci Med Sci.

[31] Zhu H., Chen J., Liu K. (2023). Human PBMC scRNA-seq-based aging clocks reveal ribosome to inflammation balance as a single-cell aging hallmark and super longevity. Sci Adv.

[32] Zhao Y., Simon M., Seluanov A., Gorbunova V. (2023). DNA damage and repair in age-related inflammation. Nat Rev Immunol.

[33] Polizio A.H., Park E., Walsh K. (2023). Clonal hematopoiesis:connecting aging and inflammation in atherosclerosis. Curr Atheroscler Rep.

[34] Saaoud F., Liu L., Xu K. (2023). Aorta- and liver-generated TMAO enhances trained immunity for increased inflammation via ER stress/mitochondrial ROS/glycolysis pathways. JCI Insight.

[35] Ferrell M., Wang Z., Anderson J.T. (2024). A terminal metabolite of niacin promotes vascular inflammation and contributes to cardiovascular disease risk. Nat Med.

[36] Valenzuela P.L., Ruilope L.M., Santos-Lozano A. (2023). Exercise benefits in cardiovascular diseases:from mechanisms to clinical implementation. Eur Heart J.

[37] Nyárády B.B., Kiss L.Z., Bagyura Z. (2024). Growth and differentiation factor-15: a link between inflammaging and cardiovascular disease. Biomed Pharmacother.

[38] Toba H., Lindsey M.L. (2019). Extracellular matrix roles in cardiorenal fibrosis:Potential therapeutic targets for CVD and CKD in the elderly. Pharmacol Ther.

[39] Bose C., Alves I., Singh P. (2020). Sulforaphane prevents age-associated cardiac and muscular dysfunction through Nrf2 signaling. Aging Cell.

[40] Gordon C.A., Madamanchi N.R., Runge M.S., Jarstfer M.B. (2022). Effect of oxidative stress on telomere maintenance in aortic smooth muscle cells. Biochim Biophys Acta Mol Basis Dis.

[41] Yang K., Hou R., Zhao J. (2023). Lifestyle effects on aging and CVD: a spotlight on the nutrient-sensing network. Ageing Res Rev.

[42] Thomas A., Ryan C.P., Caspi A. (2024). Diet, pace of biological aging, and risk of dementia in the Framingham Heart Study. Ann Neurol.

[43] Fiorito G., Caini S., Palli D. (2021). DNA methylation-based biomarkers of aging were slowed down in a two-year diet and physical activity intervention trial: the DAMA study. Aging Cell.

