# Supplementary material for: Accelerated Biological Aging, Genetic Predisposition, and Incident Valvular Heart Disease
Source: JACC Asia. 2025 Aug 16;6(1):41–51. doi: 10.1016/j.jacasi.2025.06.011 (PMC12833606; doi:10.1016/j.jacasi.2025.06.011)
Supplement: Supplemental Material [file mmc1.docx]

**Supplemental Materials**

**Supplemental Method** Algorithms of Biological age

**Supplemental Tables**

**Supplemental Table 1** Data field IDs of variables for the construction of biological age

**Supplemental Table 2** Diagnostic codes for diseases in the study

**Supplemental Table 3 Logistic Regression Analysis of Missingness by Predictor Variables**

**Supplemental Table 4** Baseline characteristics of participants by AS status

**Supplemental Table 5** Baseline characteristics of participants by AR status

**Supplemental Table 6** Baseline characteristics of participants by MR status

**Supplemental Table 7** Baseline characteristics of participants by KDM-BA acceleration (Quartiles)

**Supplemental Table 8** Baseline characteristics of participants by PhenoAge acceleration (Quartiles)

**Supplemental Table 9** Characteristics of Components of biological age by VHD status

**Supplemental Table 10** Associations of the biological age accelerations with the risk of AS

**Supplemental Table 11** Associations of the biological age accelerations with the risk of AR

**Supplemental Table 12** Associations of the biological age accelerations with the risk of MR

**Supplemental Table 13** Association between genetic predisposition and incident VHD

**Supplemental Table 14** Associations of biological age (Continuous) with incident VHD within each genetic risk category

**Supplemental Table 15** Associations of biological age (Continuous) with incident AS within each genetic risk category

**Supplemental Table 16** Associations of biological age (Continuous) with incident AR within each genetic risk category

**Supplemental Table 17** Associations of biological age (Continuous) with incident MR within each genetic risk category

**Supplemental Table 18** Additive interaction between biological age acceleration and genetic risk categories on incident AS

**Supplemental Table 19** Additive interaction between biological age acceleration and genetic risk categories on incident AR

**Supplemental Table 20** Additive interaction between biological age acceleration and genetic risk categories on incident MR

**Supplemental Table 21** Stratified analyses for associations between biological age accelerations and the risk of VHD

**Supplemental Table 22** Associations of the biological age accelerations with the risk of VHD among individuals without missing covariate data

**Supplemental Table 23** Joint effects of biological age accelerations and PRS on the risk of VHD among individuals without missing covariate data

**Supplemental Table 24** Associations of biological age accelerations with incident VHD in different genetic risk backgrounds among individuals without missing covariate data

**Supplemental Table 25** Additive interaction between biological age acceleration and genetic risk categories on incident VHD among individuals without missing covariate data

**Supplemental Table 26**Associations of biological ages with incident VHD after excluding participants with VHD in the first 2 years

**Supplemental Table 27** Joint effects of biological age accelerations and PRS on the risk of VHD after excluding participants with VHD in the first 2 years

**Supplemental Table 28** Associations of biological age accelerations with incident VHD in different genetic risk backgrounds after excluding participants with VHD in the first 2 years

**Supplemental Table 29** Additive interaction between biological age acceleration and genetic risk categories on incident VHD after excluding participants with VHD in the first 2 years

**Supplemental Table 30** Associations of biological ages with incident VHD diagnosed by a broad definition.

**Supplemental Table 31** Joint effects of biological age accelerations and PRS on the risk of VHD diagnosed by a broad definition.

**Supplemental Table 32** Associations of biological age accelerations with incident VHD diagnosed by a broad definition in different genetic risk backgrounds

**Supplemental Table 33** Additive interaction between biological age acceleration and genetic risk categories on incident VHD diagnosed by a broad definition

**Supplemental Table 34** Associations of biological ages with incident VHD by additionally controlling for comorbidities

**Supplemental Table 35** Joint effects of biological age accelerations and PRS on the risk of VHD by additionally controlling for comorbidities

**Supplemental Table 36** Associations of biological age accelerations with incident VHD in different genetic risk backgrounds by additionally controlling for comorbidities

**Supplemental Table 37** Additive interaction between biological age acceleration and genetic risk categories on incident VHD by additionally controlling for comorbidities

**Supplemental Table 38** Associations of biological ages with incident VHD in biologically younger and older groups

**Supplemental Table 39** Joint effects of biological age accelerations and PRS on the risk of VHD in biologically younger and older groups

**Supplemental Table 40** Associations of biological age accelerations with incident VHD in different genetic risk backgrounds in biologically younger and older groups

**Supplemental Table 41** Additive interaction between biological age acceleration and genetic risk categories on incident VHD in biologically younger and older groups

**Supplemental Figures**

**Supplemental Figure 1** Flowchart of participant selection

**Supplemental Figure 2 UpSet plot showing the missing data patterns across 7 variables in the dataset**

**Supplemental Figure 3** Association of biological aging with the risk AS

**Supplemental Figure 4** Association of biological aging with the risk AR

**Supplemental Figure 5** Association of biological aging with the risk MR

**Supplemental Figure 6** The Curve of density distribution of polygenic risk score

**Supplemental Figure 7** Joint effects of biological age accelerations and PRS on the risk of AS

**Supplemental Figure 8** Joint effects of biological age accelerations and PRS on the risk of AR

**Supplemental Figure 9** Joint effects of biological age accelerations and PRS on the risk of MR

**Supplemental Figure 10** Associations of biological age accelerations with incident VHD in different genetic risk backgrounds

**Supplemental Figure 11** Associations of biological age accelerations with incident AS in different genetic risk backgrounds

**Supplemental Figure 12** Associations of biological age accelerations with incident AR in different genetic risk backgrounds

**Supplemental Figure 13** Associations of biological age accelerations with incident MR in different genetic risk backgrounds

**Supplemental Figure 14** Association of biological aging with the risk VHD among individuals without missing covariate data

**Supplemental Figure 15** Association of biological aging with the risk VHD after excluding participants with VHD in the first 2 years.

**Supplemental Figure 16** Association of biological aging with the risk VHD diagnosed by a broad definition

**Supplemental Figure 17** Association of biological aging with the risk VHD by additionally controlling for comorbidities

**Supplemental Figure 18** Association of biological aging with the risk VHD in biologically younger and older groups

**Supplemental Method**

**Algorithms of Biological age**

All calculation of biological age were computed using the R package “BioAge” (<https://github.com/dayoonkwon/BioAge>)^1^

1. Klemera-Doubal method Biological Age (KDM-BA)

An individual’s KDM-BA prediction corresponds to the chronological age at which her/his physiology would be approximately normal. The KDM-BA is derived from a series of regressions of individual biomarkers on chronological age in a reference population. The equation takes information from *n* number of regression lines of chronological age regressed on *n* biomarkers. The formula is:

$$KDM-BA_{EC}= \frac{\Sigma_{ⅈ=1}^{n}\left( x_{i}-q_{i} \right)\frac{k_{i}}{s_{i}^{2}}+\frac{CA}{S_{BA}^{2}}}{\Sigma_{ⅈ=1}^{n}\left( \frac{k_{i}}{s_{i}} \right)^{2}+\frac{1}{S_{BA}^{2}}}$$

Where *x* is the value of biomarker *i* measured for an individual. For each biomarker *i*, the parameters *k*, *q*, and *s* are estimated from a regression of chronological age on the biomarker in the reference sample. *k*, *q*, and *s* are the regression intercept, slope, and root mean squared error, respectively. *s*_BA_ is a scaling factor equal to the square root of the variance in chronological age explained by the biomarker set in the reference sample. CA is chronological age. In the kdm_nhanes function in BioAge package, the reference sample is NHANES III nonpregnant participants aged 30–75 years. Algorithm parameters are estimated separately for men and women. We collected nine biomarkers including forced expiratory volume in one second (FEV_1_), systolic blood pressure, albumin, alkaline phosphatase, blood urea nitrogen, creatinine, C-reactive protein, glycated hemoglobin, and total cholesterol for calculation of biological age.

1. PhenoAge algorithm

The PhenoAge algorithm is derived from multivariate analysis of mortality hazards. The original PhenoAge algorithm was constructed from elastic-net Gompertz regression of mortality on 42 biomarkers in the NHANES III. This analysis selected nine biomarkers: albumin, alkaline phosphatase, creatinine, C-reactive protein, glucose, mean cell volume, red cell distribution width, white blood cell count, and lymphocyte proportion, and chronological age. The formula is:

$$\mathrm{PhenoAge}=141.5-225+ \frac{ln[-0.00553\times\ln(1-mortality risk)}{0.090165}$$

where

$$mortality risk=1-ⅇ^{-ⅇ^{xb}\left[ \exp\left( 12o_{x\gamma} \right)-1 \right]/\gamma}$$

$$\gamma=0.0076927$$

$$xb =-19.907-0.0336+albumin+0.0095\times creatinine+0.1953\times glucose+0.0954\times ln(C-reactive protein)-0.012\times lymphocyte precentage+0.0268\times mean corpuscular volume+0.3306\times red cell distribution width+0.00188\times alkaline phophatase+0.0554\times white blood cell count+0.0804\times chronological age$$

**Reference**

1. Kwon D, Belsky DW. A toolkit for quantification of biological age from blood chemistry and organ function test data: BioAge. *Geroscience* 2021; 43(6): 2795-808

**Supplemental Table 1 Data field IDs of variables for the construction of biological age**

| **Physical parameters and blood biomarker** | **Field ID in the UK Biobank** |
| --- | --- |
| Forced expiratory volume in one second, FEV1 (L) | 3063 |
| Systolic blood pressure (mm Hg) | 4080 |
| Total Cholesterol (mg/dL) | 30690 |
| Glycated hemoglobin (%) | 30750 |
| Blood urea nitrogen (mg/dL) | 30670 |
| Lymphocyte (%) | 30180 |
| Mean cell volume (fL) | 30270 |
| Serum glucose (mg/dL) | 30740 |
| Red cell distribution width (%) | 30070 |
| White blood cell count (1000 cells/uL) | 30000 |
| Albumin (g/dL) | 30600 |
| Creatinine (mg/dL) | 30700 |
| C-reactive protein (mg/dL) | 30710 |
| Alkaline phosphatase (U/L) | 30610 |

**Supplemental Table 2 Diagnostic codes for diseases in the study**

|  | International Classification of Diseases-10 (ICD-10) | |  |
| --- | --- | --- | --- |
| **Main Analysis (definition of VHD)** | | |  |
| aortic valve stenosis (AS) | I35.0、I35.2 | |  |
| aortic valve regurgitation (AR) | I35.1 | |  |
| mitral valve regurgitation (MR) | I34.0 | |  |
| **Congenital valvular heart disease (one of exclusion criteria)** | | | |
| Congenital malformations of pulmonary and tricuspid valves | | | Q22 |
| Congenital malformations of aortic and mitral valves | | | Q23 |
| **Sensitivity Analysis (broad definition of VHD ^1^)** | | |  |
| Rheumatic mitral valve diseases | I05 | |  |
| Rheumatic aortic valve diseases | I06 | |  |
| Rheumatic tricuspid valve diseases | I07 | |  |
| Multiple valve diseases | I08 | |  |
| Nonrheumatic mitral valve disorders | I34 | |  |
| Nonrheumatic aortic valve disorders | I35 | |  |
| Nonrheumatic tricuspid valve disorders | I36 | |  |
| Pulmonary valve disorders | I37 | |  |
| Valvular disorders in diseases classified elsewhere | I39.0、I39.1、 I39.3、I39.4 | |  |
| **Sensitivity Analysis (Comorbidities)** | | |  |
| Hypertension | I10, I11, I12, I13, I15, O10 | |  |
| Stroke | I60, I61, I62, I63, I64, I69 | |  |
| Atrial fibrillation | I48 | |  |
| Chronic kidney disease | N18 | |  |

References:

1. Jia C, Zeng Y, Huang X, Yang H, Qu Y, Hu Y, Chen W, Yang X. Lifestyle patterns, genetic susceptibility, and risk of valvular heart disease: a prospective cohort study based on the UK Biobank. Eur J Prev Cardiol. 2023 Oct 26;30(15):1665-1673. doi: 10.1093/eurjpc/zwad177. PMID: 37259902.

**Supplemental Table 3 Logistic Regression Analysis of Missingness by Predictor Variables**

|  | **OR (95% CI)** | **P value** |  |  | **OR (95% CI)** | **P value** |
| --- | --- | --- | --- | --- | --- | --- |
| **Employment status** | | |  | **Physical activity level** | | |
| Age | 0.99 (0.99-1.00) | 0.018 |  | Age | 1.01 (1.01-1.01) | <0.001 |
| Sex | 0.93 (0.85-1.02) | 0.131 |  | Sex | 0.62 (0.61-0.63) | <0.001 |
| Ethnicity | 3.12 (2.70-3.58) | <0.001 |  | Ethnicity | 1.17 (1.13-1.22) | <0.001 |
| Education level | 0.63 (0.56-0.71) |  |  | Employment status | 1.01 (0.99-1.03) | 0.411 |
| Smoking status |  |  |  | Education level | 0.51 (0.50-0.52) | <0.001 |
| Ever | 1.02 (0.91-1.13) | 0.777 |  | Smoking status |  |  |
| Current | 1.05 (0.90-1.23) | 0.499 |  | Ever | 0.92 (0.90-0.94) | <0.001 |
| Drinking status |  |  |  | Current | 1.11 (1.07-1.14) | <0.001 |
| Ever | 0.68 (0.51-0.91) | 0.01 |  | Drinking status |  |  |
| Current | 0.65 (0.55-0.78) | <0.001 |  | Ever | 0.91 (0.86-0.97) | 0.003 |
| Physical activity level | |  |  | Current | 0.86 (0.82-0.90) | <0.001 |
| Moderate | 1.12 (0.98-1.28) | 0.103 |  | TDI | 1.02 (1.02-1.03) | <0.001 |
| High | 1.20 (1.05-1.37) | 0.009 |  | BMI | 1.02 (1.02-1.02) | <0.001 |
| TDI | 1.05 (1.04-1.07) | <0.001 |  | VHD | 0.97 (0.91-1.02) | 0.236 |
| BMI | 0.99 (0.98-1.00) | 0.044 |  |  |  |  |
| VHD | 1.04 (0.75-1.40) | 0.792 |  |  |  |  |
|  |  |  |  |  |  |  |
| **Education level** | | |  | **Townsend Deprivation Index** | | |
| Age | 1.04 (1.03-1.04) | <0.001 |  | Age | 0.96 (0.95-0.98) | <0.001 |
| Sex | 1.09 (0.98-1.20) | 0.105 |  | Sex | 1.15 (0.93-1.43) | 0.188 |
| Ethnicity | 3.90 (3.37-4.50) | <0.001 |  | Ethnicity | 1.20 (0.76-1.81) | 0.418 |
| Employment status | 0.55 (0.49-0.62) | <0.001 |  | Employment status | 1.04 (0.80-1.37) | 0.766 |
| Smoking status |  |  |  | Education level | 1.12 (0.79-1.62) | 0.534 |
| Ever | 0.99 (0.89-1.11) | 0.885 |  | Smoking status |  |  |
| Current | 1.23 (1.05-1.44) | 0.011 |  | Ever | 1.03 (0.81-1.30) | 0.817 |
| Drinking status |  |  |  | Current | 1.00 (0.69-1.40) | 0.981 |
| Ever | 0.69 (0.53-0.91) | 0.008 |  | Drinking status |  |  |
| Current | 0.55 (0.46-0.65) | <0.001 |  | Ever | 0.77 (0.34-1.64) | 0.498 |
| Physical activity level | |  |  | Current | 0.78 (0.48-1.35) | 0.337 |
| Moderate | 0.97 (0.84-1.12) | 0.638 |  | Physical activity level | |  |
| High | 1.32 (1.15-1.52) | <0.001 |  | Moderate | 0.86 (0.65-1.14) | 0.294 |
| TDI | 1.06 (1.05-1.08) | <0.001 |  | High | 0.83 (0.62-1.10) | 0.185 |
| BMI | 1.02 (1.01-1.03) | <0.001 |  | BMI | 1.02 (1.00-1.04) | 0.100 |
| VHD | 1.03 (0.77-1.35) | 0.833 |  | VHD | 1.56 (0.77-2.79) | 0.174 |
|  |  |  |  |  |  |  |
| **Smoking status** | | |  | **Body Mass Index (BMI)** | | |
| Age | 1.02 (1.01-1.33) | 0.002 |  | Age | 1.00 (0.99-1.02) | 0.686 |
| Sex | 0.97 (0.82-1.13) | 0.681 |  | Sex | 1.72 (1.42-2.10) | <0.001 |
| Ethnicity | 1.87 (1.36-2.53) | <0.001 |  | Ethnicity | 3.69 (2.81-4.79) | <0.001 |
| Employment status | 0.89 (0.74-1.08) | 0.248 |  | Employment status | 0.44 (0.35-0.56) | <0.001 |
| Education level | 0.38 (0.31-0.45) | <0.001 |  | Education level | 0.83 (0.66-1.06) | 0.138 |
| Drinking status |  |  |  | Smoking status |  |  |
| Ever | 1.05 (0.60-1.80) | 0.866 |  | Ever | 0.93 (0.74-1.15) | 0.484 |
| Current | 1.11 (0.77-1.66) | 0.584 |  | Current | 1.00 (0.74-1.34) | 0.986 |
| Physical activity level | |  |  | Drinking status |  |  |
| Moderate | 0.87 (0.70-1.08) | 0.197 |  | Ever | 1.65 (1.03-2.66) | 0.038 |
| High | 0.91 (0.73-1.13) | 0.368 |  | Current | 0.86 (0.60-1.27) | 0.445 |
| TDI | 1.03 (1.01-1.04) | 0.039 |  | Physical activity level | |  |
| BMI | 1.02 (1.01-1.04) | 0.003 |  | Moderate | 0.38 (0.31-0.48) | <0.001 |
| VHD | 1.22 (0.77-1.81) | 0.365 |  | High | 0.30 (0.24-0.38) | <0.001 |
|  |  |  |  | TDI | 1.10 (1.07-1.14) | <0.001 |
| **Drinking status** | | |  | VHD | 1.04 (0.58-1.71) | 0.897 |
| Age | 0.98 (0.95-1.00) | 0.037 |  |  |  |  |
| Sex | 0.97 (0.70-1.35) | 0.877 |  |  |  |  |
| Ethnicity | 4.66 (3.07-6.93) | <0.001 |  |  |  |  |
| Employment status | 0.54 (0.37-0.78) | 0.001 |  |  |  |  |
| Education level | 0.55 (0.37-0.82) | 0.003 |  |  |  |  |
| Smoking status |  |  |  |  |  |  |
| Ever | 1.00 (0.67-1.48) | 0.984 |  |  |  |  |
| Current | 2.15 (1.40-3.26) | 0.003 |  |  |  |  |
| Physical activity level | |  |  |  |  |  |
| Moderate | 0.85 (0.56-1.31) | 0.455 |  |  |  |  |
| High | 0.72 (0.47-1.12) | 0.145 |  |  |  |  |
| TDI | 1.12 (1.06-1.17) | <0.001 |  |  |  |  |
| BMI | 1.00 (0.97-1.04) | 0.805 |  |  |  |  |
| VHD | 0.55 (0.09-1.74) | 0.403 |  |  |  |  |

**Supplemental Table 4 Baseline characteristics of participants by AS status**

|  | Overall  (N= 341,460) | non-AS  (n= 337,981) | AS  (n= 3,479) |
| --- | --- | --- | --- |
| Age, mean (SD), y | 56.89 (8.09) | 56.82 (8.09) | 63.60 (5.31) |
| Female, n (%) | 184603 (54.1) | 183320 (54.2) | 1283 (36.9) |
| White, n (%) | 323205 (94.7) | 319815 (94.6) | 3390 (97.4) |
| **Income, n (%)** |  |  |  |
| <£18,000 | 81207 (23.8) | 79882 (23.6) | 1325 (38.1) |
| £18,000 to £30,999 | 87942 (25.8) | 86913 (25.7) | 1029 (29.6) |
| £31,000 to £51,999 | 87527 (25.6) | 86871 (25.7) | 656 (18.9) |
| £52,000 to £100,000 | 67167 (19.7) | 66789 (19.8) | 378 (10.9) |
| > £100,000 | 17617 (5.2) | 17526 (5.2) | 91 (2.6) |
| **Education level, n (%)** |  |  |  |
| College or University degree | 112295 (32.9) | 111488 (33.0) | 807 (23.2) |
| Others | 229165 (67.1) | 226493 (67.0) | 2672 (76.8) |
| **Employment status, n (%)** |  |  |  |
| No | 140246 (41.1) | 137853 (40.8) | 2393 (68.8) |
| Yes | 201214 (58.9) | 200128 (59.2) | 1086 (31.2) |
| **Physical activity, n (%)** |  |  |  |
| Low | 63324 (18.5) | 62554 (18.5) | 770 (22.1) |
| Moderate | 139163 (40.8) | 137799 (40.8) | 1364 (39.2) |
| High | 138973 (40.7) | 137628 (40.7) | 1345 (38.7) |
| **Smoking status, n (%)** |  |  |  |
| Never | 188775 (55.3) | 187353 (55.4) | 1422 (40.9) |
| Ever | 117640 (34.5) | 116007 (34.3) | 1633 (46.9) |
| Current | 35045 (10.3) | 34621 (10.2) | 424 (12.2) |
| **Drinking status, n (%)** |  |  |  |
| Never | 14664 (4.3) | 14504 (4.3) | 160 (4.6) |
| Ever | 11614 (3.4) | 11435 (3.4) | 179 (5.1) |
| Current | 315182 (92.3) | 312042 (92.3) | 3140 (90.3) |
| BMI, kg/m^2^, mean (SD) | 27.34 (4.69) | 27.31 (4.68) | 29.60 (5.36) |
| Townsend deprivation index, mean (SD) | -1.37 (3.05) | -1.37 (3.05) | -1.26 (3.17) |
| **Biological ages, mean (SD)** |  |  |  |
| KDM-BA | 54.29 (9.30) | 54.20 (9.28) | 63.37 (7.17) |
| KDM-BA acceleration | -0.01 (4.83) | -0.04 (4.82) | 2.50 (5.63) |
| PhenoAge | 47.92 (10.00) | 47.82 (9.97) | 57.39 (8.26) |
| PhenoAge acceleration | -0.01 (5.36) | -0.03 (5.34) | 2.32 (6.29) |

Quantitative variables were presented as mean with SD and categorical variables were presented as frequencies and percentages. SD, standard deviations; BMI, body mass index.

**Supplemental Table 5 Baseline characteristics of participants by AR status**

|  | Overall  (N= 341,460) | non-AR  (n= 340,177) | AR  (n= 1,283) |
| --- | --- | --- | --- |
| Age, mean (SD), y | 56.89 (8.09) | 56.87 (8.09) | 61.82 (6.36) |
| Female, n (%) | 184603 (54.1) | 184049 (54.1) | 554 (43.2) |
| White, n (%) | 323205 (94.7) | 321992 (94.7) | 1213 (94.5) |
| **Income, n (%)** |  |  |  |
| <£18,000 | 81207 (23.8) | 80787 (23.7) | 420 (32.7) |
| £18,000 to £30,999 | 87942 (25.8) | 87568 (25.7) | 374 (29.2) |
| £31,000 to £51,999 | 87527 (25.6) | 87233 (25.6) | 294 (22.9) |
| £52,000 to £100,000 | 67167 (19.7) | 67000 (19.7) | 167 (13.0) |
| > £100,000 | 17617 (5.2) | 17589 (5.2) | 28 (2.2) |
| **Education level, n (%)** |  |  |  |
| College or University degree | 112295 (32.9) | 111946 (32.9) | 349 (27.2) |
| Others | 229165 (67.1) | 228231 (67.1) | 934 (72.8) |
| **Employment status, n (%)** |  |  |  |
| No | 140246 (41.1) | 139478 (41.0) | 768 (59.9) |
| Yes | 201214 (58.9) | 200699 (59.0) | 515 (40.1) |
| **Physical activity, n (%)** |  |  |  |
| Low | 63324 (18.5) | 63077 (18.5) | 247 (19.3) |
| Moderate | 139163 (40.8) | 138649 (40.8) | 514 (40.1) |
| High | 138973 (40.7) | 138451 (40.7) | 522 (40.7) |
| **Smoking status, n (%)** |  |  |  |
| Never | 188775 (55.3) | 188115 (55.3) | 660 (51.4) |
| Ever | 117640 (34.5) | 117134 (34.4) | 506 (39.4) |
| Current | 35045 (10.3) | 34928 (10.3) | 117 (9.1) |
| **Drinking status, n (%)** |  |  |  |
| Never | 14664 (4.3) | 14602 (4.3) | 62 (4.8) |
| Ever | 11614 (3.4) | 11542 (3.4) | 72 (5.6) |
| Current | 315182 (92.3) | 314033 (92.3) | 1149 (89.6) |
| BMI, kg/m^2^, mean (SD) | 27.34 (4.69) | 27.33 (4.69) | 28.29 (4.82) |
| Townsend deprivation index, mean (SD) | -1.37 (3.05) | -1.37 (3.05) | -1.19 (3.27) |
| **Biological ages, mean (SD)** |  |  |  |
| KDM-BA | 54.29 (9.30) | 54.27 (9.30) | 60.28 (7.57) |
| KDM-BA acceleration | -0.01 (4.83) | -0.01 (4.83) | 1.14 (5.34) |
| PhenoAge | 47.92 (10.00) | 47.89 (9.99) | 54.54 (8.67) |
| PhenoAge acceleration | -0.01 (5.36) | -0.01 (5.35) | 1.41 (6.11) |

Quantitative variables were presented as mean with SD and categorical variables were presented as frequencies and percentages. SD, standard deviations; BMI, body mass index.

**Supplemental Table 6 Baseline characteristics of participants by MR status**

|  | Overall  (N= 341,460) | non-MR  (n= 337,420) | MR  (n= 4,040) |
| --- | --- | --- | --- |
| Age, mean (SD), y | 56.89 (8.09) | 56.83 (8.09) | 61.80 (6.53) |
| Female, n (%) | 184603 (54.1) | 182833 (54.2) | 1770 (43.8) |
| White, n (%) | 323205 (94.7) | 319344 (94.6) | 3861 (95.6) |
| **Income, n (%)** |  |  |  |
| <£18,000 | 81207 (23.8) | 79876 (23.7) | 1331 (32.9) |
| £18,000 to £30,999 | 87942 (25.8) | 86793 (25.7) | 1149 (28.4) |
| £31,000 to £51,999 | 87527 (25.6) | 86591 (25.7) | 936 (23.2) |
| £52,000 to £100,000 | 67167 (19.7) | 66674 (19.8) | 493 (12.2) |
| > £100,000 | 17617 (5.2) | 17486 (5.2) | 131 (3.2) |
| **Education level, n (%)** |  |  |  |
| College or University degree | 112295 (32.9) | 111176 (32.9) | 1119 (27.7) |
| Others | 229165 (67.1) | 226244 (67.1) | 2921 (72.3) |
| **Employment status, n (%)** |  |  |  |
| No | 140246 (41.1) | 137792 (40.8) | 2454 (60.7) |
| Yes | 201214 (58.9) | 199628 (59.2) | 1586 (39.3) |
| **Physical activity, n (%)** |  |  |  |
| Low | 63324 (18.5) | 62562 (18.5) | 762 (18.9) |
| Moderate | 139163 (40.8) | 137539 (40.8) | 1624 (40.2) |
| High | 138973 (40.7) | 137319 (40.7) | 1654 (40.9) |
| **Smoking status, n (%)** |  |  |  |
| Never | 188775 (55.3) | 186799 (55.4) | 1976 (48.9) |
| Ever | 117640 (34.5) | 115996 (34.4) | 1644 (40.7) |
| Current | 35045 (10.3) | 34625 (10.3) | 420 (10.4) |
| **Drinking status, n (%)** |  |  |  |
| Never | 14664 (4.3) | 14492 (4.3) | 172 (4.3) |
| Ever | 11614 (3.4) | 11417 (3.4) | 197 (4.9) |
| Current | 315182 (92.3) | 311511 (92.3) | 3671 (90.9) |
| BMI, kg/m^2^, mean (SD) | 27.34 (4.69) | 27.33 (4.69) | 28.02 (4.89) |
| Townsend deprivation index, mean (SD) | -1.37 (3.05) | -1.37 (3.05) | -1.30 (3.12) |
| **Biological ages, mean (SD)** |  |  |  |
| KDM-BA | 54.29 (9.30) | 54.22 (9.29) | 60.23 (8.30) |
| KDM-BA acceleration | -0.01 (4.83) | -0.02 (4.82) | 1.12 (5.67) |
| PhenoAge | 47.92 (10.00) | 47.84 (9.98) | 54.54 (9.27) |
| PhenoAge acceleration | -0.01 (5.36) | -0.03 (5.34) | 1.41 (6.28) |

Quantitative variables were presented as mean with SD and categorical variables were presented as frequencies and percentages. SD, standard deviations; BMI, body mass index.

**Supplemental Table 7 Baseline characteristics of participants by KDM-BA acceleration (****Quartiles)**

|  | **Q1**  **(n=** **85365)** | | **Q2**  **(n=** **85365)** | | **Q3**  **(n=** 85365**)** | **Q4**  **(n=** **85365)** |
| --- | --- | --- | --- | --- | --- | --- |
| Age, mean (SD), y | 56.94 (8.02) | | 56.80 (8.16) | | 56.88 (8.15) | 56.94 (8.03) |
| Female, n (%) | 46251 (54.2) | | 44851 (52.5) | | 45400 (53.2) | 48101 (56.3) |
| White, n (%) | 83519 (97.8) | | 82155 (96.2) | | 80471 (94.3) | 77060 (90.3) |
| **Income, n (%)** |  | |  | |  |  |
| <£18,000 | 15452 (18.1) | | 17940 (21.0) | | 20936 (24.5) | 26879 (31.5) |
| £18,000 to £30,999 | 20728 (24.3) | | 21580 (25.3) | | 22289 (26.1) | 23345 (27.3) |
| £31,000 to £51,999 | 23008 (27.0) | | 22558 (26.4) | | 21989 (25.8) | 19972 (23.4) |
| £52,000 to £100,000 | 20014 (23.4) | | 18397 (21.6) | | 16193 (19.0) | 12563 (14.7) |
| > £100,000 | 6163 (7.2) | | 4890 (5.7) | | 3958 (4.6) | 2606 (3.1) |
| **Education level, n (%)** | |  | |  |  |  |
| College or University degree | 35529 (41.6) | | 30040 (35.2) | | 25788 (30.2) | 20938 (24.5) |
| Others | 49836 (58.4) | | 55325 (64.8) | | 59577 (69.8) | 64427 (75.5) |
| **Employment status, n (%)** |  | |  | |  |  |
| No | 33014 (38.7) | | 33820 (39.6) | | 35005 (41.0) | 38407 (45.0) |
| Yes | 52351 (61.3) | | 51545 (60.4) | | 50360 (59.0) | 46958 (55.0) |
| **Physical activity, n (%)** |  | |  | |  |  |
| Low | 13630 (16.0) | | 14558 (17.1) | | 15996 (18.7) | 19140 (22.4) |
| Moderate | 35339 (41.4) | | 34987 (41.0) | | 34407 (40.3) | 34430 (40.3) |
| High | 36396 (42.6) | | 35820 (42.0) | | 34962 (41.0) | 31795 (37.2) |
| **Smoking status, n (%)** |  | |  | |  |  |
| Never | 50006 (58.6) | | 48611 (56.9) | | 46535 (54.5) | 43623 (51.1) |
| Ever | 29548 (34.6) | | 29065 (34.0) | | 29406 (34.4) | 29621 (34.7) |
| Current | 5811 (6.8) | | 7689 (9.0) | | 9424 (11.0) | 12121 (14.2) |
| **Drinking status, n (%)** |  | |  | |  |  |
| Never | 2540 (3.0) | | 3075 (3.6) | | 3649 (4.3) | 5400 (6.3) |
| Ever | 2552 (3.0) | | 2574 (3.0) | | 2860 (3.4) | 3628 (4.2) |
| Current | 80273 (94.0) | | 79716 (93.4) | | 78856 (92.4) | 76337 (89.4) |
| BMI, kg/m^2^, mean (SD) | 25.08 (3.44) | | 26.53 (3.87) | | 27.80 (4.34) | 29.93 (5.45) |
| Townsend deprivation index, mean (SD) | -1.76 (2.82) | | -1.59 (2.93) | | -1.35 (3.05) | -0.78 (3.30) |
| **Biological ages, mean (SD)** |  | |  | |  |  |
| KDM-BA | 48.57 (8.12) | | 52.45 (8.07) | | 55.51 (8.06) | 60.64 (8.50) |
| KDM-BA acceleration | -5.78 (2.10) | | -1.76 (0.85) | | 1.21 (0.92) | 6.29 (3.17) |
| PhenoAge | 44.02 (9.16) | | 46.54 (9.35) | | 48.67 (9.45) | 52.43 (10.06) |
| PhenoAge acceleration | -3.95 (3.92) | | -1.29 (3.82) | | 0.74 (4.11) | 4.45 (5.50) |

Quantitative variables were presented as mean with SD and categorical variables were presented as frequencies and percentages. SD, standard deviations; BMI, body mass index; Q1: Quartile 1; Q2: Quartile 2; Q3: Quartile 3; Q4: Quartile 4.

**Supplemental Table 8 Baseline characteristics of participants by PhenoAge acceleration (Quartiles)**

|  | **Q1**  **(n=** **85365)** | | **Q2**  **(n=** **85365)** | | **Q3**  **(n=** 85365**)** | **Q4**  **(n=** **85365)** |
| --- | --- | --- | --- | --- | --- | --- |
| Age, mean (SD), y | 57.04 (8.06) | | 56.75 (8.11) | | 56.77 (8.11) | 56.99 (8.08) |
| Female, n (%) | 52764 (61.8) | | 48854 (57.2) | | 44419 (52.0) | 38566 (45.2) |
| White, n (%) | 83505 (97.8) | | 82413 (96.5) | | 81038 (94.9) | 76249 (89.3) |
| **Income, n (%)** |  | |  | |  |  |
| <£18,000 | 16641 (19.5) | | 18133 (21.2) | | 20010 (23.4) | 26423 (31.0) |
| £18,000 to £30,999 | 21333 (25.0) | | 21654 (25.4) | | 22093 (25.9) | 22862 (26.8) |
| £31,000 to £51,999 | 22833 (26.7) | | 22477 (26.3) | | 22273 (26.1) | 19944 (23.4) |
| £52,000 to £100,000 | 19028 (22.3) | | 18168 (21.3) | | 16789 (19.7) | 13182 (15.4) |
| > £100,000 | 5530 (6.5) | | 4933 (5.8) | | 4200 (4.9) | 2954 (3.5) |
| **Education level, n (%)** | |  | |  |  |  |
| College or University degree | 32300 (37.8) | | 29724 (34.8) | | 27245 (31.9) | 23026 (27.0) |
| Others | 53065 (62.2) | | 55641 (65.2) | | 58120 (68.1) | 62339 (73.0) |
| **Employment status, n (%)** |  | |  | |  |  |
| No | 34363 (40.3) | | 33595 (39.4) | | 34069 (39.9) | 38219 (44.8) |
| Yes | 51002 (59.7) | | 51770 (60.6) | | 51296 (60.1) | 47146 (55.2) |
| **Physical activity, n (%)** |  | |  | |  |  |
| Low | 13950 (16.3) | | 14777 (17.3) | | 15817 (18.5) | 18780 (22.0) |
| Moderate | 35188 (41.2) | | 34961 (41.0) | | 34610 (40.5) | 34404 (40.3) |
| High | 36227 (42.4) | | 35627 (41.7) | | 34938 (40.9) | 32181 (37.7) |
| **Smoking status, n (%)** |  | |  | |  |  |
| Never | 50515 (59.2) | | 49016 (57.4) | | 47369 (55.5) | 41875 (49.1) |
| Ever | 30066 (35.2) | | 29713 (34.8) | | 29062 (34.0) | 28799 (33.7) |
| Current | 4784 (5.6) | | 6636 (7.8) | | 8934 (10.5) | 14691 (17.2) |
| **Drinking status, n (%)** |  | |  | |  |  |
| Never | 2700 (3.2) | | 3151 (3.7) | | 3671 (4.3) | 5142 (6.0) |
| Ever | 2431 (2.8) | | 2531 (3.0) | | 2785 (3.3) | 3867 (4.5) |
| Current | 80234 (94.0) | | 79683 (93.3) | | 78909 (92.4) | 76356 (89.4) |
| BMI, kg/m^2^, mean (SD) | 25.90 (3.77) | | 26.83 (4.17) | | 27.65 (4.58) | 28.96 (5.50) |
| Townsend deprivation index, mean (SD) | -1.78 (2.81) | | -1.60 (2.91) | | -1.39 (3.02) | -0.69 (3.33) |
| **Biological ages, mean (SD)** |  | |  | |  |  |
| KDM-BA | 50.88 (8.83) | | 52.97 (8.73) | | 54.82 (8.70) | 58.50 (9.21) |
| KDM-BA acceleration | -3.58 (3.82) | | -1.20 (3.56) | | 0.64 (3.64) | 4.10 (4.64) |
| PhenoAge | 41.85 (8.65) | | 45.80 (8.50) | | 49.01 (8.53) | 55.00 (9.34) |
| PhenoAge acceleration | -6.24 (2.18) | | -1.98 (0.91) | | 1.21 (0.99) | 6.97 (3.90) |

Quantitative variables were presented as mean with SD and categorical variables were presented as frequencies and percentages. SD, standard deviations; BMI, body mass index; Q1: Quartile 1; Q2: Quartile 2; Q3: Quartile 3; Q4: Quartile 4.

**Supplemental Table 9 Characteristics of Components of biological age by VHD status**

|  | **Overall**  **(N=** **341460)** | **non-VHD**  **(n=** **333314)** | **VHD**  **(n=** **8146)** |
| --- | --- | --- | --- |
| FEV_1_ (L)* | 2.82 (0.80) | 2.83 (0.80) | 2.62 (0.78) |
| SBP (mm Hg) * | 137.76 (18.49) | 137.60 (18.44) | 144.55 (19.29) |
| Total Cholesterol (mg/dL) * | 220.49 (43.88) | 220.69 (43.76) | 212.16 (47.86) |
| Glycated hemoglobin (%) * | 35.07 (5.95) | 35.02 (5.88) | 37.17 (8.15) |
| Blood urea nitrogen (μmol) * | 15.15 (3.79) | 15.12 (3.74) | 16.64 (5.40) |
| Lymphocyte (%) * | 29.00 (7.42) | 29.03 (7.40) | 27.76 (8.00) |
| Mean cell volume (fL) ^#^ | 82.81 (5.28) | 82.80 (5.27) | 83.08 (5.56) |
| Serum glucose (μmol) ^#^ | 5.11 (1.20) | 5.10 (1.18) | 5.38 (1.66) |
| Red cell distribution width (%) ^#^ | 13.47 (0.95) | 13.47 (0.95) | 13.63 (0.99) |
| White blood cell count (1000 cells/μL) ^#^ | 6.86 (1.98) | 6.85 (1.98) | 7.21 (2.28) |
| Albumin (g/dL) ^*#^ | 4.53 (0.26) | 4.53 (0.26) | 4.48 (0.27) |
| Creatinine (μmol) ^*#^ | 72.13 (15.41) | 72.01 (15.16) | 76.94 (23.00) |
| C-reactive protein (μmol) ^*#^ | 0.25 (0.41) | 0.25 (0.41) | 0.33 (0.54) |
| Alkaline phosphatase (U/L) ^*#^ | 83.19 (25.83) | 83.09 (25.69) | 87.42 (30.72) |

^*^Employed to construct KDM-BA.

^#^Employed to construct PhenoAge.

FEV_1_: Forced expiratory volume in one second; SBP: Systolic blood pressure

**Supplemental Table 10 Associations of the biological age accelerations with the risk of AS**

| **Biological age** | **Cases/**  **Person years** | **Incidence rate (95%CI), per 1000 person-years** | **HR (95%CI)** | ***P*** | **PAF (95%CI)** |
| --- | --- | --- | --- | --- | --- |
| KDM-BA acceleration (Continuous) | 3479/4531716.7 | 0.77 (0.74, 0.79) | 1.49 (1.45, 1.54) | <0.001 |  |
| KDM-BA acceleration (Quartiles) | | | | | |
| Q1 | 447/1146290.6 | 0.39 (0.35, 0.43) | Reference |  |  |
| Q2 | 673/1140918.9 | 0.59 (0.55, 0.64) | 1.38 (1.23, 1.56) | <0.001 | 20.18% (14.73%, 25.58%) |
| Q3 | 867/1134324.8 | 0.76 (0.71, 0.82) | 1.68 (1.49, 1.88) | <0.001 | 31.96% (27.10%, 36.98%) |
| Q4 | 1492/1110182.3 | 1.34 (1.28, 1.41) | 2.68 (2.40, 3.00) | <0.001 | 53.89% (50.40%, 57.49%) |
|  |  |  |  |  |  |
| PhenoAge acceleration (Continuous) | 3479/4531716.7 | 0.77 (0.74, 0.79) | 1.34 (1.29, 1.38) | <0.001 |  |
| PhenoAge acceleration (Quartiles) | | | | | |
| Q1 | 539/1150257.7 | 0.47 (0.43, 0.51) | Reference |  |  |
| Q2 | 665/1145273.5 | 0.58 (0.54, 0.63) | 1.14 (1.02, 1.28) | 0.026 | 10.47% (5.14%, 16.23%) |
| Q3 | 860/1136182.8 | 0.76 (0.71, 0.81) | 1.34 (1.20, 1.49) | <0.001 | 22.94% (17.86%, 27.99%) |
| Q4 | 1415/1100002.7 | 1.29 (1.22, 1.36) | 1.92 (1.73, 2.13) | <0.001 | 44.83% (40.92%, 48.62%) |

Model adjusted for age, sex, ethnicity, education level, employment status, BMI, smoking status, drinking status, physical activity, and Townsend deprivation index. Q1: Quartile 1; Q2: Quartile 2; Q3: Quartile 3; Q4: Quartile 4

.

**Supplemental Table 11 Associations of the biological age accelerations with the risk of AR**

| **Biological age** | **Cases/**  **Person years** | **Incidence rate (95%CI), per 1000 person-years** | **HR (95%CI)** | ***P*** | **PAF (95%CI)** |
| --- | --- | --- | --- | --- | --- |
| KDM-BA acceleration (Continuous) | 1283/4540934.1 | 0.28 (0.27, 0.30) | 1.24 (1.17, 1.31) | <0.001 |  |
| KDM-BA acceleration (Quartiles) | | | | | |
| Q1 | 240/1147157.3 | 0.21 (0.18, 0.24) | Reference |  |  |
| Q2 | 296/1142634.3 | 0.26 (0.23, 0.29) | 1.20 (1.01, 1.43) | 0.034 | 10.45% (2.36%, 18.71%) |
| Q3 | 316/1136661.3 | 0.28 (0.25, 0.31) | 1.26 (1.06, 1.49) | 0.001 | 13.67% (4.95%, 21.82%) |
| Q4 | 431/1114481.3 | 0.39 (0.35, 0.43) | 1.70 (1.43, 2.01) | 28.461 | 28.46% (21.26%, 35.54%) |
|  |  |  |  |  |  |
| PhenoAge acceleration (Continuous) | 1283/4540934.1 | 0.28 (0.27, 0.30) | 1.24 (1.18, 1.31) | <0.001 |  |
| PhenoAge acceleration (Quartiles) | | | | | |
| Q1 | 251/1151494.8 | 0.22 (0.19, 0.25) | Reference |  |  |
| Q2 | 278/1147018.7 | 0.24 (0.21, 0.27) | 1.08 (0.91, 1.29) | 0.358 | 5.10% (-3.41%, 14.62%) |
| Q3 | 299/1138631.8 | 0.26 (0.23, 0.29) | 1.12 (0.95, 1.33) | 0.189 | 8.73% (0.29%, 17.33%) |
| Q4 | 455/1103788.8 | 0.41 (0.38, 0.45) | 1.63 (1.38, 1.92) | <0.001 | 28.90% (21.83%, 36.33%) |

Model adjusted for age, sex, ethnicity, education level, employment status, BMI, smoking status, drinking status, physical activity, and Townsend deprivation index. Q1: Quartile 1; Q2: Quartile 2; Q3: Quartile 3; Q4: Quartile 4.

**Supplemental Table 12 Associations of the biological age accelerations with the risk of MR**

| **Biological age** | **Cases/**  **Person years** | **Incidence rate (95%CI), per 1000 person-years** | **HR (95%CI)** | ***P*** | **PAF (95%CI)** |
| --- | --- | --- | --- | --- | --- |
| KDM-BA acceleration (Continuous) | 4040/4528800.6 | 0.89 (0.86, 0.92) | 1.27 (1.23, 1.31) | <0.001 |  |
| KDM-BA acceleration (Quartiles) | | | | | |
| Q1 | 862/1144244.8 | 0.75 (0.70, 0.81) | Reference |  |  |
| Q2 | 857/1140194.9 | 0.75 (0.70,0.80) | 0.98 (0.89, 1.08) | 0.703 | -0.29% (-4.90%, 4.07%) |
| Q3 | 1030/1133255.2 | 0.91 (0.85, 0.97) | 1.17 (1.07, 1.28) | 0.001 | 8.88% (4.22%, 13.35%) |
| Q4 | 1291/1111105.8 | 1.16 (1.10, 1.23) | 1.48 (1.35, 1.63) | <0.001 | 19.93% (16.05%, 23.96%) |
|  |  |  |  |  |  |
| PhenoAge acceleration (Continuous) | 4040/4528800.6 | 0.89 (0.86, 0.92) | 1.27 (1.23, 1.31) | <0.001 |  |
| PhenoAge acceleration (Quartiles) | | | | | |
| Q1 | 825/1148857.1 | 0.72 (0.67, 0.77) | Reference |  |  |
| Q2 | 869/1144415.1 | 0.76 (0.71, 0.81) | 1.04 (0.95, 1.15) | 0.375 | 2.60% (-1.94%, 7.23%) |
| Q3 | 948/1135555.3 | 0.83 (0.78, 0.89) | 1.11 (1.01, 1.22) | 0.032s | 6.94% (2.53%, 11.58%)) |
| Q4 | 1398/1099973.2 | 1.27 (1.21, 1.34) | 1.59 (1.45, 1.74) | <0.001 | 25.78% (21.73%, 29.94%) |

Model adjusted for age, sex, ethnicity, education level, employment status, BMI, smoking status, drinking status, physical activity, and Townsend deprivation index. Q1: Quartile 1; Q2: Quartile 2; Q3: Quartile 3; Q4: Quartile 4.

**Supplemental Table 13 Association between genetic predisposition and incident VHD**

|  |  | **HR (95% CI)** | ***P*** |
| --- | --- | --- | --- |
| PRS group | Low | Reference |  |
|  | Intermediate | 1.30 (1.21, 1.39) | <0.001 |
|  | High | 1.69 (1.57, 1.82) | <0.001 |

Model adjusted for age, sex, ethnicity, education level, employment status, BMI, smoking status, drinking status, physical activity, and Townsend deprivation index.

**Supplemental Table 14 Associations of biological age (Continuous) with incident VHD within each genetic risk category**

| **Subgroup** | **Cases/Person years** | **HR (95%CI)** | ***P*** |
| --- | --- | --- | --- |
| **KDM-BA acceleration (Continuous)** | | | |
| Low genetic risk | 1086/758356.8 | 1.34 (1.26, 1.43) | <0.001 |
| Intermediate genetic risk | 4164/2269204.6 | 1.35 (1.31, 1.40) | <0.001 |
| High genetic risk | 1776/753739.5 | 1.33 (1.27, 1.40) | <0.001 |
|  |  |  |  |
| **PhenoAge acceleration (Continuous)** | | | |
| Low genetic risk | 1086/758356.8 | 1.29 (1.22, 1.37) | <0.001 |
| Intermediate genetic risk | 4164/2269204.6 | 1.31 (1.27, 1.35) | <0.001 |
| High genetic risk | 1776/753739.5 | 1.26 (1.20, 1.32) | <0.001 |

Model adjusted for age, sex, education level, employment status, BMI, smoking status, drinking status, physical activity, and Townsend deprivation index.

**Supplemental Table 15 Associations of biological age (Continuous) with incident AS within each genetic risk category**

| **Subgroup** | **Cases/Person years** | **HR (95%CI)** | ***P*** |
| --- | --- | --- | --- |
| **KDM-BA acceleration (Continuous)** | | | |
| Low genetic risk | 362/761248.9 | 1.67 (1.52, 1.84) | <0.001 |
| Intermediate genetic risk | 1747/2279251.9 | 1.46 (1.40, 1.53) | <0.001 |
| High genetic risk | 950/757146.3 | 1.39 (1.30, 1.48) | <0.001 |
|  |  |  |  |
| **PhenoAge acceleration (Continuous)** | | | |
| Low genetic risk | 362/761248.9 | 1.41 (1.28, 1.55) | <0.001 |
| Intermediate genetic risk | 1747/2279251.9 | 1.34 (1.28, 1.4) | <0.001 |
| High genetic risk | 950/757146.3 | 1.28 (1.20, 1.36) | <0.001 |

Model adjusted for age, sex, education level, employment status, BMI, smoking status, drinking status, physical activity, and Townsend deprivation index.

**Supplemental Table 16 Associations of biological age (Continuous) with incident AR within each genetic risk category**

| **Subgroup** | **Cases/Person years** | **HR (95%CI)** | ***P*** |
| --- | --- | --- | --- |
| **KDM-BA acceleration (Continuous)** | | | |
| Low genetic risk | 190/761865.3 | 1.33 (1.15, 1.54) | <0.001 |
| Intermediate genetic risk | 633/2283908.8 | 1.19 (1.09, 1.29) | <0.001 |
| High genetic risk | 243/760308.1 | 1.30 (1.14, 1.49) | <0.001 |
|  |  |  |  |
| **PhenoAge acceleration (Continuous)** | | | |
| Low genetic risk | 190/761865.3 | 1.29 (1.12, 1.48) | <0.001 |
| Intermediate genetic risk | 633/2283908.8 | 1.19 (1.10, 1.29) | <0.001 |
| High genetic risk | 243/760308.1 | 1.27 (1.11, 1.44) | <0.001 |

Model adjusted for age, sex, education level, employment status, BMI, smoking status, drinking status, physical activity, and Townsend deprivation index.

**Supplemental Table 17 Associations of biological age (Continuous) with incident MR within each genetic risk category**

| **Subgroup** | **Cases/Person years** | **HR (95%CI)** | ***P*** |
| --- | --- | --- | --- |
| **KDM-BA acceleration (Continuous)** | | | |
| Low genetic risk | 613/760019.9 | 1.16 (1.06, 1.26) | <0.001 |
| Intermediate genetic risk | 2118/2277329.4 | 1.30 (1.25, 1.36) | <0.001 |
| High genetic risk | 750/758062.2 | 1.30 (1.200, 1.4) | <0.001 |
|  |  |  |  |
| **PhenoAge acceleration (Continuous)** | | | |
| Low genetic risk | 613/760019.9 | 1.22 (1.13, 1.33) | <0.001 |
| Intermediate genetic risk | 2118/2277329.4 | 1.31 (1.26, 1.37) | <0.001 |
| High genetic risk | 750/758062.2 | 1.26 (1.18, 1.36) | <0.001 |

Model adjusted for age, sex, education level, employment status, BMI, smoking status, drinking status, physical activity, and Townsend deprivation index.

**Supplemental Table 18 Additive interaction between biological age acceleration and genetic risk categories on incident AS**

|  | | **Intermediate genetic risk** | |  | **High genetic risk** | |
| --- | --- | --- | --- | --- | --- | --- |
|  |  | **RERI (95%CI)** | **AP (95%CI)** |  | **RERI (95%CI)** | **AP (95%CI)** |
| KDM-BA acceleration (Quartiles) | Q2 | 0.26 (-0.29, 0.80) | 0.10 (-0.12, 0.33) |  | 0.69 (-0.25, 1.62) | 0.15 (-0.05, 0.34) |
|  | Q3 | 0.75 (0.22, 1.27) | 0.23 (0.05, 0.41) |  | 0.68 (-0.26, 1.62) | 0.14 (-0.05, 0.33) |
|  | Q4 | 0.57 (-0.10, 1.24） | 0.12 (-0.03, 0.27) |  | 1.47 (0.37, 2.57) | 0.20 (0.06, 0.34) |
|  |  |  |  |  |  |  |
| PhenoAge acceleration (Quartiles) | Q2 | 0.21 (-0.20, 0.63) | 0.12 (-0.12, 0.36) |  | 0.40 (-0.29, 1.09) | 0.13 (-0.09, 0.34) |
|  | Q3 | 0.38 (-0.03, 0.78) | 0.18 (-0.03, 0.38) |  | 0.66 (-0.03, 1.34) | 0.18 (-0.01, 0.37) |
|  | Q4 | 0.38 (-0.09, 0.84) | 0.12 (-0.04, 0.28) |  | 0.83 (0.09, 1.57) | 0.18 (0.02, 0.33) |

RERI: relative excess risk of interaction; AP: attributable proportion. RERI and AP were calculated by “Delta” method. Q1: Quartile 1; Q2: Quartile 2; Q3: Quartile 3; Q4: Quartile 4

**Supplemental Table 19 Additive interaction between biological age acceleration and genetic risk categories on incident AR**

|  | | **Intermediate genetic risk** | |  | **High genetic risk** | |
| --- | --- | --- | --- | --- | --- | --- |
|  |  | **RERI (95%CI)** | **AP (95%CI)** |  | **RERI (95%CI)** | **AP (95%CI)** |
| KDM-BA acceleration (Quartiles) | Q2 | 0.37 (-0.14, 0.87) | 0.24 (-0.12, 0.60) |  | 0.02 (-0.73, 0.76) | 0.01 (-0.48, 0.50) |
|  | Q3 | 0.15 (-0.41, 0.72) | 0.10 (-0.27, 0.47) |  | -0.08 (-0.87, 0.71) | -0.05 (-0.52, 0.42) |
|  | Q4 | -0.44 (-1.20, 0.33） | -0.23 (-0.61, 0.15) |  | -0.24 (-1.17, 0.69) | -0.10 (-0.48, 0.28) |
|  |  |  |  |  |  |  |
| PhenoAge acceleration (Quartiles) | Q2 | -0.05 (-0.62, 0.53) | -0.03 (-0.45, 0.39) |  | -0.06 (-0.80, 0.69) | -0.04 (-0.55, 0.47) |
|  | Q3 | -0.38 (-1.04, 0.29) | -0.29 (-0.76, 0.18) |  | -0.39 (-1.20, 0.42) | -0.28 (-0.85, 0.30) |
|  | Q4 | -0.21 (-0.89, 0.47) | -0.11 (-0.45, 0.23) |  | 0.31 (-0.52, 1.13) | 0.12 (-0.20, 0.44) |

RERI: relative excess risk of interaction; AP: attributable proportion. RERI and AP were calculated by “Delta” method. Q1: Quartile 1; Q2: Quartile 2; Q3: Quartile 3; Q4: Quartile 4

**Supplemental Table 20 Additive interaction between biological age acceleration and genetic risk categories on incident MR**

|  | | **Intermediate genetic risk** | |  | **High genetic risk** | |
| --- | --- | --- | --- | --- | --- | --- |
|  |  | **RERI (95%CI)** | **AP (95%CI)** |  | **RERI (95%CI)** | **AP (95%CI)** |
| KDM-BA acceleration (Quartiles) | Q2 | 0.13 (-0.11, 0.38) | 0.13 (-0.12, 0.37) |  | 0.14 (-0.18, 0.46) | 0.13 (-0.16, 0.41) |
|  | Q3 | 0.05 (-0.22, 0.32) | 0.04 (-0.18, 0.26) |  | 0.21 (-0.13, 0.55) | 0.15 (-0.09, 0.38) |
|  | Q4 | 0.32 (0.05, 0.58） | 0.19 (0.02, 0.37) |  | 0.33 (-0.02, 0.68) | -0.20 (-0.01, 0.40) |
|  |  |  |  |  |  |  |
| PhenoAge acceleration (Quartiles) | Q2 | -0.11 (-0.43, 0.21) | -0.08 (-0.33, 0.16) |  | -0.26 (-0.68, 0.15) | -0.20 (-0.52, 0.12) |
|  | Q3 | -0.20 (-0.54, 0.14) | -0.15 (-0.39, 0.09) |  | -0.25 (-0.67, 0.18) | -0.17 (-0.46, 0.12) |
|  | Q4 | 0.28 (-0.04, 0.59) | 0.14 (-0.03, 0.30) |  | 0.20 (-0.23, 0.62) | 0.09 (-0.11, 0.29) |

RERI: relative excess risk of interaction; AP: attributable proportion. RERI and AP were calculated by “Delta” method. Q1: Quartile 1; Q2: Quartile 2; Q3: Quartile 3; Q4: Quartile 4

**Supplemental Table 21 Stratified analyses for associations between biological age accelerations and the risk of VHD**

| **Group** | | **Cases/Person** | **KDM-BA acceleration** | | |  | **PhenoAge acceleration** | | |
| --- | --- | --- | --- | --- | --- | --- | --- | --- | --- |
|  |  |  | **HR (95%CI)** | ***P*** | ***P* _interaction_** |  | **HR (95%CI)** | ***P*** | ***P* _interaction_** |
| **Sex** | Male | 3338/2462540.8 | 1.41 (1.37, 1.45) | <0.001 | 0.002 |  | 1.30 (1.26, 1.33) | <0.001 | 0.687 |
|  | Female | 4808/2049906.6 | 1.28 (1.24, 1.33) | <0.001 |  |  | 1.29 (1.25, 1.34) | <0.001 |  |
| **Age** | <65 years | 4743/3713809.8 | 1.36 (1.32, 1.40) | <0.001 | 0.023 |  | 1.30 (1.27, 1.34) | <0.001 | 0.005 |
|  | ≥65 years | 3403/798637.7 | 1.29 (1.25, 1.34) | <0.001 |  |  | 1.26 (1.22, 1.30) | <0.001 |  |
| **Ethnicity** | White | 7824/4273980.5 | 1.35 (1.32, 1.38) | <0.001 | 0.655 |  | 1.30 (1.27, 1.33) | <0.001 | 0.089 |
|  | Other | 322/238466.9 | 1.38 (1.25, 1.53) | <0.001 |  |  | 1.20 (1.10, 1.32) | <0.001 |  |
| **BMI** | <25 | 1939/1519073 | 1.34 (1.28, 1.40) | <0.001 | 0.062 |  | 1.28 (1.22, 1.34) | <0.001 | 0.103 |
|  | ≥25 | 6207/2993374.4 | 1.41 (1.38, 1.45) | <0.001 |  |  | 1.34 (1.31, 1.37) | <0.001 |  |
| **Employment status** | Yes | 5165/1807775.3 | 1.36 (1.31, 1.41) | <0.001 | 0.663 |  | 1.26 (1.21, 1.30) | <0.001 | 0.094 |
|  | No | 2981/2704672.2 | 1.35 (1.31, 1.38) | <0.001 |  |  | 1.31 (1.27, 1.34) | <0.001 |  |
| **smoking status** | Never | 3754/2518729.3 | 1.36 (1.32, 1.41) | <0.001 | 0.266 |  | 1.29 (1.25, 1.33) | <0.001 | 0.671 |
|  | Ever | 3493/1540682.8 | 1.33 (1.28, 1.37) | <0.001 |  |  | 1.28 (1.24, 1.32) | <0.001 |  |
|  | Now | 899/453035.3 | 1.41 (1.33, 1.51) | <0.001 |  |  | 1.32 (1.25, 1.40) | <0.001 |  |
| **Drinking status** | Never | 359/192240.3 | 1.38 (1.26, 1.53) | <0.001 | 0.136 |  | 1.33 (1.21, 1.45) | <0.001 | 0.396 |
|  | Ever | 415/149023.1 | 1.47 (1.35, 1.60) | <0.001 |  |  | 1.39 (1.29, 1.41) | <0.001 |  |
|  | Now | 7372/4171184 | 1.34 (1.31, 1.38) | <0.001 |  |  | 1.29 (1.26, 1.32) | <0.001 |  |
| **Physical activity** | Low | 1651/833354.8 | 1.41 (1.35, 1.48) | <0.001 | 0.027 |  | 1.34 (1.28, 1.40) | <0.001 | 0.042 |
|  | Moderate | 3233/1840140 | 1.34 (1.29, 1.38) | <0.001 |  |  | 1.28 (1.24, 1.32) | <0.001 |  |
|  | High | 3262/1838952.7 | 1.33 (1.28, 1.38) | <0.001 |  |  | 1.27 (1.23, 1.32) | <0.001 |  |

BMI: Body Mass Index; Model adjusted for age (not for age-stratified analysis), sex (not for sex-stratified analysis), ethnicity (not for ethnicity-stratified analysis), education level, employment status (not for employment status -stratified analysis), BMI (not for BMI -stratified analysis), smoking status (not for smoking status -stratified analysis) , drinking status (not for drinking status -stratified analysis), physical activity (not for physical activity -stratified analysis), and Townsend deprivation index.

**Supplemental Table 22 Associations of the biological age accelerations with the risk of VHD among individuals without missing covariate data**

| **Biological age** | **Cases/**  **Person years** | **Incidence rate (95%CI), per 1000 person-years** | **HR (95%CI)** | ***P*** | **PAF (95%CI)** |
| --- | --- | --- | --- | --- | --- |
| KDM-BA acceleration (Continuous) | 8146/4512447.4 | 1.81 (1.77, 1.84) | 1.36 (1.33, 1.40) | <0.001 |  |
| KDM-BA acceleration (Quartiles) | | | | | |
| Q1 | 1445/1142018.4 | 1.27 (1.20, 1.33) | Reference |  |  |
| Q2 | 1702/1136599.6 | 1.50 (1.43, 1.57) | 1.11 (1.03, 1.21) | 0.007 | 8.17% (4.84%, 11.49%) |
| Q3 | 2038/1129286.9 | 1.80 (1.73, 1.88) | 1.34 (1.24, 1.44) | <0.001 | 17.03% (13.80%, 20.41%) |
| Q4 | 2961/1104542.5 | 2.68 (2.59, 2.78) | 1.88 (1.74, 2.03) | <0.001 | 34.41% (31.78%, 37.04%) |
|  |  |  |  |  |  |
| PhenoAge acceleration (Continuous) | 8146/4512447.4 | 1.81 (1.77, 1.84) | 1.30 (1.27, 1.33) | <0.001 |  |
| PhenoAge acceleration (Quartiles) | | | | | |
| Q1 | 1524/1146118.2 | 1.33 (1.26, 1.40) | Reference |  |  |
| Q2 | 1683/1141056.3 | 1.47 (1.41, 1.55) | 1.09 (1.01, 1.18) | 0.030 | 4.96% (1.79%, 8.43%) |
| Q3 | 1932/1131566.5 | 1.71 (1.63, 1.79) | 1.17 (1.09, 1.27) | <0.001 | 11.81% (8,46%, 15.25%) |
| Q4 | 3007/1093706.4 | 2.75 (2.65, 2.85) | 1.70 (1.58, 1.84) | <0.001 | 32.73% (30.91%, 35.58%) |

Model adjusted for age, sex, ethnicity, education level, employment status, BMI, smoking status, drinking status, physical activity, and Townsend deprivation index. Q1: Quartile 1; Q2: Quartile 2; Q3: Quartile 3; Q4: Quartile 4.

**Supplemental Table 23 Joint effects of biological age accelerations and PRS on the risk of VHD among individuals without missing covariate data**

| **Group** | **Cases/Person years** | | **HR (95%CI)** | ***P*** |
| --- | --- | --- | --- | --- |
| **Low genetic risk** | | | | |
| KDM-BA acceleration | Q1 | 211/199863.7 | Reference |  |
|  | Q2 | 219/194381.2 | 1.07 (0.86, 1.31) | 0.556 |
|  | Q3 | 268/188392.6 | 1.28 (1.04, 1.56) | 0.018 |
|  | Q4 | 388/175719.4 | 1.79 (1.47, 2.17) | <0.001 |
| **Intermediate genetic risk** | | | | |
| KDM-BA acceleration | Q1 | 743/594299.7 | 1.21 (1.02, 1.44) | 0.026 |
|  | Q2 | 894/579512.9 | 1.40 (1.18, 1.65) | <0.001 |
|  | Q3 | 1059/566632.3 | 1.69 (1.43, 1.99) | <0.001 |
|  | Q4 | 1468/528759.8 | 2.34 (1.98, 2.76) | <0.001 |
| **High genetic risk** | | | | |
| KDM-BA acceleration | Q1 | 320/190847.6 | 1.72 (1.42, 2.08) | <0.001 |
|  | Q2 | 384/194241.1 | 1.83 (1.52, 2.21) | <0.001 |
|  | Q3 | 451/189826.4 | 2.21 (1.84, 2.65) | <0.001 |
|  | Q4 | 621/178824.4 | 3.06 (2.56, 3.66) | <0.001 |
|  | | | | |
| **Low genetic risk** | | | | |
| PhenoAge acceleration | Q1 | 198/197055.1 | Reference |  |
|  | Q2 | 232/195665.3 | 1.16 (0.94, 1.44) | 0.163 |
|  | Q3 | 271/191019 | 1.28 (1.04, 1.58) | 0.018 |
|  | Q4 | 385/174617.5 | 1.74 (1.43, 2.12) | <0.001 |
| **Intermediate genetic risk** | | | | |
| PhenoAge acceleration | Q1 | 803/595588.8 | 1.38 (1.15, 1.64) | <0.001 |
|  | Q2 | 867/584667.6 | 1.46 (1.23, 1.74) | <0.001 |
|  | Q3 | 986/572355.2 | 1.58 (1.33, 1.88) | <0.001 |
|  | Q4 | 1508/516593.1 | 2.31 (1.95, 2.74) | <0.001 |
| **High genetic risk** | | | | |
| PhenoAge acceleration | Q1 | 357/198880.8 | 1.88 (1.55, 2.29) | <0.001 |
|  | Q2 | 375/195030 | 1.96 (1.61, 2.37) | <0.001 |
|  | Q3 | 421/189359.5 | 2.05 (1.7, 2.49) | <0.001 |
|  | Q4 | 623/170469.3 | 3.10 (2.58, 3.72) | <0.001 |

Model adjusted for age, sex, ethnicity, education level, employment status, BMI, smoking status, drinking status, physical activity, and Townsend deprivation index. Q1: Quartile 1; Q2: Quartile 2; Q3: Quartile 3; Q4: Quartile 4.

**Supplemental Table 24 Associations of biological age accelerations with incident VHD in different genetic risk backgrounds among individuals without missing covariate data**

| **Group** | **Cases/Person years** | | **HR (95%CI)** | ***P*** | |
| --- | --- | --- | --- | --- | --- |
| **Low genetic risk** | | | | | |
| KDM-BA acceleration (continue) | | 1086/758356.8 | 1.36 (1.26, 1.46) | | <0.001 |
| KDM-BA acceleration | Q1 | 211/199863.7 | Reference |  | |
|  | Q2 | 219/194381.2 | 1.08 (0.88, 1.34) | 0.463 | |
|  | Q3 | 268/188392.6 | 1.32 (1.07, 1.62) | 0.009 | |
|  | Q4 | 388/175719.4 | 1.90 (1.54, 2.33) | <0.001 | |
| **Intermediate genetic risk** | | | | | |
| KDM-BA acceleration (continue) | | 4164/2269204.7 | 1.35 (1.31, 1.4) | | <0.001 |
| KDM-BA acceleration | Q1 | 743/594299.7 | Reference |  | |
|  | Q2 | 894/579512.9 | 1.17 (1.06, 1.29) | 0.002 | |
|  | Q3 | 1059/566632.3 | 1.35 (1.22, 1.48) | <0.001 | |
|  | Q4 | 1468/528759.8 | 1.87 (1.7, 2.05) | <0.001 | |
| **High genetic risk** | | | | | |
| KDM-BA acceleration (continue) | | 1776/753739.5 | 1.33 (1.27, 1.4) | | <0.001 |
| KDM-BA acceleration | Q1 | 320/190847.6 | Reference |  | |
|  | Q2 | 384/194241.1 | 1.12 (0.97, 1.3) | 0.134 | |
|  | Q3 | 451/189826.4 | 1.29 (1.11, 1.49) | 0.001 | |
|  | Q4 | 621/178824.4 | 1.81 (1.57, 2.09) | <0.001 | |
|  | | | | | |
| **Low genetic risk** | | | | | |
| PhenoAge acceleration (continue) | | 1086/758356.8 | 1.31 (1.22, 1.40) | | <0.001 |
| PhenoAge acceleration | Q1 | 198/197055.1 | Reference |  | |
|  | Q2 | 232/195665.3 | 1.19 (0.96, 1.47) | 0.121 | |
|  | Q3 | 271/191019 | 1.33 (1.07, 1.63) | 0.008 | |
|  | Q4 | 385/174617.5 | 1.85 (1.51, 2.27) | <0.001 | |
| **Intermediate genetic risk** | | | | | |
| PhenoAge acceleration (continue) | | 4164/2269204.7 | 1.31 (1.27, 1.35) | | <0.001 |
| PhenoAge acceleration | Q1 | 803/595588.8 | Reference |  | |
|  | Q2 | 867/584667.6 | 1.05 (0.95, 1.15) | 0.365 | |
|  | Q3 | 986/572355.2 | 1.13 (1.03, 1.25) | 0.009 | |
|  | Q4 | 1508/516593.1 | 1.67 (1.53, 1.83) | <0.001 | |
| **High genetic risk** | | | | | |
| PhenoAge acceleration (continue) | | 1776/753739.5 | 1.26 (1.2, 1.32) | | <0.001 |
| PhenoAge acceleration | Q1 | 357/198880.8 | Reference |  | |
|  | Q2 | 375/195030 | 1.01(0.87, 1.17) | 0.878 | |
|  | Q3 | 421/189359.5 | 1.09(0.94, 1.25) | 0.253 | |
|  | Q4 | 623/170469.3 | 1.59(1.38, 1.82) | <0.001 | |

Model adjusted for age, sex, ethnicity, education level, employment status, BMI, smoking status, drinking status, physical activity, and Townsend deprivation index. Q1: Quartile 1; Q2: Quartile 2; Q3: Quartile 3; Q4: Quartile 4.

**Supplemental Table 25 Additive interaction between biological age acceleration and genetic risk categories on incident VHD among individuals without missing covariate data**

|  | | **Intermediate genetic risk** | |  | **High genetic risk** | |
| --- | --- | --- | --- | --- | --- | --- |
|  |  | **RERI (95%CI)** | **AP (95%CI)** |  | **RERI (95%CI)** | **AP (95%CI)** |
| KDM-BA acceleration (Quartiles) | Q2 | 0.12 (-0.13, 0.37) | 0.08 (-0.10, 0.27) |  | 0.05 (-0.31, 0.41) | 0.03 (-0.17, 0.22) |
|  | Q3 | 0.20 (-0.06, 0.46) | 0.12 (-0.04, 0.28) |  | 0.21 (-0.16, 0.58) | 0.10 (-0.07, 0.26) |
|  | Q4 | 0.34 (0.05, 0.62） | 0.14 (0.02, 0.27) |  | 0.56 (0.15, 0.97) | 0.18 (0.05, 0.31) |
|  |  |  |  |  |  |  |
| PhenoAge acceleration (Quartiles) | Q2 | -0.08 (-0.36, 0.21) | -0.05 (-0.24, 0.14) |  | -0.09 (-0.48, 0.30) | -0.05 (-0.25, 0.15) |
|  | Q3 | -0.08 (-0.37, 0.21) | -0.05 (-0.23, 0.13) |  | -0.11 (-0.51, 0.28) | -0.05 (-0.25, 0.14) |
|  | Q4 | 0.20 (-0.10, 0.49) | 0.08 (-0.05, 0.22) |  | 0.47 (0.06, 0.89) | 0.15 (0.02, 0.29) |

Model adjusted for age, sex, ethnicity, education level, employment status, BMI, smoking status, drinking status, physical activity, and Townsend deprivation index. Q1: Quartile 1; Q2: Quartile 2; Q3: Quartile 3; Q4: Quartile 4.

**Supplemental Table 26 Associations of biological ages with incident VHD after excluding participants with VHD in the first 2 years**

| **Biological age** | **Cases/**  **Person years** | **Incidence rate (95%CI), per 1000 person-years** | **HR (95%CI)** | ***P*** | **PAF (95%CI)** |
| --- | --- | --- | --- | --- | --- |
| KDM-BA acceleration (Continuous) | 7737/4511982.1 | 1.71 (1.68, 1.75) | 1.34 (1.31, 1.38) | <0.001 |  |
| KDM-BA acceleration (Quartiles) | | | | | |
| Q1 | 1379/1141941 | 1.21 (1.14, 1.27) | Reference |  |  |
| Q2 | 1623/1136506.3 | 1.43 (1.36, 1.50) | 1.13 (1.05, 1.21) | 0.007 | 8.13% (4.76%, 11.51%) |
| Q3 | 1935/1129176.3 | 1.71 (1.64, 1.79) | 1.30 (1.21, 1.40) | <0.001 | 16.80% (13.33%, 20.09%) |
| Q4 | 2800/1104358.5 | 2.54 (2.44, 2.63) | 1.84 (1.72, 1.98) | <0.001 | 34.04% (31.08%, 36.69%) |
|  |  |  |  |  |  |
| PhenoAge acceleration (Continuous) | 7737/4511982.1 | 1.71 (1.68, 1.75) | 1.28 (1.25, 1.31) | <0.001 |  |
| PhenoAge acceleration (Quartiles) | | | | | |
| Q1 | 1456/1146029.9 | 1.37 (1.21, 1.34) | Reference |  |  |
| Q2 | 1603/1140965.8 | 1.40 (1.34, 1.48) | 1.06 (0.99, 1.14) | 0.122 | 4.81% (1.47%, 8.54%) |
| Q3 | 1844/1131465.1 | 1.63 (1.56, 1.71) | 1.15 (1.07, 1.24) | <0.001 | 11.77% (8.40%, 15.23%) |
| Q4 | 2834/1093521.3 | 2.59 (2.50, 2.69) | 1.65 (1.55, 1.77) | <0.001 | 32.16% (29.22%, 35.00%) |

Model adjusted for age, sex, ethnicity, education level, employment status, BMI, smoking status, drinking status, physical activity, and Townsend deprivation index. Q1: Quartile 1; Q2: Quartile 2; Q3: Quartile 3; Q4: Quartile 4.

**Supplemental Table 27 Joint effects of biological age accelerations and PRS on the risk of VHD after excluding participants with VHD in the first 2 years**

| **Group** | **Cases/Person years** | | **HR (95%CI)** | ***P*** |
| --- | --- | --- | --- | --- |
| **Low genetic risk** | | | | |
| KDM-BA acceleration | Q1 | 206/199829.2 | Reference |  |
|  | Q2 | 208/194310.3 | 0.98 (0.81, 1.19) | 0.856 |
|  | Q3 | 261/188330.5 | 1.22 (1.01, 1.46) | 0.034 |
|  | Q4 | 368/175629.7 | 1.72 (1.45, 2.05) | <0.001 |
| Intermediate genetic risk | | | | |
| KDM-BA acceleration | Q1 | 703/594196.8 | 1.16 (0.99, 1.35) | 0.063 |
|  | Q2 | 855/579487.9 | 1.38 (1.18, 1.60) | <0.001 |
|  | Q3 | 1002/566551 | 1.57 (1.35, 1.83) | <0.001 |
|  | Q4 | 1392/528662.7 | 2.20 (1.89, 2.55) | <0.001 |
| High genetic risk | | | | |
| KDM-BA acceleration | Q1 | 307/190914.9 | 1.60 (1.34, 1.91) | <0.001 |
|  | Q2 | 365/194256.3 | 1.77 (1.49, 2.10) | <0.001 |
|  | Q3 | 424/189873 | 2.00 (1.69, 2.36) | <0.001 |
|  | Q4 | 584/178861.9 | 2.80 (2.38, 3.29) | <0.001 |
|  | | | | |
| Low genetic risk | | | | |
| PhenoAge acceleration | Q1 | 193/196977.5 | Reference |  |
|  | Q2 | 222/195625 | 1.10 (0.91, 1.33) | 0.337 |
|  | Q3 | 258/190949.1 | 1.22 (1.01, 1.47) | 0.040 |
|  | Q4 | 370/174548.1 | 1.69 (1.42, 2.01) | <0.001 |
| Intermediate genetic risk | | | | |
| PhenoAge acceleration | Q1 | 766/595516.8 | 1.32 (1.13, 1.55) | <0.001 |
|  | Q2 | 820/584588.3 | 1.37 (1.17, 1.61) | <0.001 |
|  | Q3 | 948/572352.2 | 1.52 (1.30, 1.77) | <0.001 |
|  | Q4 | 1418/516441.2 | 2.21 (1.89, 2.57) | <0.001 |
| High genetic risk | | | | |
| PhenoAge acceleration | Q1 | 340/198952.8 | 1.79 (1.50, 2.14) | <0.001 |
|  | Q2 | 360/195067.3 | 1.83 (1.53, 2.18) | <0.001 |
|  | Q3 | 397/189346.3 | 1.93 (1.62, 2.29) | <0.001 |
|  | Q4 | 583/170539.7 | 2.80 (2.37, 3.30) | <0.001 |

Model adjusted for age, sex, ethnicity, education level, employment status, BMI, smoking status, drinking status, physical activity, and Townsend deprivation index. Q1: Quartile 1; Q2: Quartile 2; Q3: Quartile 3; Q4: Quartile 4.

**Supplemental Table 28 Associations of biological age accelerations with incident VHD in different genetic risk backgrounds after excluding participants with VHD in the first 2 years**

| **Group** | **Cases/Person years** | | **HR (95%CI)** | ***P*** | |
| --- | --- | --- | --- | --- | --- |
| **Low genetic risk** | | | | | |
| KDM-BA acceleration (continue) | | 1043/758099.7 | 1.34 (1.26, 1.43) | | <0.001 |
| KDM-BA acceleration | Q1 | 206/199829.2 | Reference |  | |
|  | Q2 | 208/194310.3 | 1.00 (0.82, 1.21) | 0.970 | |
|  | Q3 | 261/188330.5 | 1.25 (1.04, 1.51) | 0.020 | |
|  | Q4 | 368/175629.7 | 1.80 (1.50, 2.17) | <0.001 | |
|  | | | | | |
| KDM-BA acceleration (continue) | | 4266/2268150.9 | 1.34 (1.30, 1.39) | | <0.001 |
| KDM-BA acceleration | Q1 | 780/588920.8 | Reference |  | |
|  | Q2 | 901/583359.9 | 1.11 (1.01, 1.22) | 0.035 | |
|  | Q3 | 1088/565299.5 | 1.32 (1.20, 1.45) | <0.001 | |
|  | Q4 | 1497/530570.7 | 1.84 (1.68, 2.02) | <0.001 | |
| **High genetic risk** | | | | | |
| KDM-BA acceleration (continue) | | 1405/756453.5 | 1.40 (1.33, 1.48) | | <0.001 |
| KDM-BA acceleration | Q1 | 250/199386.7 | Reference |  | |
|  | Q2 | 293/192464.8 | 1.16 (0.98, 1.38) | 0.081 | |
|  | Q3 | 328/187272.5 | 1.28 (1.08, 1.52) | 0.004 | |
|  | Q4 | 534/177329.6 | 2.08 (1.77, 2.44) | <0.001 | |
|  | | | | | |
| **Low genetic risk** | | | | | |
| PhenoAge acceleration (continue) | | 1043/758099.7 | 1.29 (1.21, 1.37) | | <0.001 |
| PhenoAge acceleration | Q1 | 193/196977.5 | Reference |  | |
|  | Q2 | 222/195625 | 1.11 (0.92, 1.35) | 0.272 | |
|  | Q3 | 258/190949.1 | 1.25 (1.03, 1.51) | 0.022 | |
|  | Q4 | 370/174548.1 | 1.77 (1.47, 2.12) | <0.001 | |
| **Intermediate genetic risk** | | | | | |
| PhenoAge acceleration (continue) | | 4266/2268150.9 | 1.29 (1.26, 1.33) | | <0.001 |
| PhenoAge acceleration | Q1 | 835/594479.2 | Reference |  | |
|  | Q2 | 884/585525.4 | 1.02 (0.93, 1.13) | 0.628 | |
|  | Q3 | 1028/572197.8 | 1.15 (1.04, 1.26) | 0.004 | |
|  | Q4 | 1519/515948.5 | 1.65 (1.51, 1.81) | <0.001 | |
| **High genetic risk** | | | | | |
| PhenoAge acceleration (continue) | | 1405/756453.5 | 1.34(1.27, 1.41) | <0.001 | |
| PhenoAge acceleration | Q1 | 252/197988.3 | Reference |  | |
|  | Q2 | 299/194275.8 | 1.14(0.96, 1.34) | 0.140 | |
|  | Q3 | 328/191560.3 | 1.17(0.99, 1.39) | 0.060 | |
|  | Q4 | 526/172629.2 | 1.82(1.56, 2.14) | <0.001 | |

Model adjusted for age, sex, ethnicity, education level, employment status, BMI, smoking status, drinking status, physical activity, and Townsend deprivation index. Q1: Quartile 1; Q2: Quartile 2; Q3: Quartile 3; Q4: Quartile 4.

**Supplemental Table 29 Additive interaction between biological age acceleration and genetic risk categories on incident VHD after excluding participants with VHD in the first 2 years**

|  | | **Intermediate genetic risk** | |  | **High genetic risk** | |
| --- | --- | --- | --- | --- | --- | --- |
|  |  | **RERI (95%CI)** | **AP (95%CI)** |  | **RERI (95%CI)** | **AP (95%CI)** |
| KDM-BA acceleration (Quartiles) | Q2 | 0.23 (0.02, 0.45) | 0.17 (0.01, 0.33) |  | 0.19 (-0.12, 0.49) | 0.11 (-0.07, 0.28) |
|  | Q3 | 0.19 (-0.03, 0.42) | 0.12 (-0.03, 0.27) |  | 0.18 (-0.14, 0.50) | 0.09 (-0.07, 0.25) |
|  | Q4 | 0.31 (0.07, 0.56） | 0.14 (0.03, 0.26) |  | 0.48 (0.13, 0.82) | 0.17 (0.05, 0.29) |
|  |  |  |  |  |  |  |
| PhenoAge acceleration (Quartiles) | Q2 | -0.05 (-0.29, 0.20) | -0.03 (-0.21, 0.14) |  | -0.07 (-0.41, 0.27) | -0.04 (-0.22, 0.15) |
|  | Q3 | -0.02 (-0.27, 0.23) | -0.01 (-0.28, 0.15) |  | -0.08 (-0.42, 0.26) | -0.04 (-0.22, 0.14) |
|  | Q4 | 0.19 (-0.06, 0.45) | 0.09 (-0.03, 0.21) |  | 0.31 (0.04, 0.67) | 0.11 (-0.02, 0.24) |

Model adjusted for age, sex, ethnicity, education level, employment status, BMI, smoking status, drinking status, physical activity, and Townsend deprivation index. Q1: Quartile 1; Q2: Quartile 2; Q3: Quartile 3; Q4: Quartile 4.

**Supplemental Table 30 Associations of biological ages with incident VHD diagnosed by a broad definition.**

| **Biological age** | **Cases/**  **Person years** | **Incidence rate (95%CI), per 1000 person-years** | **HR (95%CI)** | ***P*** | **PAF (95%CI)** |
| --- | --- | --- | --- | --- | --- |
| KDM-BA acceleration (Continuous) | 3538/4539478.3 | 0.78 (0.75, 0.81) | 1.48 (1.43, 1.53) | <0.001 |  |
| KDM-BA acceleration (Quartiles) | | | | | |
| Q1 | 462/1148301.7 | 0.40 (0.37, 0.44) | Reference |  |  |
| Q2 | 690/1142817.3 | 0.60 (0.56, 0.65) | 1.37 (1.22, 1.54) | <0.001 | 19.79% (14.19%, 25.19%) |
| Q3 | 876/1136258.5 | 0.77 (0.72, 0.82) | 1.63 (1.46, 1.83) | <0.001 | 30.94% (25.60%, 35.91%) |
| Q4 | 1510/1112100.9 | 1.36 (1.29, 1.43) | 2.62 (2.34, 2.92) | <0.001 | 53.14% (49.56%, 56.78%) |
|  |  |  |  |  |  |
| PhenoAge acceleration (Continuous) | 3538/4539478.3 | 0.78 (0.75, 0.81) | 1.33 (1.29, 1.37) | <0.001 |  |
| PhenoAge acceleration (Quartiles) | | | | | |
| Q1 | 552/1152306.4 | 0.48 (0.44, 0.52) | Reference |  |  |
| Q2 | 684/1147245.6 | 0.60 (0.55, 0.64) | 1.14 (1.02, 1.28) | 0.021 | 10.68% (5.13%, 16.30%) |
| Q3 | 866/1138095.3 | 0.76 (0.71, 0.81) | 1.31 (1.18, 1.46) | <0.001 | 22.14% (17.22%, 27.00%) |
| Q4 | 1436/1101831.1 | 1.30 (1.24, 1.37) | 1.89 (1.71, 2.10) | <0.001 | 44.47% (40.63%,48.37%) |

Model adjusted for age, sex, ethnicity, education level, employment status, BMI, smoking status, drinking status, physical activity, and Townsend deprivation index. Q1: Quartile 1; Q2: Quartile 2; Q3: Quartile 3; Q4: Quartile 4.

**Supplemental Table 31 Joint effects of biological age accelerations and PRS on the risk of VHD diagnosed by a broad definition.**

| **Group** | **Cases/Person years** | | **HR (95%CI)** | ***P*** |
| --- | --- | --- | --- | --- |
| **Low genetic risk** | | | | |
| KDM-BA acceleration | Q1 | 47/200969.8 | Reference |  |
|  | Q2 | 69/195294.5 | 1.37 (0.94, 1.98) | 0.097 |
|  | Q3 | 80/189442.9 | 1.51 (1.05, 2.17) | 0.025 |
|  | Q4 | 177/176875.3 | 3.14 (2.27, 4.35) | <0.001 |
| **Intermediate genetic risk** | | | | |
| KDM-BA acceleration | Q1 | 237/597606 | 1.72 (1.26, 2.36) | <0.001 |
|  | Q2 | 346/582673.4 | 2.34 (1.73, 3.18) | <0.001 |
|  | Q3 | 467/570276.1 | 2.98 (2.20,4.02) | <0.001 |
|  | 733 | 733/532489.4 | 4.41 (3.27, 5.93) | <0.001 |
| **High genetic risk** | | | | |
| KDM-BA acceleration | Q1 | 142/191932 | 3.28 (2.36, 4.56) | <0.001 |
|  | Q2 | 211/195292.8 | 4.32 (3.15, 5.92) | <0.001 |
|  | 236 | 236/191072.3 | 4.51 (3.30, 6.18) | <0.001 |
|  | Q4 | 368/180092.3 | 6.78 (4.99, 9.21) | <0.001 |
|  | | | | |
| **Low genetic risk** | | | | |
| PhenoAge acceleration | Q1 | 60/198040.5 | Reference |  |
|  | Q2 | 66/196760.1 | 1.00 (0.71, 1.42) | 0.996 |
|  | Q3 | 80/192069.2 | 1.10 (0.79, 1.54) | 0.571 |
|  | Q4 | 167/175712.8 | 2.08 (1.54, 2.79) | <0.001 |
| **Intermediate genetic risk** | | | | |
| PhenoAge acceleration | Q1 | 287/598896.8 | 1.59 (1.21, 2.11) | 0.001 |
|  | Q2 | 248/587869.9 | 1.80 (1.37, 2.37) | <0.001 |
|  | Q3 | 435/575726.3 | 2.06 (1.57, 2.70) | <0.001 |
|  | Q4 | 713/520551.9 | 3.01 (2.31, 3.92) | <0.001 |
| **High genetic risk** | | | | |
| PhenoAge acceleration | Q1 | 160/200046.9 | 2.74 (2.04, 3.69) | <0.001 |
|  | Q2 | 198/196078.5 | 3.11 (2.33, 4.15) | <0.001 |
|  | Q3 | 246/190401.7 | 3.54 (2.67, 4.70) | <0.001 |
|  | Q4 | 353/171862.3 | 4.60 (3.49, 6.06) | <0.001 |

Model adjusted for age, sex, ethnicity, education level, employment status, BMI, smoking status, drinking status, physical activity, and Townsend deprivation index. Q1: Quartile 1; Q2: Quartile 2; Q3: Quartile 3; Q4: Quartile 4.

**Supplemental Table 32 Associations of biological age accelerations with incident VHD diagnosed by a broad definition in different genetic risk backgrounds**

| **Group** | **Cases/Person years** | | **HR (95%CI)** | ***P*** | |
| --- | --- | --- | --- | --- | --- |
| **Low genetic risk** | | | | | |
| KDM-BA acceleration (continue) | | 373/762582.5 | 1.65 (1.50, 1.81) | | <0.001 |
| KDM-BA acceleration | Q1 | 47/200969.8 | Reference |  | |
|  | Q2 | 69/195294.5 | 1.39 (0.95, 2.01) | 0.0860 | |
|  | Q3 | 80/189442.9 | 1.55 (1.08, 2.24) | 0.019 | |
|  | Q4 | 177/176875.3 | 3.29 (2.34, 4.63) | <0.001 | |
|  | | | | | |
| KDM-BA acceleration (continue) | | 1783/2283044.9 | 1.44 (1.38, 1.51) | | <0.001 |
| KDM-BA acceleration | Q1 | 237/597606 | Reference |  | |
|  | Q2 | 346/582673.4 | 1.34 (1.14, 1.58) | <0.001 | |
|  | Q3 | 467/570276.1 | 1.68 (1.43, 1.97) | <0.001 | |
|  | Q4 | 733/532489.4 | 2.44 (2.09, 2.86) | <0.001 | |
| **High genetic risk** | | | | | |
| KDM-BA acceleration (continue) | | 957/758389.4 | 1.39 (1.30, 1.48) | | <0.001 |
| KDM-BA acceleration | Q1 | 142/191932 | Reference |  | |
|  | Q2 | 211/195292.8 | 1.34 (1.09, 1.66) | 0.007 | |
|  | Q3 | 236/191072.3 | 1.43 (1.16, 1.77) | <0.001 | |
|  | Q4 | 368/180092.3 | 2.20 (1.79, 2.71) | <0.001 | |
|  | | | | | |
| **Low genetic risk** | | | | | |
| PhenoAge acceleration (continue) | | 373/762582.5 | 1.40 (1.27, 1.54) | | <0.001 |
| PhenoAge acceleration | Q1 | 60/198040.5 | Reference |  | |
|  | Q2 | 66/196760.1 | 1.00 (0.71,1.43) | 0.981 | |
|  | Q3 | 80/192069.2 | 1.12 (0.80, 1.57) | 0.520 | |
|  | Q4 | 167/175712.8 | 2.13 (1.56, 2.91) | <0.001 | |
| **Intermediate genetic risk** | | | | | |
| PhenoAge acceleration (continue) | | 1783/2283044.9 | 1.33 (1.27, 1.39) | | <0.001 |
| PhenoAge acceleration | Q1 | 287/598896.8 | Reference |  | |
|  | Q2 | 348587869.9 | 1.12 (0.96, 1.31) | 0.159 | |
|  | Q3 | 435/575726.3 | 1.27 (1.09, 1.47) | 0.002 | |
|  | Q4 | 713/520551.9 | 1.82 (1.57, 2.10) | <0.001 | |
| **High genetic risk** | | | | | |
| PhenoAge acceleration (continue) | | 957/758389.4 | 1.27 (1.20, 1.36) | <0.001 | |
| PhenoAge acceleration | Q1 | 160/200046.9 | Reference |  | |
|  | Q2 | 198/196078.5 | 1.15 (0.94, 1.42) | 0.177 | |
|  | Q3 | 246/190401.7 | 1.17 (1.09, 1.63) | 0.005 | |
|  | Q4 | 353/171862.3 | 1.82 (1.46, 2.17) | <0.001 | |

Model adjusted for age, sex, ethnicity, education level, employment status, BMI, smoking status, drinking status, physical activity, and Townsend deprivation index. Q1: Quartile 1; Q2: Quartile 2; Q3: Quartile 3; Q4: Quartile 4.

**Supplemental Table 33 Additive interaction between biological age acceleration and genetic risk categories on incident VHD diagnosed by a broad definition.**

|  | | **Intermediate genetic risk** | |  | **High genetic risk** | |
| --- | --- | --- | --- | --- | --- | --- |
|  |  | **RERI (95%CI)** | **AP (95%CI)** |  | **RERI (95%CI)** | **AP (95%CI)** |
| KDM-BA acceleration (Quartiles) | Q2 | 0.25 (-0.26, 0.76) | 0.11 (-0.12, 0.33) |  | 0.67 (-0.19, 1.53) | 0.16 (-0.04,0.35) |
|  | Q3 | 0.74 (0.26, 1.23) | 0.25 (0.07, 0.43) |  | 0.73 (-0.14, 1.59) | 0.16 (-0.03,0.35) |
|  | Q4 | 0.54 (-0.08, 1.16） | 0.12 (-0.03, 0.27) |  | 1.36 (0.35, 2.37) | 0.20 (0.06, 0.34) |
|  |  |  |  |  |  |  |
| PhenoAge acceleration (Quartiles) | Q2 | 0.21 (-0.20, 0.62) | 0.12 (-0.12, 0.35) |  | 0.36 (-0.31, 1.04) | 0.12 (-0.10, 0.33) |
|  | Q3 | 0.37 (-0.03, 0.76) | 0.18 (-0.03, 0.38) |  | 0.70 (0.04, 1.36) | 0.20 (0.01, 0.38) |
|  | Q4 | 0.34 (-0.12, 0.80) | 0.11 (-0.05, 0.27) |  | 0.78 (0.06, 1.50) | 0.17 (0.02, 0.32) |

RERI: relative excess risk of interaction; AP: attributable proportion. RERI and AP were calculated by “Delta” method. Q1: Quartile 1; Q2: Quartile 2; Q3: Quartile 3; Q4: Quartile 4

**Supplemental Table 34 Associations of biological ages with incident VHD by additionally controlling for comorbidities**

| **Biological age** | **Cases/**  **Person years** | **Incidence rate (95%CI), per 1000 person-years** | **HR (95%CI)** | ***P*** | **PAF** |
| --- | --- | --- | --- | --- | --- |
| KDM-BA acceleration (Continuous) | 8146/4512447.4 | 1.81 (1.77, 1.84) | 1.13 (1.11, 1.16) | <0.001 |  |
| KDM-BA acceleration (Quartiles) | | | | | |
| Q1 | 1445/1142018.4 | 1.27 (1.20, 1.33) | Reference |  |  |
| Q2 | 1702/1136599.6 | 1.50 (1.43, 1.57) | 01.02 (0.95, 1.10) | 0.514 | 8.17% (4.84%, 11.49%) |
| Q3 | 2038/1129286.9 | 1.80 (1.73, 1.88) | 1.08 (1.01, 1.16) | 0.023 | 17.03% (13.80%, 20.41%) |
| Q4 | 2961/1104542.5 | 2.68 (2.59, 2.78) | 1.25 (1.17, 1.34) | <0.001 | 34.41% (31.78%, 37.04%) |
|  |  |  |  |  |  |
| PhenoAge acceleration (Continuous) | 8146/4512447.4 | 1.81 (1.77, 1.84) | 1.07 (1.05, 1.09) | <0.001 |  |
| PhenoAge acceleration (Quartiles) | | | | | |
| Q1 | 1524/1146118.2 | 1.33 (1.26, 1.40) | Reference |  |  |
| Q2 | 1683/1141056.3 | 1.47 (1.41, 1.55) | 0.97 (0.90, 1.04) | 0.371 | 4.96% (1.79%, 8.43%) |
| Q3 | 1932/1131566.5 | 1.71 (1.63, 1.79) | 0.95 (0.89, 1.02) | 0.151 | 11.81% (8,46%, 15.25%) |
| Q4 | 3007/1093706.4 | 2.75 (2.65, 2.85) | 1.09 (1.02, 1.17) | 0.012 | 32.73% (30.91%, 35.58%) |

Model adjusted for age, sex, ethnicity, education level, employment status, BMI, smoking status, drinking status, physical activity, Townsend deprivation index, hypertension, stroke, atrial fibrillation, chronic kidney disease, cancer and type 2 diabetes. Q1: Quartile 1; Q2: Quartile 2; Q3: Quartile 3; Q4: Quartile 4.

**Supplemental Table 35 Joint effects of biological age accelerations and PRS on the risk of VHD by additionally controlling for comorbidities**

| **Group** | **Cases/Person years** | | **HR (95%CI)** | ***P*** |  |
| --- | --- | --- | --- | --- | --- |
| **Low genetic risk** | | | | |  |
| KDM-BA acceleration | Q1 | 211/199863.7 | Reference |  |  |
|  | Q2 | 219/194381.2 | 0.89 (0.73, 1.07) | 0.209 |  |
|  | Q3 | 268/188392.6 | 1.02 (0.85, 1.22) | 0.818 |  |
|  | Q4 | 388/175719.4 | 1.22 (1.03, 1.45) | 0.020 |  |
| Intermediate genetic risk | | | | |  |
| KDM-BA acceleration | Q1 | 743/594299.7 | 1.17 (1.00, 1.36) | 0.044 |  |
|  | Q2 | 894/579512.9 | 1.25 (1.08, 1.46) | 0.003 |  |
|  | Q3 | 1059/566632.3 | 1.31 (1.13, 1.52) | <0.001 |  |
|  | Q4 | 1468/528759.8 | 1.50 (1.29, 1.73) | <0.001 |  |
| High genetic risk | | | | |  |
| KDM-BA acceleration | Q1 | 320/190847.6 | 1.53 (1.28, 1.82) | <0.001 |  |
|  | Q2 | 384/194241.1 | 1.59 (1.34, 1.88) | <0.001 |  |
|  | Q3 | 451/189826.4 | 1.65 (1.40, 1.94) | <0.001 |  |
|  | Q4 | 621/178824.4 | 1.88 (1.60, 2.20) | <0.001 |  |
|  | | | | |  |
| Low genetic risk | | | | |  |
| PhenoAge acceleration | Q1 | 198/197055.1 | Reference |  |  |
|  | Q2 | 232/195665.3 | 1.03(0.85, 1.25) | 0.759 |  |
|  | Q3 | 271/191019 | 1.03(0.86, 1.24) | 0.737 |  |
|  | Q4 | 385/174617.5 | 1.14(0.96, 1.36) | 0.133 |  |
| Intermediate genetic risk | | | | |  |
| PhenoAge acceleration | Q1 | 803/595588.8 | 1.34(1.15, 1.56) | <0.001 |  |
|  | Q2 | 867/584667.6 | 1.28(1.09, 1.49) | 0.002 |  |
|  | Q3 | 986/572355.2 | 1.25(1.07, 1.45) | 0.005 |  |
|  | Q4 | 1508/516593.1 | 1.48(1.27, 1.72) | <0.001 |  |
| High genetic risk | | | | |  |
| PhenoAge acceleration | Q1 | 357/198880.8 | 1.74(1.46, 2.07) | <0.001 |  |
|  | Q2 | 375/195030 | 1.63(1.37, 1.94) | <0.001 |  |
|  | Q3 | 421/189359.5 | 1.59(1.34, 1.88) | <0.001 |  |
|  | Q4 | 623/170469.3 | 1.83(1.55, 2.15) | <0.001 |  |

Model adjusted for age, sex, ethnicity, education level, employment status, BMI, smoking status, drinking status, physical activity, Townsend deprivation index, hypertension, stroke, atrial fibrillation, chronic kidney disease, cancer and type 2 diabetes. Q1: Quartile 1; Q2: Quartile 2; Q3: Quartile 3; Q4: Quartile 4.

**Supplemental Table 36 Associations of biological age accelerations with incident VHD in different genetic risk backgrounds by additionally controlling for comorbidities**

| **Group** | **Cases/Person years** | | **HR (95%CI)** | ***P*** | |
| --- | --- | --- | --- | --- | --- |
| **Low genetic risk** | | | | | |
| KDM-BA acceleration (continue) | | 1086/758356.8 | 1.15 (1.09, 1.23) | | <0.001 |
| KDM-BA acceleration | Q1 | 211/199863.7 | Reference |  | |
|  | Q2 | 219/194381.2 | 0.90 (0.74, 1.09) | 0.277 | |
|  | Q3 | 268/188392.6 | 1.06 (0.88, 1.28) | 0.512 | |
|  | Q4 | 388/175719.4 | 1.31 (1.09, 1.58) | 0.005 | |
| **Intermediate genetic risk** | | | | | |
| KDM-BA acceleration (continue) | | 4164/2269204.7 | 1.12 (1.09, 1.16) | | <0.001 |
| KDM-BA acceleration | Q1 | 743/594299.7 | Reference |  | |
|  | Q2 | 894/579512.9 | 1.06 (0.96, 1.17) | 0.270 | |
|  | Q3 | 1059/566632.3 | 1.09 (0.99, 1.20) | 0.073 | |
|  | Q4 | 1468/528759.8 | 1.22 (1.11, 1.35) | <0.001 | |
| **High genetic risk** | | | | | |
| KDM-BA acceleration (continue) | | 1776/753739.5 | 1.14 (1.09, 1.2) | | <0.001 |
| KDM-BA acceleration | Q1 | 320/190847.6 | Reference |  | |
|  | Q2 | 384/194241.1 | 1.05 (0.90, 1.22) | 0.525 | |
|  | Q3 | 451/189826.4 | 1.11 (0.96, 1.28) | 0.176 | |
|  | Q4 | 621/178824.4 | 1.31 (1.13, 1.51) | <0.001 | |
|  | | | | | |
| **Low genetic risk** | | | | | |
| PhenoAge acceleration (continue) | | 1086/758356.8 | 1.09 (1.02, 1.16) | | 0.008 |
| PhenoAge acceleration | Q1 | 198/197055.1 | Reference |  | |
|  | Q2 | 232/195665.3 | 1.05 (0.87, 1.27) | 0.615 | |
|  | Q3 | 271/191019 | 1.06 (0.88, 1.28) | 0.541 | |
|  | Q4 | 385/174617.5 | 1.19 (0.99, 1.43) | 0.063 | |
| **Intermediate genetic risk** | | | | | |
| PhenoAge acceleration (continue) | | 4164/2269204.7 | 1.07 (1.04, 1.11) | | <0.001 |
| PhenoAge acceleration | Q1 | 803/595588.8 | Reference |  | |
|  | Q2 | 867/584667.6 | 0.94 (0.86, 1.04) | 0.236 | |
|  | Q3 | 986/572355.2 | 0.91 (0.83 – 1.00) | 0.061 | |
|  | Q4 | 1508/516593.1 | 1.06 (0.97, 1.17) | 0.204 | |
| **High genetic risk** | | | | | |
| PhenoAge acceleration (continue) | | 1776/753739.5 | 1.07(1.02, 1.12) | | 0.006 |
| PhenoAge acceleration | Q1 | 357/198880.8 | Reference |  | |
|  | Q2 | 375/195030 | 0.95 (0.82, 1.10) | 0.501 | |
|  | Q3 | 421/189359.5 | 0.94 (0.81, 1.08) | 0.368 | |
|  | Q4 | 623/170469.3 | 1.12 (0.97, 1.29) | 0.126 | |

Model adjusted for age, sex, ethnicity, education level, employment status, BMI, smoking status, drinking status, physical activity, Townsend deprivation index, hypertension, stroke, atrial fibrillation, chronic kidney disease, cancer and type 2 diabetes. Q1: Quartile 1; Q2: Quartile 2; Q3: Quartile 3; Q4: Quartile 4.

**Supplemental Table 37 Additive interaction between biological age acceleration and genetic risk categories on incident VHD by additionally controlling for comorbidities**

|  | | **Intermediate genetic risk** | |  | **High genetic risk** | |
| --- | --- | --- | --- | --- | --- | --- |
|  |  | **RERI (95%CI)** | **AP (95%CI)** |  | **RERI (95%CI)** | **AP (95%CI)** |
| KDM-BA acceleration (Quartiles) | Q2 | 0.20 (0.00, 0.39) | 0.16 (-0.01, 0.32) |  | 0.17 (-0.11, 0.45) | 0.11 (-0.07, 0.29) |
|  | Q3 | 0.12 (-0.09, 0.32) | 0.09 (-0.07, 0.25) |  | 0.10 (-0.19, 0.38) | 0.06 (-0.11, 0.23) |
|  | Q4 | 0.10 (-0.10, 0.31） | 0.07 (-0.07, 0.21) |  | 0.13 (-0.15, 0.41) | 0.07 (-0.08, 0.22) |
|  |  |  |  |  |  |  |
| PhenoAge acceleration (Quartiles) | Q2 | -0.09 (-0.33, 0.14) | -0.07 (-0.25, 0.11) |  | -0.13(-0.45, 0.19) | -0.08 (-0.28, 0.11) |
|  | Q3 | -0.12 (-0.36, 0.11) | -0.10 (-0.28, 0.08) |  | -0.18 (-0.49, 0.13) | -0.11 (-0.31, 0.08) |
|  | Q4 | -0.01 (-0.22, 0.21) | 0.00 (-0.15, 0.14) |  | -0.05 (-0.35, 0.25) | -0.03 (-0.19, 0.13) |

Model adjusted for age, sex, ethnicity, education level, employment status, BMI, smoking status, drinking status, physical activity, Townsend deprivation index, hypertension, stroke, atrial fibrillation, chronic kidney disease, cancer and type 2 diabetes. Q1: Quartile 1; Q2: Quartile 2; Q3: Quartile 3; Q4: Quartile 4.

**Supplemental Table 38 Associations of biological ages with incident VHD in biologically younger and older groups**

| **Biological age** | **Cases/Person years** | **Incidence rate (95%CI), per 1000 person-years** | **HR (95%CI)** | ***P*** | **PAF (95%CI)** |
| --- | --- | --- | --- | --- | --- |
| KDM-BA acceleration (Quartiles) |  |  |  |  |  |
| Biologically younger | 3354/2398538.7 | 1.40 (1.35, 1.45) | Reference |  |  |
| Biologically older | 4792/2113908.8 | 2.27 (2.20, 2.33) | 1.46 (1.39, 1.53) | <0.001 | 21.79% (19.73%, 23.71%) |
|  |  |  |  |  |  |
| PhenoAge acceleration (Quartiles) |  |  |  |  |  |
| Biologically younger | 3436/2442382.2 | 1.41 (1.36, 1.45) | Reference |  |  |
| Biologically older | 4710/2070065.3 | 2.28 (2.21, 2.34) | 1.39 (1.33, 1.45) | <0.001 | 21.03% (19.10%, 22.99%) |

Model adjusted for age, sex, ethnicity, education level, employment status, BMI, smoking status, drinking status, physical activity, and Townsend deprivation index.

**Supplemental Table 39** **Joint effects of biological age accelerations and PRS on the risk of VHD in biologically younger and older groups**

| **Group** | **Cases/Person years** | | **HR (95%CI)** | ***P*** |
| --- | --- | --- | --- | --- |
| **Low genetic risk** | | | | |
| KDM-BA acceleration | Biologically younger | 455/415023.9 | Reference |  |
|  | Biologically older | 631/343332.9 | 1.48 (1.31, 1.67) | <0.001 |
| **Intermediate genetic risk** | | | | |
| KDM-BA acceleration | Biologically younger | 1746/1234068.5 | 1.30 (1.18, 1.45) | <0.001 |
|  | Biologically older | 2418/1035136.2 | 1.91 (1.72, 2.11) | <0.001 |
| **High genetic risk** | | | | |
| KDM-BA acceleration | Biologically younger | 750/405491.4 | 1.72 (1.53, 1.93) | <0.001 |
|  | Biologically older | 1026/348248.1 | 2.45 (2.19, 2.74) | <0.001 |
|  | | | | |
| **Low genetic risk** | | | | |
| PhenoAge acceleration | Biologically younger | 465/418372.1 | Reference |  |
|  | Biologically older | 621/339984.8 | 1.38 (1.22, 1.56) | 0.163 |
| **Intermediate genetic risk** | | | | |
| PhenoAge acceleration | Biologically younger | 1787/1259948.8 | 1.29 (1.16, 1.43) | <0.001 |
|  | Biologically older | 2377/1009255.8 | 1.80 (1.63, 1.99) | <0.001 |
| **High genetic risk** | | | | |
| PhenoAge acceleration | Biologically younger | 776/420220 | 1.70 (1.52, 1.91) | <0.001 |
|  | Biologically older | 1000/333519.5 | 2.32 (2.07, 2.59) | <0.001 |

Model adjusted for age, sex, ethnicity, education level, employment status, BMI, smoking status, drinking status, physical activity, and Townsend deprivation index.

**Supplemental Table 40 Associations of biological age accelerations with incident VHD in different genetic risk backgrounds in biologically younger and older groups**

| **Group** |  |  | **HR (95%CI)** | ***P*** |
| --- | --- | --- | --- | --- |
| **Low genetic risk** | | | | |
| KDM-BA acceleration | Biologically younger | 455/415023.9 | Reference |  |
|  | Biologically older | 631/343332.9 | 1.51(1.33, 1.72) | <0.001 |
| **Intermediate genetic risk** | | | | |
| KDM-BA acceleration | Biologically younger | 1746/1234068.5 | Reference |  |
|  | Biologically older | 2418/1035136.2 | 1.45(1.36, 1.55) | <0.001 |
| **High genetic risk** | | | | |
| KDM-BA acceleration | Biologically younger | 750/405491.4 | Reference |  |
|  | Biologically older | 1026/348248.1 | 1.44(1.3, 1.59) | <0.001 |
|  | | | | |
| **Low genetic risk** | | | | |
| PhenoAge acceleration | Biologically younger | 465/418372.1 | Reference |  |
|  | Biologically older | 621/339984.8 | 1.41(1.24, 1.6) | <0.001 |
| **Intermediate genetic risk** | | | | |
| PhenoAge acceleration | Biologically younger | 1787/1259948.8 | Reference |  |
|  | Biologically older | 2377/1009255.8 | 1.39(1.3, 1.48) | <0.001 |
| **High genetic risk** | | | | |
| PhenoAge acceleration | Biologically younger | 776/420220 | Reference |  |
|  | Biologically older | 1000/333519.5 | 1.36(1.23, 1.5) | <0.001 |

Model adjusted for age, sex, ethnicity, education level, employment status, BMI, smoking status, drinking status, physical activity, and Townsend deprivation index.

**Supplemental Table 41 Additive interaction between biological age acceleration and genetic risk categories on incident VHD in biologically younger and older groups**

|  | | **Intermediate genetic risk** | |  | **High genetic risk** | |
| --- | --- | --- | --- | --- | --- | --- |
|  |  | **RERI (95%CI)** | **AP (95%CI)** |  | **RERI (95%CI)** | **AP (95%CI)** |
| KDM-BA acceleration  (Quartiles) | Biologically older | 0.12 (-0.05, 0.30) | 0.07 (-0.03, 0.16) |  | 0.25 (0.02, 0.49) | 0.10 (0.01, 0.20) |
|  |  |  |  |  |  |  |
| PhenoAge-BA acceleration (Quartiles) | Biologically older | 0.13 (-0.03, 0.30) | 0.07 (-0.02, 0.17) |  | 0.23 (0.00, 0.46) | 0.10 (0.00, 0.20) |

Model adjusted for age, sex, ethnicity, education level, employment status, BMI, smoking status, drinking status, physical activity, and Townsend deprivation index.

342,886 participants with complete data on biological age algorithms and VHD

341,894 participants were included for data analysis

Exclude 160,485 participants without trait data for biological age algorithms or VHD

A total of 503,371 participants in the UK Biobank from 2006 to 2010

Individuals were excluded due to:

1. Refuse to participate (n=0)
2. Participants with VHD at baseline (n=992)

341,460 participants were included for data analysis

Further excluded participants with congenital valvular heart disease (n=434)

**Supplemental Figure 1 Flowchart of participant selection**

**
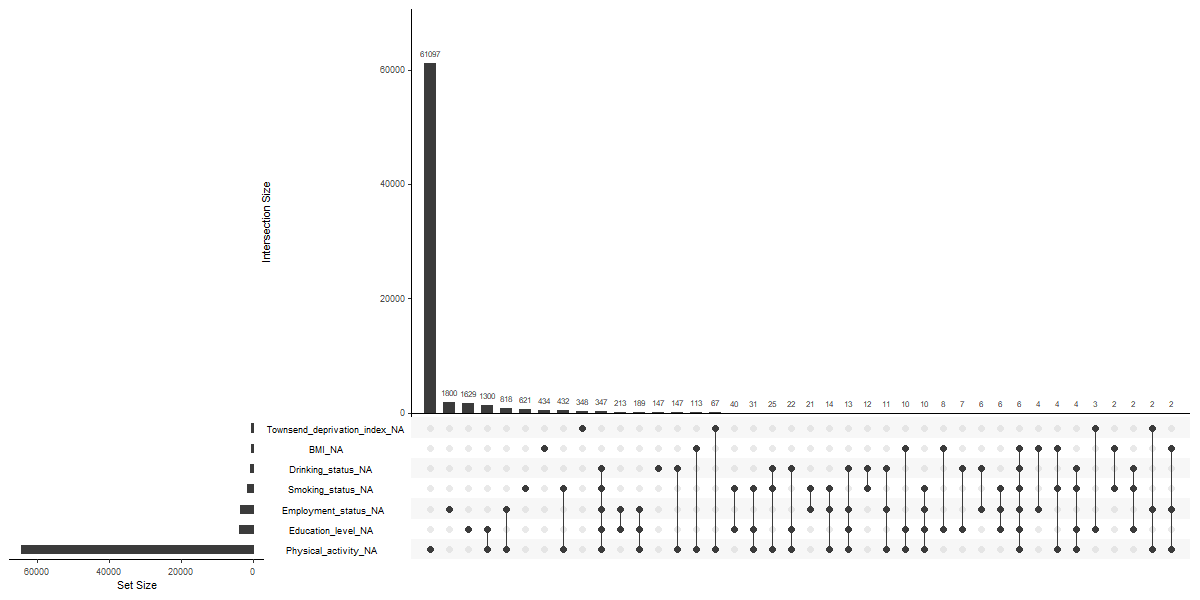
**

**Supplemental Figure 2 UpSet plot showing the missing data patterns**


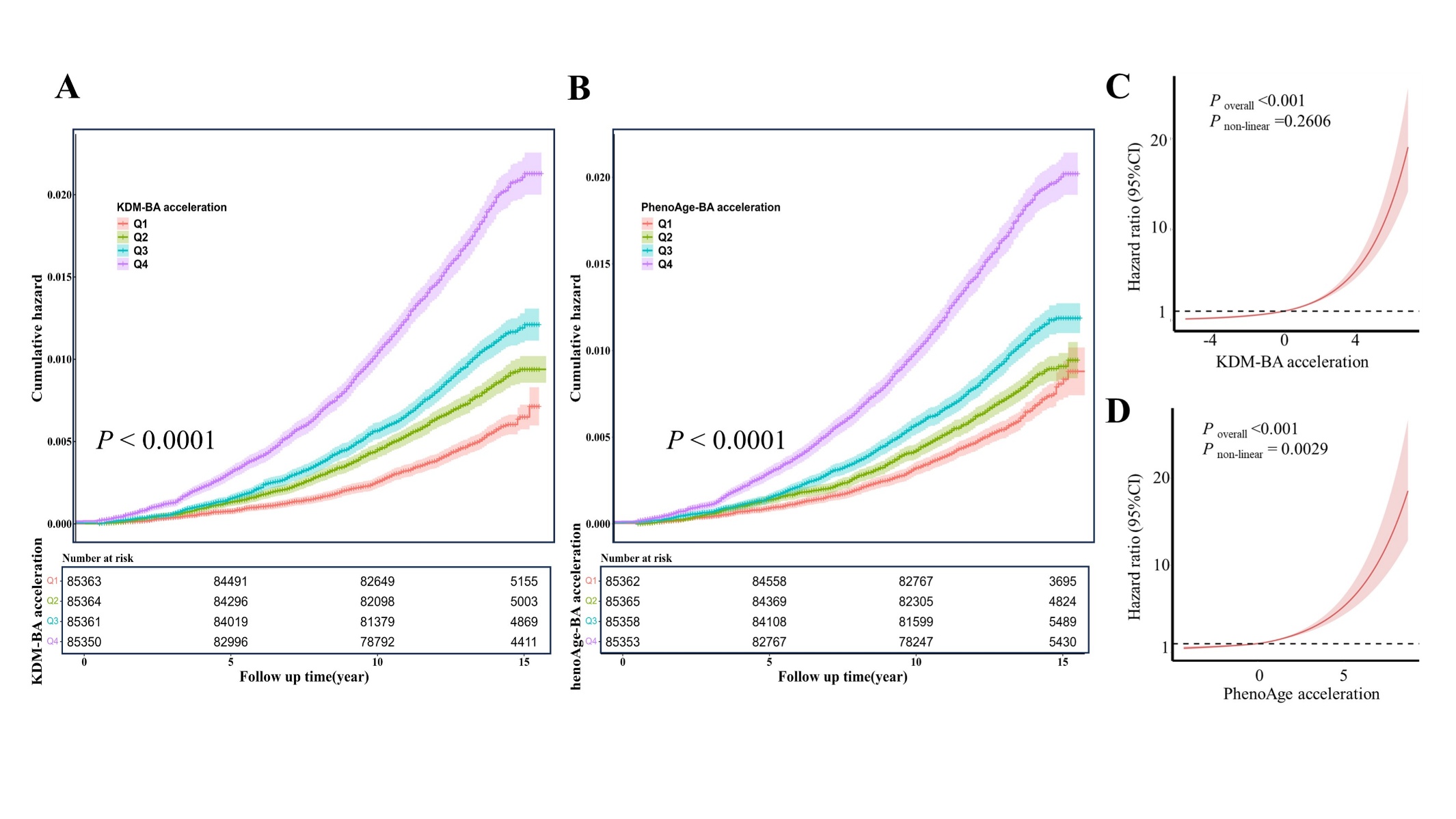


**Supplemental Figure 3 Association of biological aging with the risk AS.** (A) Cumulative AS Incidence Difference Among KDM-BA acceleration Groups. (B) Cumulative AS Incidence Difference Among PhenoAge acceleration Groups. (C) Association between KDM-BA acceleration and the risk of AS using RCS. (D) Association between PhenoAge acceleration and the risk of AS using RCS. All models adjusted for age, sex, ethnicity, education level, employment status, BMI, smoking status, drinking status, physical activity, and Townsend deprivation index. KDM-BA: Klemera–Doubal method biological age; Q1: Quartile 1; Q2: Quartile 2; Q3: Quartile 3; Q4: Quartile.


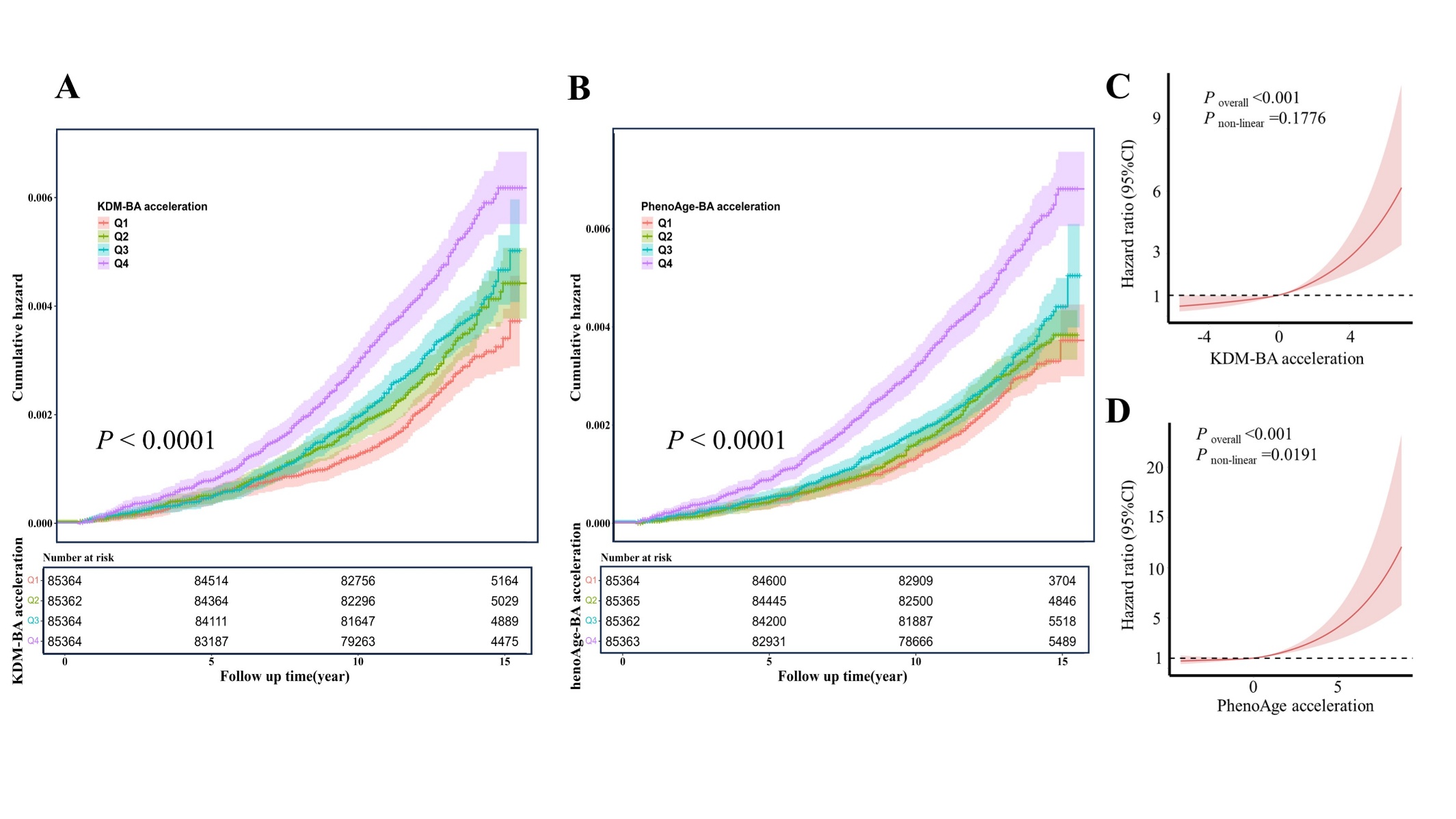


**Supplemental Figure 4 Association of biological aging with the risk AR.** (A) Cumulative AR Incidence Difference Among KDM-BA acceleration Groups. (B) Cumulative AR Incidence Difference Among PhenoAge acceleration Groups. (C) Association between KDM-BA acceleration and the risk of AR using RCS. (D) Association between PhenoAge acceleration and the risk of AR using RCS. All models adjusted for age, sex, ethnicity, education level, employment status, BMI, smoking status, drinking status, physical activity, and Townsend deprivation index. KDM-BA: Klemera–Doubal method biological age; Q1: Quartile 1; Q2: Quartile 2; Q3: Quartile 3; Q4: Quartile.


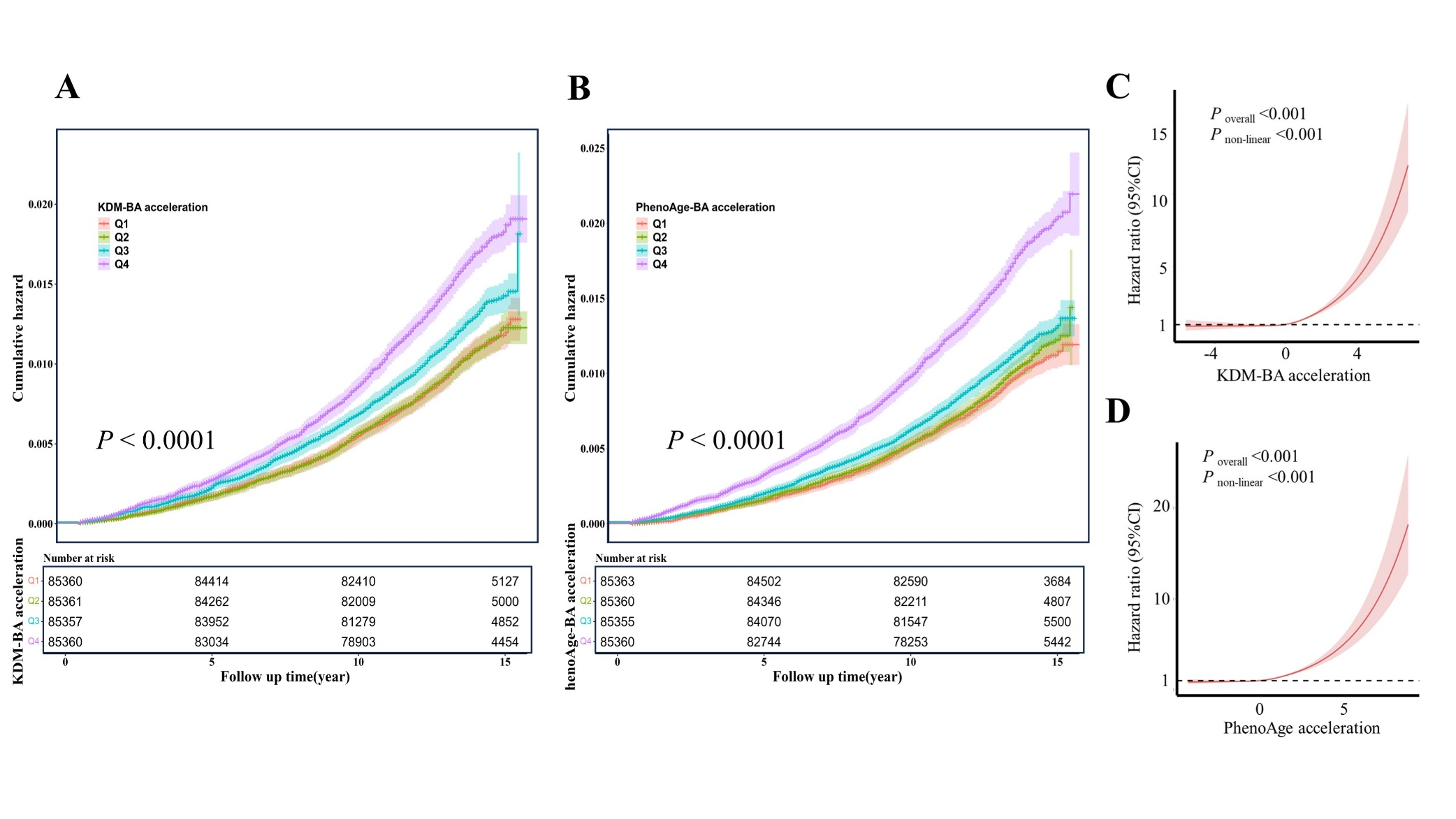


**Supplemental Figure 5 Association of biological aging with the risk MR.** (A) Cumulative MR Incidence Difference Among KDM-BA acceleration Groups. (B) Cumulative MR Incidence Difference Among PhenoAge acceleration Groups. (C) Association between KDM-BA acceleration and the risk of MR using RCS. (D) Association between PhenoAge acceleration and the risk of MR using RCS. All models adjusted for age, sex, ethnicity, education level, employment status, BMI, smoking status, drinking status, physical activity, and Townsend deprivation index. KDM-BA: Klemera–Doubal method biological age; Q1: Quartile 1; Q2: Quartile 2; Q3: Quartile 3; Q4: Quartile.


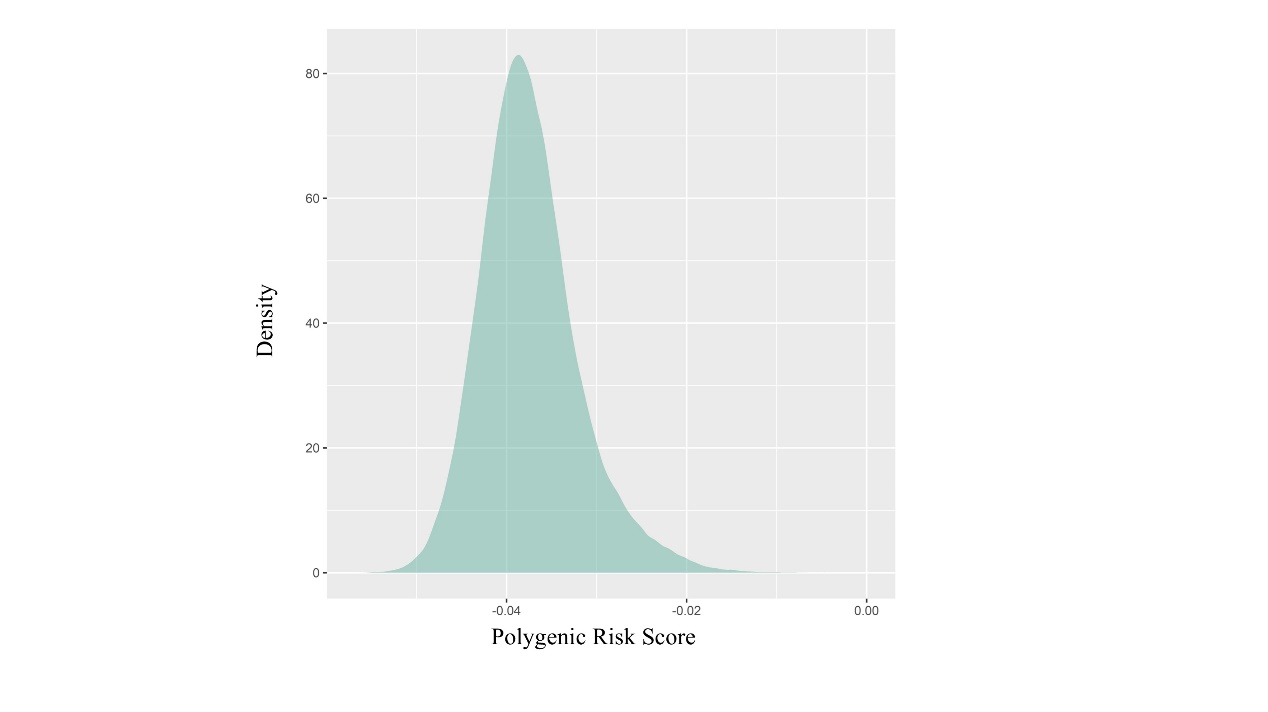


**Supplemental Figure 6 The Curve of density distribution of polygenic risk score**


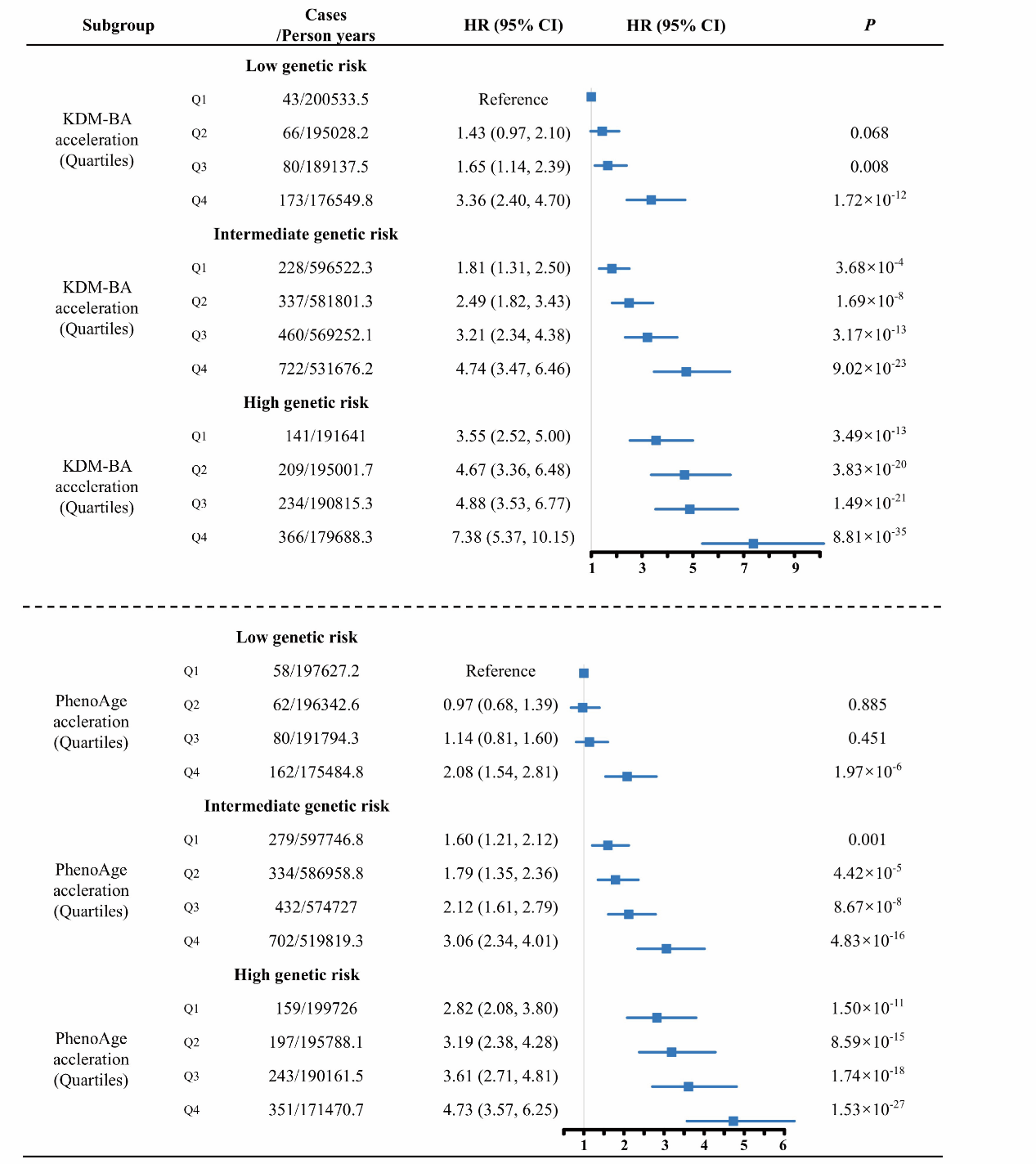


**Supplemental Figure 7 Joint effects of biological age accelerations and PRS on the risk of AS.** Model adjusted for age, sex, education level, employment status, BMI, smoking status, drinking status, physical activity, and Townsend deprivation index. HR: Hazard ratio; KDM-BA: Klemera–Doubal method biological age; Q1: Quartile 1; Q2: Quartile 2; Q3: Quartile 3; Q4: Quartile 4.


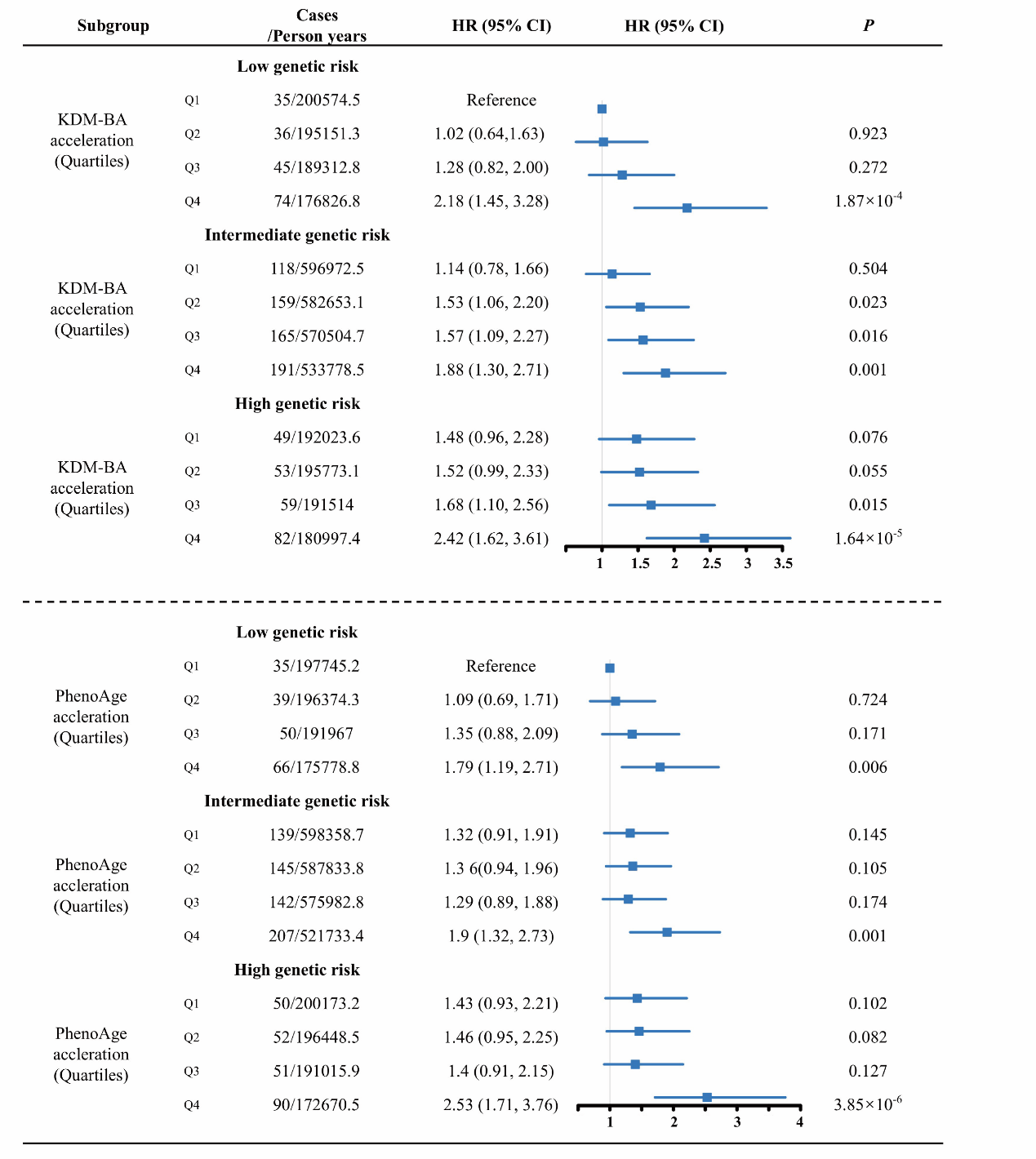


**Supplemental Figure 8 Joint effects of biological age accelerations and PRS on the risk of AR.** Model adjusted for age, sex, education level, employment status, BMI, smoking status, drinking status, physical activity, and Townsend deprivation index. HR: Hazard ratio; KDM-BA: Klemera–Doubal method biological age; Q1: Quartile 1; Q2: Quartile 2; Q3: Quartile 3; Q4: Quartile 4.


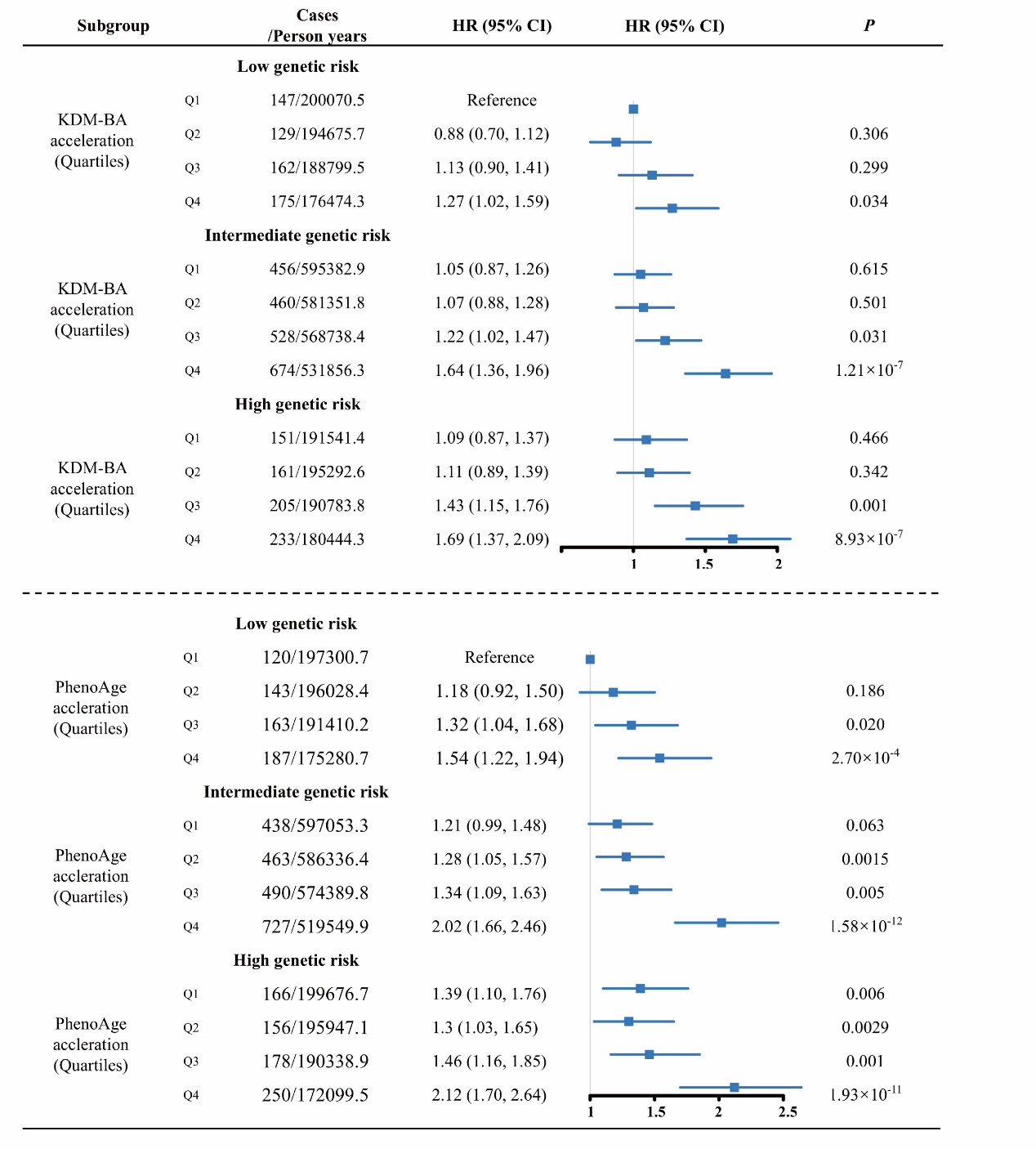


**Supplemental Figure 9 Joint effects of biological age accelerations and PRS on the risk of MR.** Model adjusted for age, sex, education level, employment status, BMI, smoking status, drinking status, physical activity, and Townsend deprivation index. HR: Hazard ratio; KDM-BA: Klemera–Doubal method biological age; Q1: Quartile 1; Q2: Quartile 2; Q3: Quartile 3; Q4: Quartile 4.


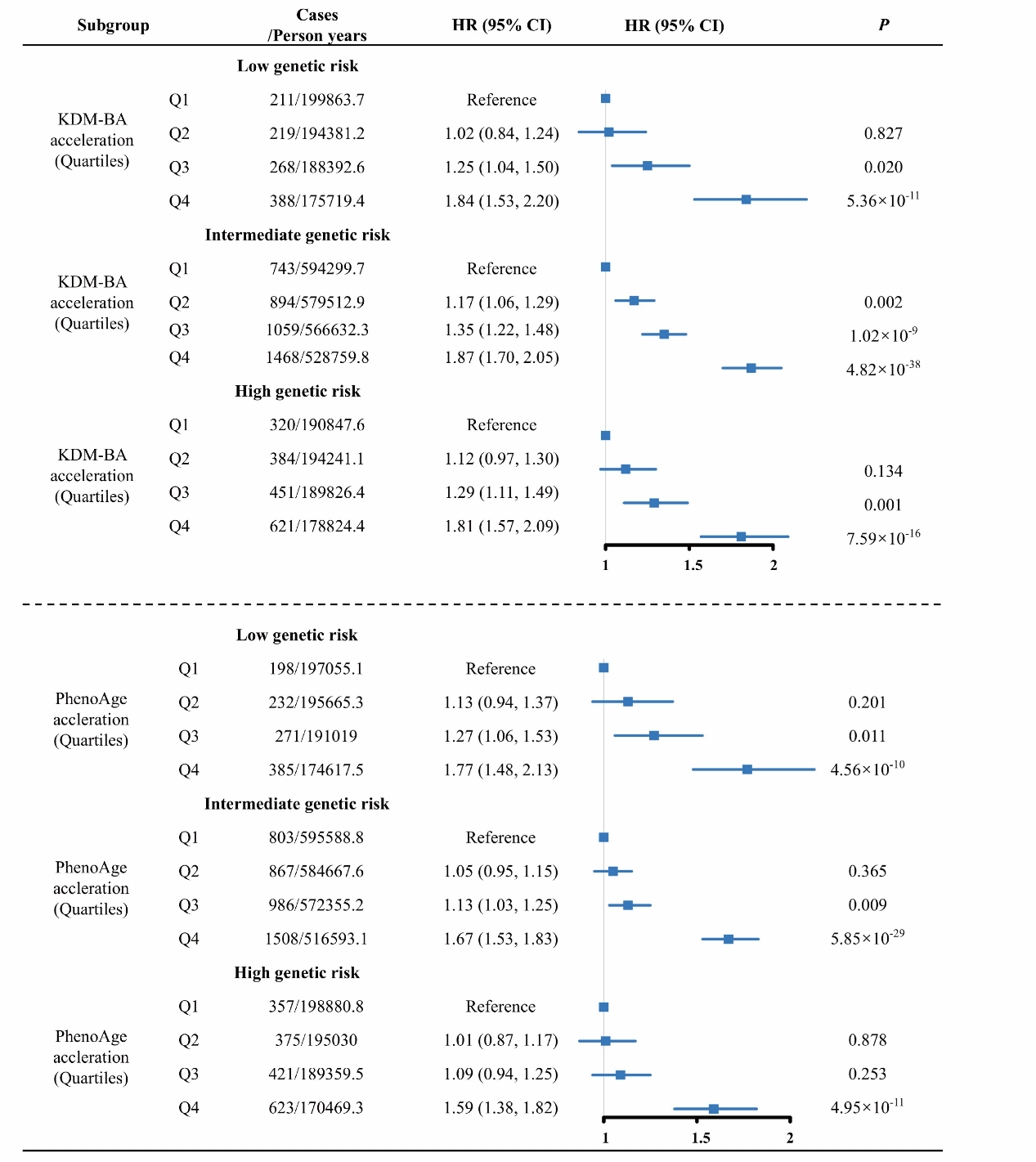


**Supplemental Figure 10 Associations of biological age accelerations with incident VHD in different genetic risk backgrounds** Model adjusted for age, sex, education level, employment status, BMI, smoking status, drinking status, physical activity, and Townsend deprivation index. HR: Hazard ratio; KDM-BA: Klemera–Doubal method biological age; Q1: Quartile 1; Q2: Quartile 2; Q3: Quartile 3; Q4: Quartile 4.


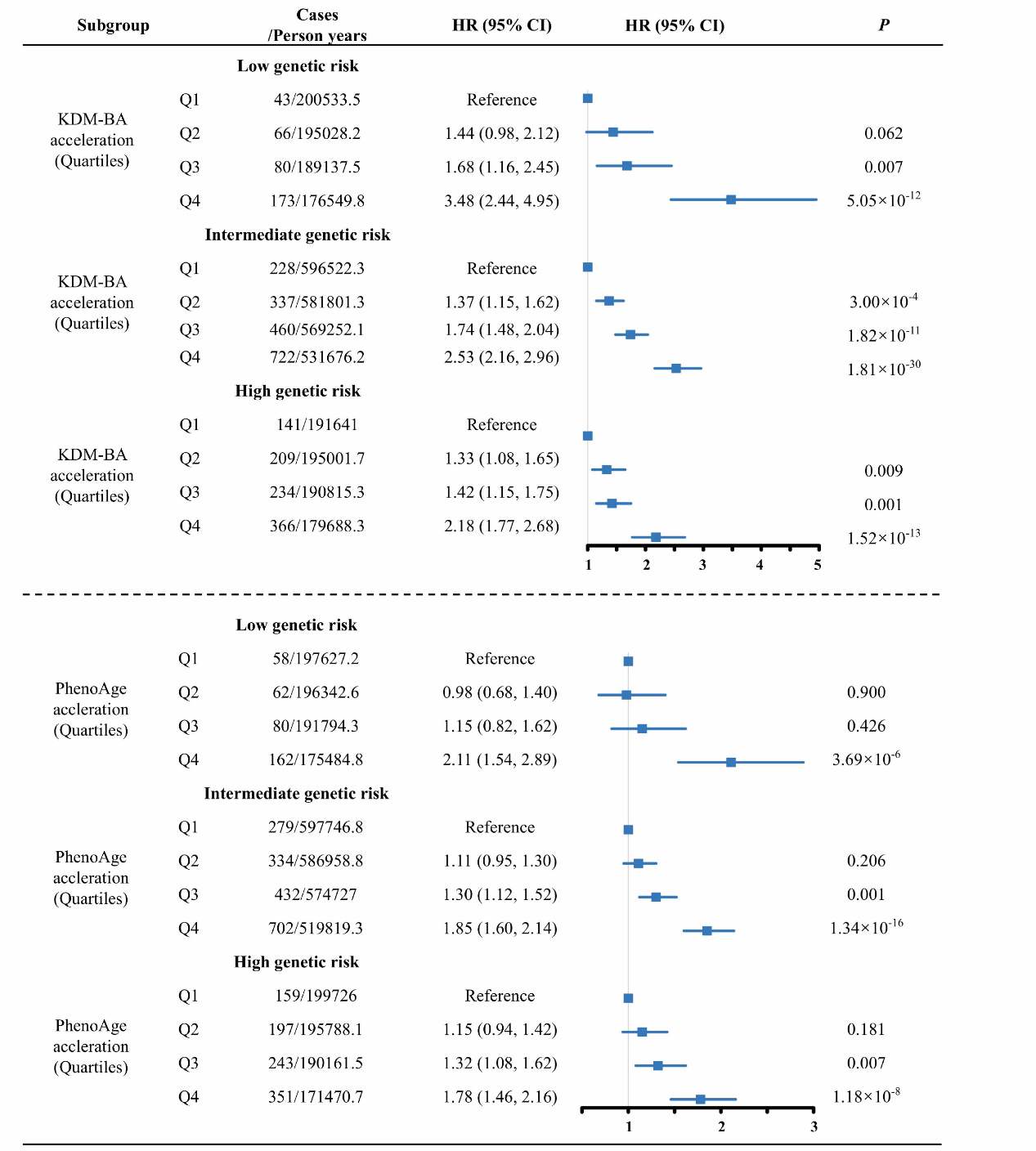


**Supplemental Figure 11 Associations of biological age accelerations with incident AS in different genetic risk backgrounds** Model adjusted for age, sex, education level, employment status, BMI, smoking status, drinking status, physical activity, and Townsend deprivation index. HR: Hazard ratio; KDM-BA: Klemera–Doubal method biological age; Q1: Quartile 1; Q2: Quartile 2; Q3: Quartile 3; Q4: Quartile 4.

**
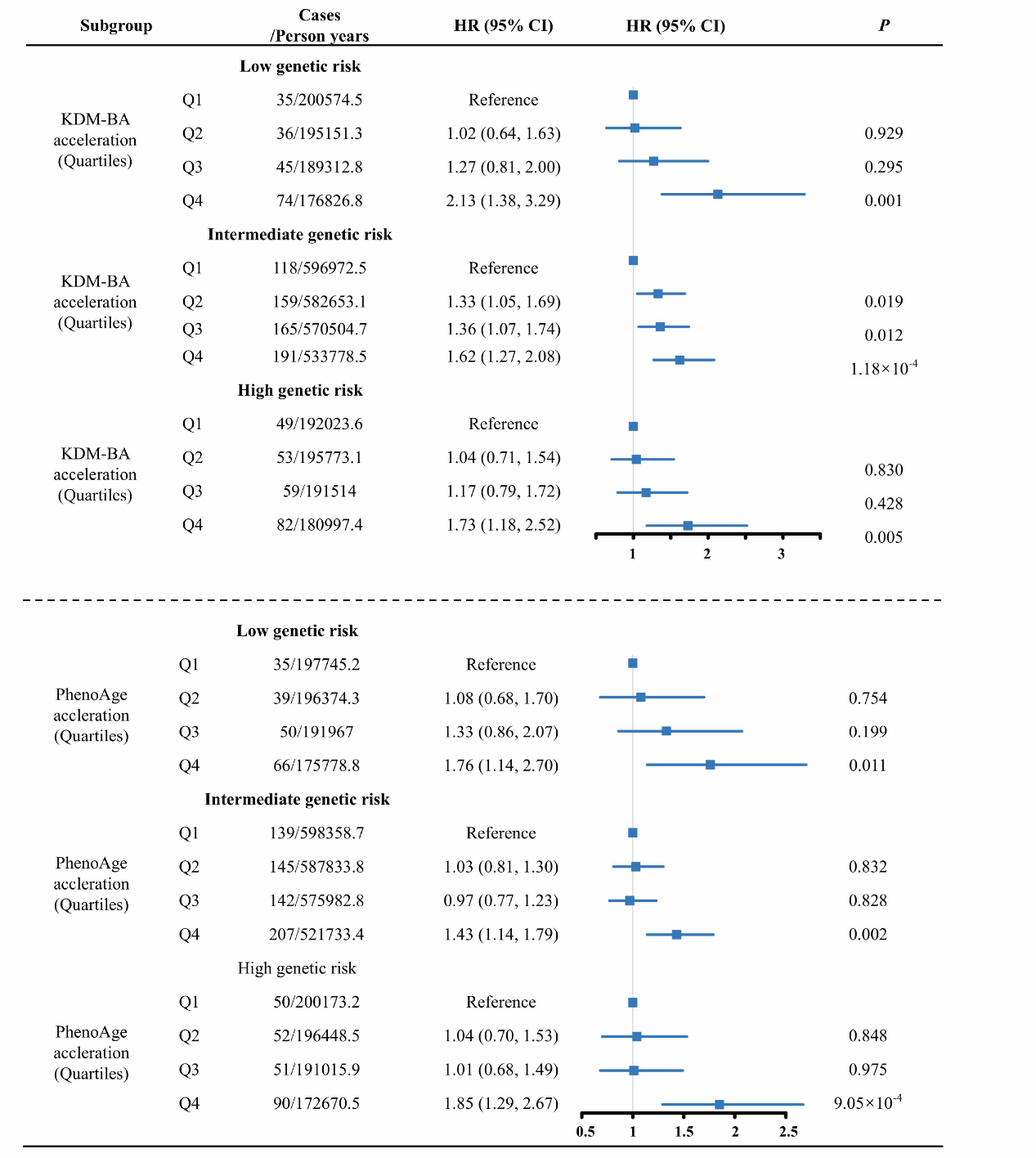
**

**Supplemental Figure 12 Associations of biological age accelerations with incident AR in different genetic risk backgrounds** Model adjusted for age, sex, education level, employment status, BMI, smoking status, drinking status, physical activity, and Townsend deprivation index. HR: Hazard ratio; KDM-BA: Klemera–Doubal method biological age; Q1: Quartile 1; Q2: Quartile 2; Q3: Quartile 3; Q4: Quartile 4.

**
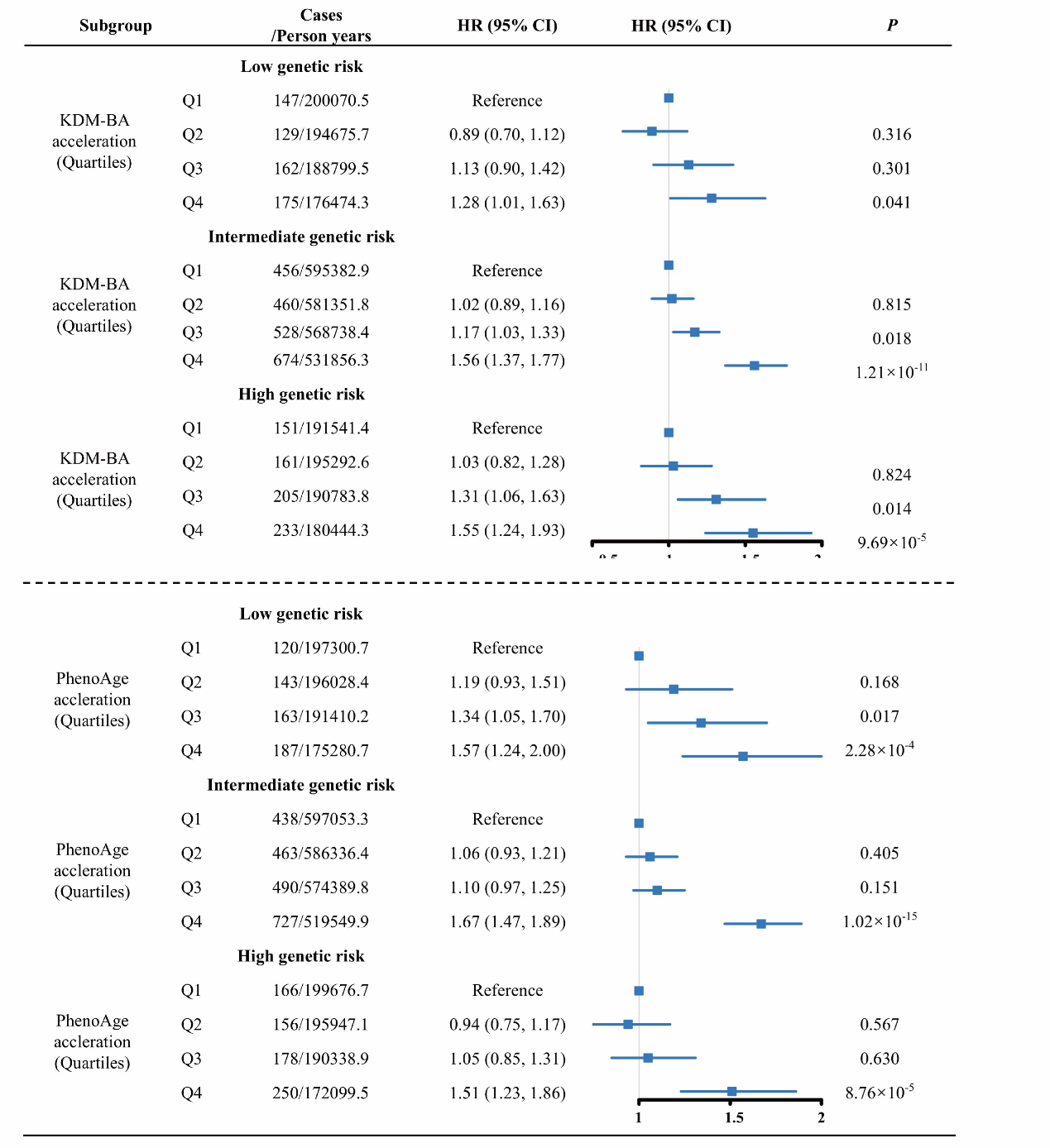
**

**Supplemental Figure 13 Associations of biological age accelerations with incident MR in different genetic risk backgrounds** Model adjusted for age, sex, education level, employment status, BMI, smoking status, drinking status, physical activity, and Townsend deprivation index. HR: Hazard ratio; KDM-BA: Klemera–Doubal method biological age; Q1: Quartile 1; Q2: Quartile 2; Q3: Quartile 3; Q4: Quartile 4.


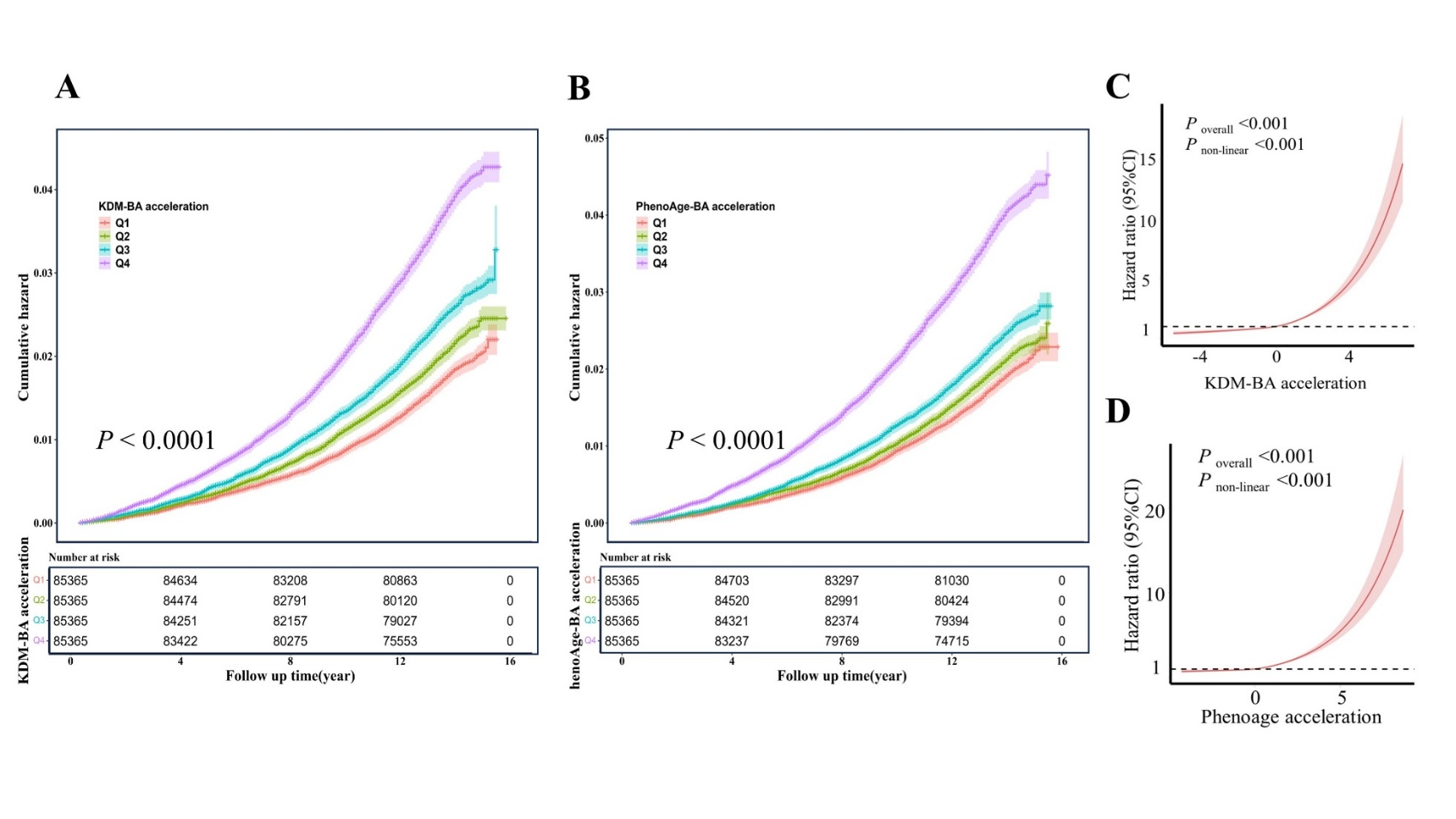


**Supplemental Figure 14 Association of biological aging with the risk VHD among individuals without missing covariate data.** (A) Cumulative VHD Incidence Difference Among KDM-BA acceleration Groups. (B) Cumulative VHD Incidence Difference Among PhenoAge acceleration Groups. (C) Association between KDM-BA acceleration and the risk of VHD using RCS. (D) Association between PhenoAge acceleration and the risk of VHD using RCS. All models adjusted for age, sex, ethnicity, education level, employment status, BMI, smoking status, drinking status, physical activity, and Townsend deprivation index. KDM-BA: Klemera–Doubal method biological age; Q1: Quartile 1; Q2: Quartile 2; Q3: Quartile 3; Q4: Quartile.


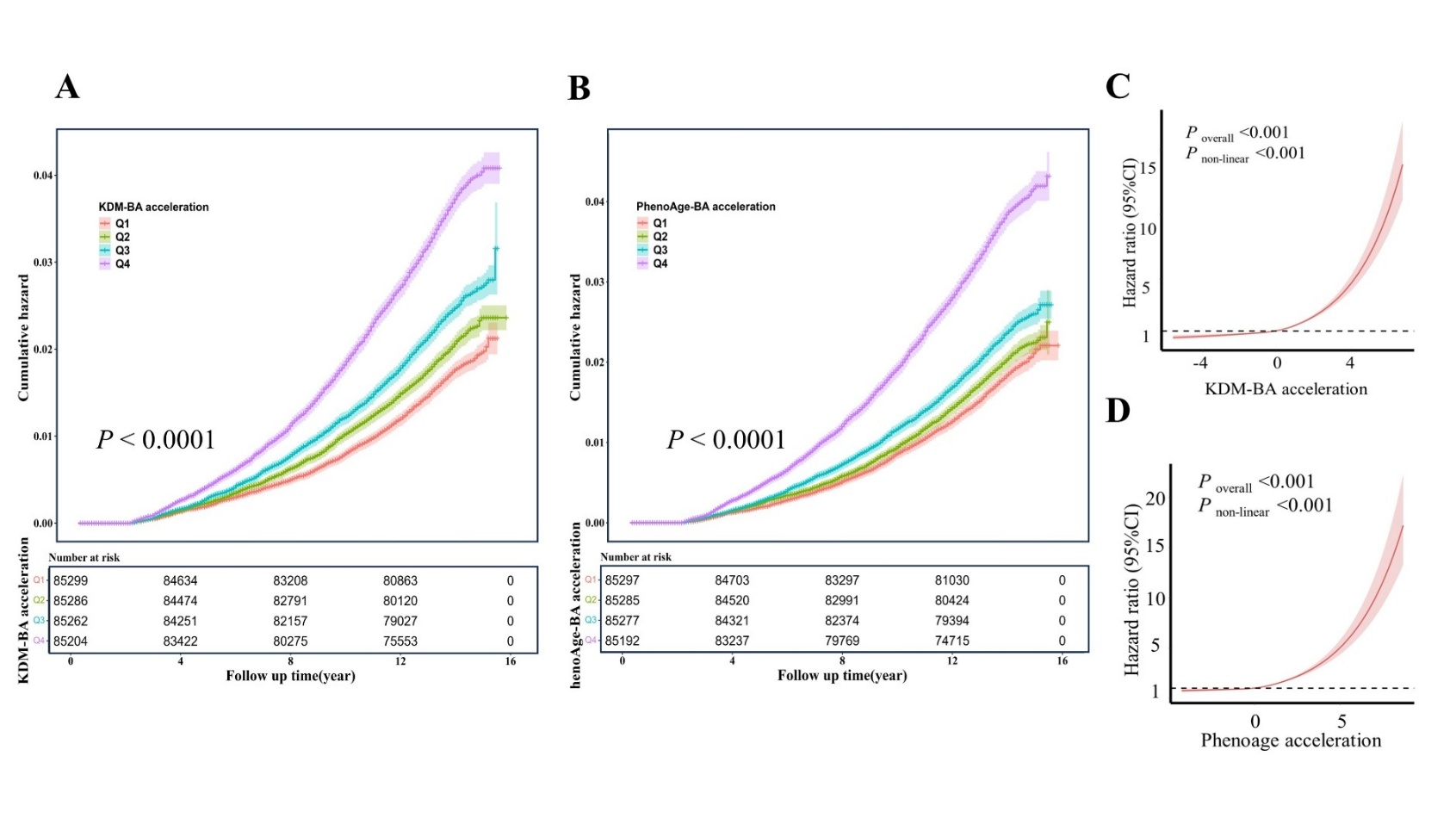


**Supplemental Figure 15 Association of biological aging with the risk VHD after excluding participants with VHD in the first 2 years.** (A) Cumulative VHD Incidence Difference Among KDM-BA acceleration Groups. (B) Cumulative VHD Incidence Difference Among PhenoAge acceleration Groups. (C) Association between KDM-BA acceleration and the risk of VHD using RCS. (D) Association between PhenoAge acceleration and the risk of VHD using RCS. All models adjusted for age, sex, ethnicity, education level, employment status, BMI, smoking status, drinking status, physical activity, and Townsend deprivation index. KDM-BA: Klemera–Doubal method biological age; Q1: Quartile 1; Q2: Quartile 2; Q3: Quartile 3; Q4: Quartile.


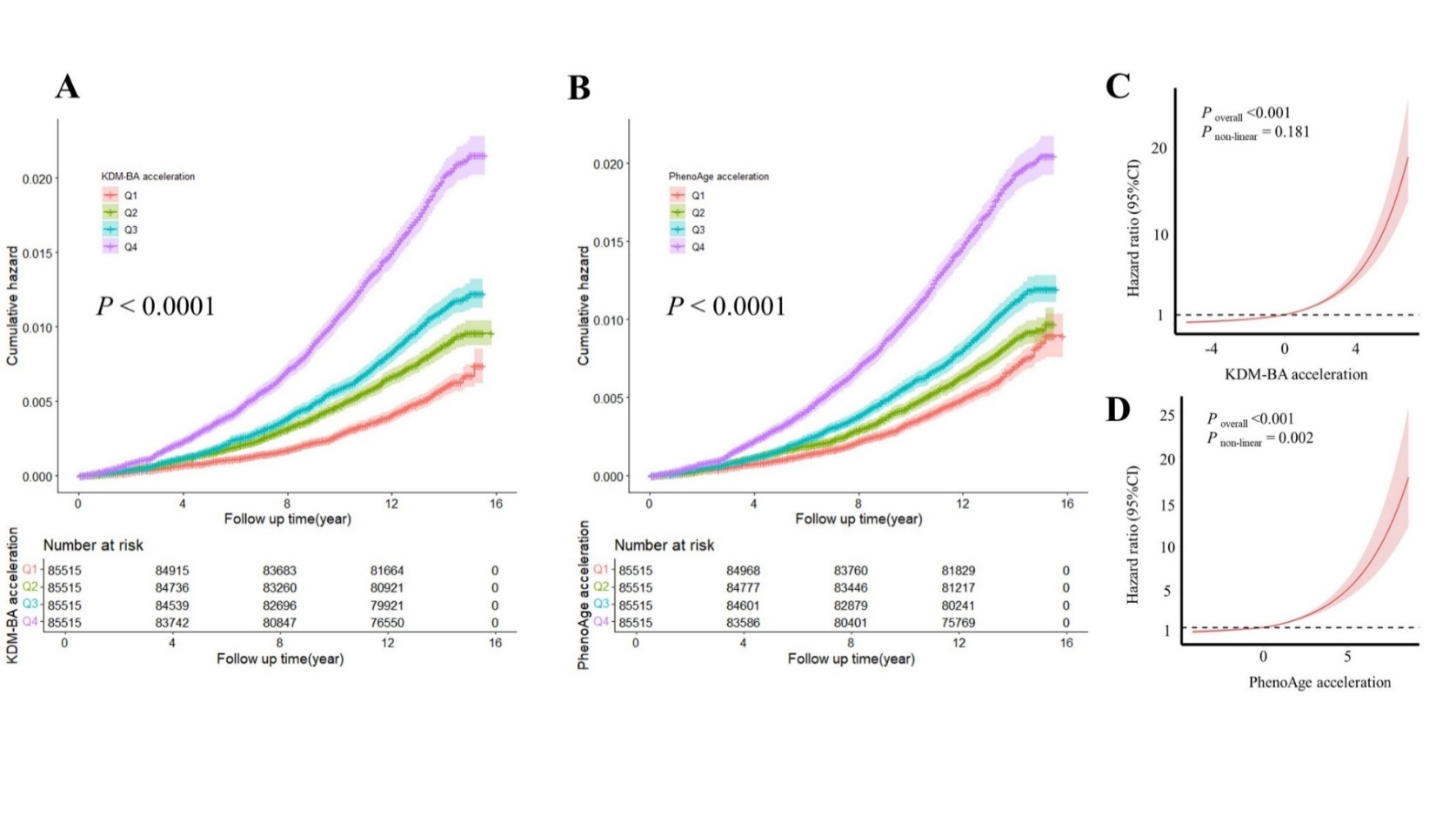


**Supplemental Figure 16 Association of biological aging with the risk VHD diagnosed by a broad definition.** (A) Cumulative VHD Incidence Difference Among KDM-BA acceleration Groups. (B) Cumulative VHD Incidence Difference Among PhenoAge acceleration Groups. (C) Association between KDM-BA acceleration and the risk of VHD using RCS. (D) Association between PhenoAge acceleration and the risk of VHD using RCS. All models adjusted for age, sex, ethnicity, education level, employment status, BMI, smoking status, drinking status, physical activity, and Townsend deprivation index. KDM-BA: Klemera–Doubal method biological age; Q1: Quartile 1; Q2: Quartile 2; Q3: Quartile 3; Q4: Quartile.


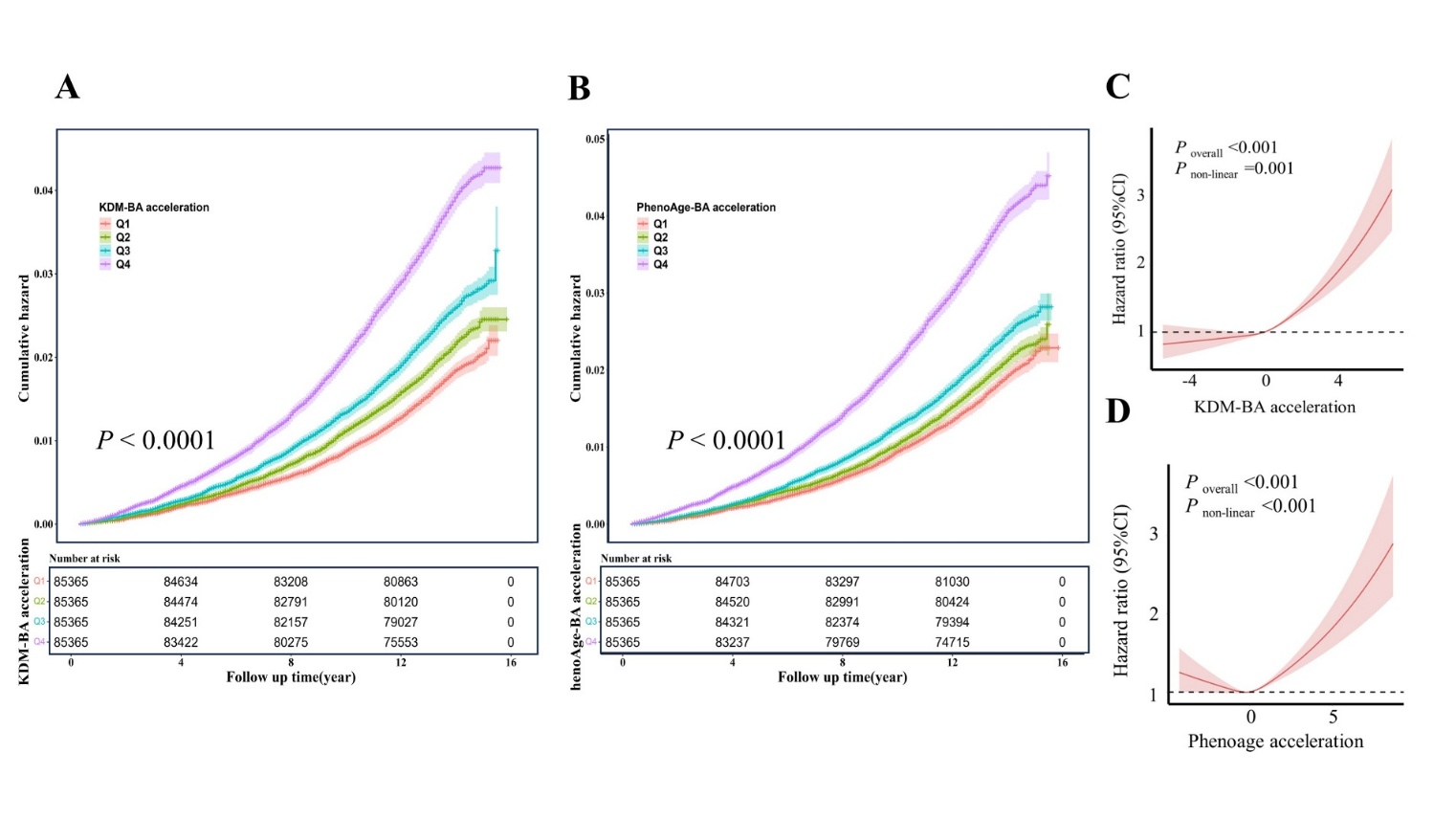


**Supplemental Figure 17 Association of biological aging with the risk VHD by additionally controlling for comorbidities.** (A) Cumulative VHD Incidence Difference Among KDM-BA acceleration Groups. (B) Cumulative VHD Incidence Difference Among PhenoAge acceleration Groups. (C) Association between KDM-BA acceleration and the risk of VHD using RCS. (D) Association between PhenoAge acceleration and the risk of VHD using RCS. All models adjusted for age, sex, ethnicity, education level, employment status, BMI, smoking status, drinking status, physical activity, Townsend deprivation index, hypertension, stroke, atrial fibrillation, chronic kidney disease, cancer and type 2 diabetes. KDM-BA: Klemera–Doubal method biological age; Q1: Quartile 1; Q2: Quartile 2; Q3: Quartile 3; Q4: Quartile.


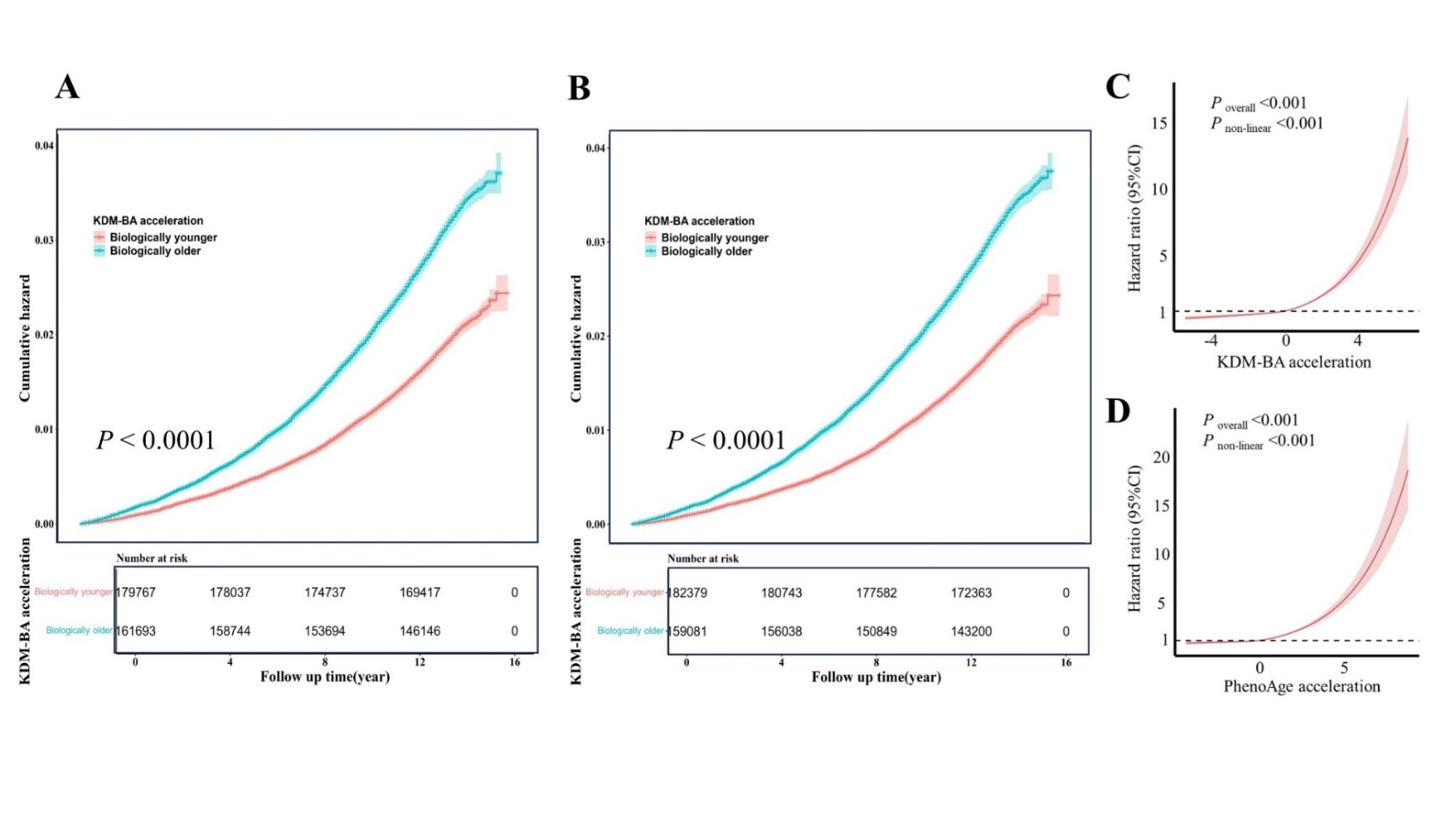


**Supplemental Figure 18 Association of biological aging with the risk VHD in biologically younger and older groups.** (A) Cumulative VHD Incidence Difference Among KDM-BA acceleration Groups. (B) Cumulative VHD Incidence Difference Among PhenoAge acceleration Groups. (C) Association between KDM-BA acceleration and the risk of VHD using RCS. (D) Association between PhenoAge acceleration and the risk of VHD using RCS. All models adjusted for age, sex, ethnicity, education level, employment status, BMI, smoking status, drinking status, physical activity, and Townsend deprivation index. KDM-BA: Klemera–Doubal method biological age; Q1: Quartile 1; Q2: Quartile 2; Q3: Quartile 3; Q4: Quartile.
